# Supplementary material for: Hospital admission with non-alcoholic fatty liver disease is associated with increased all-cause mortality independent of cardiovascular risk factors
Source: PLoS One. 2020 Oct 27;15(10):e0241357. doi: 10.1371/journal.pone.0241357 (PMC7591046; doi:10.1371/journal.pone.0241357)
Supplement: S1 Table — Baseline demographics for participants included in the study. Q-values were derived from chi-squared tests (for sex and ethnicity) or t-tests (for age) with adjustment for multiple testing using the Benjamini-Hochberg method. SD, standard deviation. (DOCX) [file pone.0241357.s001.docx]

## **S1 Table. Demographics for control, NAFL, NASH and NAFLD-cirrhosis patients.**

|  | **Control**  **(n=24,737)** | **NAFL**  **(n=994)** | **NAFL vs. Control q-value** | **NASH**  **(n=97)** | **NASH vs. Control q-value** | **NAFL vs. NASH q-value** | **Cirrhosis**  **(n=711)** | **Cirrhosis vs. Control q-value** | **Cirrhosis vs. NAFL q-value** | **Cirrhosis vs. NASH q-value** |
| --- | --- | --- | --- | --- | --- | --- | --- | --- | --- | --- |
| Mean age (SD) | 55.0 (15.4) | 51.5 (15.0) | 9.8E-12 | 52.6 (17.0) | 0.41 | 1 | 63.6 (13.5) | 2.0E-53 | 1.60E-61 | 1.1E-07 |
| Male | 10999 (44.5) | 471 (47.4) | 0.12 | 47 (48.5) | 0.71 | 1 | 338 (47.5) | 0.15 | 1 | 1 |
| Caucasian | 19761 (79.9) | 770 (77.5) | 0.12 | 80 (82.5) | 0.78 | 0.93 | 607 (85.4) | 6.5E-04 | 1.10E-04 | 0.9 |
| South Asian | 1652 ( 6.7) | 134 (13.5) | 1.3E-15 | 6 ( 6.2) | 1 | 0.45 | 37 ( 5.2) | 0.17 | 9.30E-08 | 1 |

Baseline demographics for participants included in the study. Q-values were derived from chi-squared tests (for sex and ethnicity) or t-tests (for age) with adjustment for multiple testing using the Benjamini-Hochberg method. SD, standard deviation.
