# Supplementary material for: Hospital admission with non-alcoholic fatty liver disease is associated with increased all-cause mortality independent of cardiovascular risk factors
Source: PLoS One. 2020 Oct 27;15(10):e0241357. doi: 10.1371/journal.pone.0241357 (PMC7591046; doi:10.1371/journal.pone.0241357)
Supplement: S2 Table — Crude rates of mortality, metabolic, cardiovascular, and liver-related outcomes for control, non-cirrhotic-NAFLD, and NAFLD-cirrhosis, patients during a 14-year study period. Data is given a number of events (%). Q-values were derived from chi-squared tests with adjustment for multiple testing using the Benjamini-Hochberg method. GI malignancy, gastrointestinal malignancy. (DOCX) [file pone.0241357.s002.docx]

## **S2 Table. Cardiovascular disease burden and liver-related events across the NAFLD spectrum.**

|  | **Control**  **(n=24,737)** | **NAFL**  **(n=994)** | **NAFL vs. Control q-value** | **NASH**  **(n=97)** | **NASH vs. Control q-value** | **NAFL vs. NASH q-value** | **Cirrhosis**  **(n=711)** | **Cirrhosis vs. Control q-value** | **Cirrhosis vs. NAFL q-value** | **Cirrhosis vs. NASH q-value** |
| --- | --- | --- | --- | --- | --- | --- | --- | --- | --- | --- |
| Obesity | 307 (1.2) | 83 (8.4) | 3.10E-70 | 9 (9.3) | 4.9E-10 | 1 | 27 (3.8) | 2.0E-08 | 4.2E-04 | 0.07 |
| Type 2 Diabetes | 2328 ( 9.4) | 214 (21.5) | 5.70E-35 | 21 (21.6) | 6.3E-04 | 1 | 250 (35.2) | 3.3E-110 | 2.1E-09 | 0.03 |
| Hyperlipidaemia | 2015 ( 8.1) | 128 (12.9) | 4.70E-07 | 17 (17.5) | 6.8E-03 | 0.93 | 46 ( 6.5) | 0.16 | 4.9E-05 | 1.8E-03 |
| Hypertension | 5655 (22.9) | 311 (31.3) | 2.80E-09 | 32 (33.0) | 0.09 | 1 | 225 (31.6) | 1.1E-07 | 1 | 1 |
| Ischaemic heart disease | 2951 (11.9) | 104 (10.5) | 0.24 | 11 (11.3) | 1 | 1 | 112 (15.8) | 4.0E-03 | 2.6E-03 | 0.62 |
| Myocardial infarction | 940 (3.8) | 20 (2.0) | 0.01 | 4 (4.1) | 1 | 0.93 | 21 (3.0) | 0.33 | 0.33 | 1 |
| Atrial fibrillation | 1174 ( 4.7) | 55 ( 5.5) | 0.35 | 9 ( 9.3) | 0.18 | 0.93 | 86 (12.1) | 3.3E-18 | 4.9E-06 | 0.9 |
| Congestive Heart Failure | 865 (3.5) | 34 (3.4) | 1 | 10 (10.3) | 4.5E-03 | 0.06 | 63 (8.9) | 2.7E-13 | 6.7E-06 | 1 |
| Ischaemic stroke | 498 (2.0) | 12 (1.2) | 0.15 | 0 (0.0) | 0.56 | 1 | 24 (3.4) | 0.03 | 5.4E-03 | 0.3 |
| Peripheral vascular disease | 381 (1.5) | 10 (1.0) | 0.29 | 3 (3.1) | 0.68 | 0.93 | 14 (2.0) | 0.49 | 0.19 | 1 |
| Chronic Kidney Disease | 309 (1.2) | 20 (2.0) | 0.1 | 3 (3.1) | 0.51 | 1 | 33 (4.6) | 8.8E-14 | 5.0E-03 | 1 |
| Any malignancy | 1876 ( 7.6) | 93 ( 9.4) | 0.1 | 10 (10.3) | 0.68 | 1 | 176 (24.8) | 1.2E-60 | 5.5E-17 | 9.0E-03 |
| GI malignancy | 395 ( 1.6) | 34 ( 3.4) | 4.80E-05 | 4 ( 4.1) | 0.3 | 1 | 111 (15.6) | 3.4E-151 | 5.8E-18 | 0.01 |
| Hepatic failure/decompensation | 116 ( 0.5) | 54 ( 5.4) | 5.30E-77 | 7 ( 7.2) | 6.2E-17 | 1 | 300 (42.2) | <1E-300 | 1.8E-74 | 6.8E-10 |
| Hepatocellular carcinoma | 45 ( 0.2) | 13 ( 1.3) | 9.90E-12 | 1 ( 1.0) | 0.69 | 1 | 93 (13.1) | <1E-300 | 5.1E-22 | 4.4E-03 |
| All-cause mortality | 3,635 (14.7) | 127 (12.8) | 0.15 | 22 (22.7) | 0.13 | 0.12 | 288 (40.5) | 1.9E-225 | 3.3E-90 | 3.9E-10 |

Crude rates of mortality, metabolic, cardiovascular, and liver-related outcomes for control, non-cirrhotic-NAFLD, and NAFLD-cirrhosis, patients during a 14-year study period. Data is given a number of events (%). Q-values were derived from chi-squared tests with adjustment for multiple testing using the Benjamini-Hochberg method. GI malignancy, gastrointestinal malignancy.
