# Supplementary material for: Hospital admission with non-alcoholic fatty liver disease is associated with increased all-cause mortality independent of cardiovascular risk factors
Source: PLoS One. 2020 Oct 27;15(10):e0241357. doi: 10.1371/journal.pone.0241357 (PMC7591046; doi:10.1371/journal.pone.0241357)
Supplement: S3 Table — Odds ratios for liver-related events (hepatic failure/decompensation and hepatocellular carcinoma) were calculated using multivariable logistic regression adjusted for age, sex, and ethnicity. Adjusted hazard ratios of overall mortality were calculated between control, non-cirrhotic NAFLD, and NAFLD-cirrhosis groups using Cox proportional regression. Adjustment for demographic characteristics were gender, age and ethnicity. Adjustment for cardiovascular risk factors and CVD included: obesity, type 2 diabetes mellitus, CHF, ischaemic stroke, myocardial infarction, chronic kidney disease, peripheral vascular disease, hypertension, hyperlipidaemia, ischaemic heart disease, and atrial fibrillation. Adjustment for liver-related events included: hepatocellular carcinoma, hepatic failure, oesophageal varices, portal hypertension, splenomegaly, and ascites. Control n = 25,780; NAFL n = 1,238; NASH n = 105; and Cirrhosis n = 1,235 Q-values were calculated from p-values using the Benjamini-Hochberg method. Adj. OR, adjusted odds ratio; CI, confidence interval; CVD, cardiovascular disease; HR, hazard ratio. (DOCX) [file pone.0241357.s003.docx]

## **S3 Table. Adjusted odds ratios for liver-related outcomes and adjusted mortality ratios.**

|  | **NAFL vs. Control** | | **NASH vs. Control** | | **NASH vs. NAFL** | | **Cirrhosis vs. Control** | | **Cirrhosis vs. NAFL** | | **Cirrhosis vs. NASH** | |
| --- | --- | --- | --- | --- | --- | --- | --- | --- | --- | --- | --- | --- |
|  | **Adj. OR (95% CI)** | **q-value** | **Adj. OR (95% CI)** | **q-value** | **Adj. OR (95% CI)** | **q-value** | **Adj. OR (95% CI)** | **q-value** | **Adj. OR (95% CI)** | **q-value** | **Adj. OR (95% CI)** | **q-value** |
| Hepatic failure/ decompensation | 2.6 (2.2-2.9) | 1.20E-51 | 2.9 (2.0-3.6) | 9.00E-12 | 0.3 (0.6-1.0) | 0.6 | 4.9 (4.7-5.2) | <1E-300 | 2.4 (2.1-2.8) | 4.10E-46 | 2.2 1.5-3.1) | 2.90E-07 |
| Hepatocellular carcinoma | 2.2 (1.5-2.8) | 2.80E-11 | 1.9 (1.0-3.4) | 0.08 | 0.3 (3.2-1.3) | 0.76 | 4.2 (3.8-4.6) | 8.30E-107 | 1.9 ( 1.4-2.6) | 4.30E-10 | 2.3 (0.7-5.2) | 0.02 |
|  | **HR (95% CI)** | **q-value** | **HR (95% CI)** | **q-value** | **HR (95% CI)** | **q-value** | **HR (95% CI)** | **q-value** | **HR (95% CI)** | **q-value** | **HR (95% CI)** | **q-value** |
| Mortality adjusted for demographic characteristics | 1.2 (1.0-1.5) | 0.06 | 2.1 (1.4-3.1) | 2.30E-03 | 1.6 (1.0-2.6) | 0.12 | 3.8 (3.4-4.2) | 8.30E-146 | 4.0 (3.2-4.9) | 1.80E-38 | 2.2 (1.5-3.5) | 8.20E-04 |
| Mortality adjusted for demographics, metabolic risk factors, and CVD | 1.2 (1.0-1.4) | 0.17 | 1.6 (1.1-2.5) | 0.05 | 1.6 (1.0-2.6) | 0.12 | 3.2 (2.9-3.6) | 8.30E-107 | 3.7 (3.0-4.6) | 1.10E-33 | 2.1 (1.4-3.3) | 1.40E-03 |
| Mortality adjusted for demographics and liver-related events | 1.0 (0.8-1.2) | 0.93 | 1.5 (1.0-2.3) | 0.08 | 1.5 (1.0-2.5) | 0.12 | 2.4 (2.1-2.8) | 2.80E-31 | 3.2 (2.6-4.0) | 1.70E-24 | 1.9 (1.2-3.0) | 5.40E-03 |
| Mortality adjusted for demographics, metabolic risk factors, CVD, and liver-related events | 1.0 (0.8-1.2) | 0.91 | 1.4 (0.9-2.1) | 0.15 | 1.5 (1.0-2.5) | 0.12 | 2.1 (1.8-2.4) | 9.00E-23 | 3.0 (2.4-3.8) | 7.30E-22 | 1.9 (1.2-2.9) | 6.30E-03 |

Odds ratios for liver-related events (hepatic failure/decompensation and hepatocellular carcinoma) were calculated using multivariable logistic regression adjusted for age, sex, and ethnicity. Adjusted hazard ratios of overall mortality were calculated between control, non-cirrhotic NAFLD, and NAFLD-cirrhosis groups using Cox proportional regression. Adjustment for demographic characteristics were gender, age and ethnicity. Adjustment for cardiovascular risk factors and CVD included: obesity, type 2 diabetes mellitus, CHF, ischaemic stroke, myocardial infarction, chronic kidney disease, peripheral vascular disease, hypertension, hyperlipidaemia, ischaemic heart disease, and atrial fibrillation. Adjustment for liver-related events included: hepatocellular carcinoma, hepatic failure, oesophageal varices, portal hypertension, splenomegaly, and ascites. Control n=25,780; NAFL n=1,238; NASH n=105; and Cirrhosis n=1,235 Q-values were calculated from p-values using the Benjamini-Hochberg method. Adj. OR, adjusted odds ratio; CI, confidence interval; CVD, cardiovascular disease; HR, hazard ratio.
