# Supplementary material for: Hospital admission with non-alcoholic fatty liver disease is associated with increased all-cause mortality independent of cardiovascular risk factors
Source: PLoS One. 2020 Oct 27;15(10):e0241357. doi: 10.1371/journal.pone.0241357 (PMC7591046; doi:10.1371/journal.pone.0241357)
Supplement: S4 Table — Adjusted hazard ratios of overall mortality were calculated between control, non-cirrhotic NAFLD, and NAFLD-cirrhosis groups using Cox proportional regression after removal of all participants with liver-related events. Adjustment for demographic characteristics were gender, age and ethnicity. Adjustment for cardiovascular risk factors and CVD included: obesity, type 2 diabetes mellitus, CHF, ischaemic stroke, myocardial infarction, chronic kidney disease, peripheral vascular disease, hypertension, hyperlipidaemia, ischaemic heart disease, and atrial fibrillation. Control n = 25,551, NAFLD n = 1,241, and cirrhosis n = 591. Q-values were calculated from p-values using the Benjamini-Hochberg method. CI, confidence interval; CVD, cardiovascular disease; HR, hazard ratio. (DOCX) [file pone.0241357.s004.docx]

## **Table S4. Adjusted mortality ratios for patients with no liver-related events.**

|  | **NAFLD vs. Control** | | **Cirrhosis vs. Control** | | **Cirrhosis vs. NAFLD** | |
| --- | --- | --- | --- | --- | --- | --- |
|  | **HR (95% CI)** | **q-value** | **HR (95% CI)** | **q-value** | **HR (95% CI)** | **q-value** |
| Mortality adjusted for demographic characteristics | 1.2 (1.0-1.4) | 0.08 | 3.1 (2.6-3.6) | 9.70E-46 | 2.9 (2.3-3.7) | 2.00E-18 |
| Mortality adjusted for demographics, metabolic risk factors, and CVD | 1.1 (0.9-1.3) | 0.23 | 2.6 (2.2-3.0) | 1.10E-32 | 2.8 (2.2-3.5) | 1.90E-16 |

Adjusted hazard ratios of overall mortality were calculated between control, non-cirrhotic NAFLD, and NAFLD-cirrhosis groups using Cox proportional regression after removal of all participants with liver-related events. Adjustment for demographic characteristics were gender, age and ethnicity. Adjustment for cardiovascular risk factors and CVD included: obesity, type 2 diabetes mellitus, CHF, ischaemic stroke, myocardial infarction, chronic kidney disease, peripheral vascular disease, hypertension, hyperlipidaemia, ischaemic heart disease, and atrial fibrillation. Control n=25,551, NAFLD n=1,241, and cirrhosis n=591. Q-values were calculated from p-values using the Benjamini-Hochberg method. CI, confidence interval; CVD, cardiovascular disease; HR, hazard ratio.
