# Supplementary material for: Hospital admission with non-alcoholic fatty liver disease is associated with increased all-cause mortality independent of cardiovascular risk factors
Source: PLoS One. 2020 Oct 27;15(10):e0241357. doi: 10.1371/journal.pone.0241357 (PMC7591046; doi:10.1371/journal.pone.0241357)
Supplement: S1 Fig — Survival curves showing cumulative hazard of mortality derived from four models of adjustment using Cox proportional regression. Data shows 95% CI for control n = 24,737; NAFL n = 994, NASH n = 97; and Cirrhosis n = 711. (A) adjustment for demographic characteristics only (gender, age and ethnicity). (B) adjustment for demographics plus CVD and metabolic risk factors (obesity, type 2 diabetes mellitus, CHF, ischaemic stroke, myocardial infarction, chronic kidney disease, peripheral vascular disease, hypertension, hyperlipidaemia, ischaemic heart disease, and atrial fibrillation). (C) adjustment for demographics and liver-related events (hepatocellular carcinoma, hepatic failure, oesophageal varices, portal hypertension, splenomegaly, and ascites). (D) adjustment for demographics, CVD, metabolic risk factors, and liver-related events. (DOCX) [file pone.0241357.s005.docx]

## **S1 Fig. Adjusted cumulative hazard of mortality for hospitalised controls, patients with NAFL, NASH, and NAFLD-cirrhosis.**


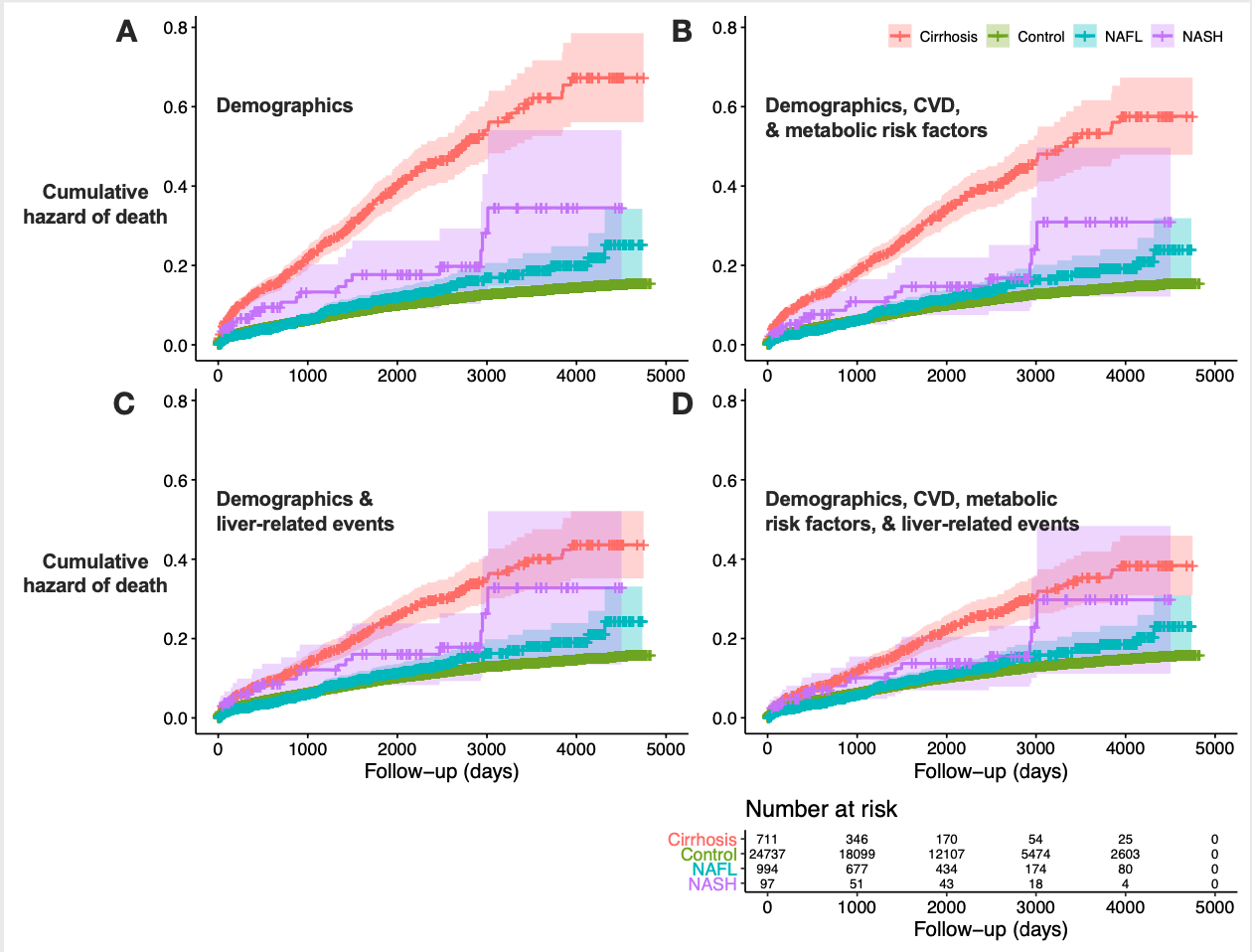


Survival curves showing cumulative hazard of mortality derived from four models of adjustment using Cox proportional regression. Data shows 95% CI for control n=24,737; NAFL n=994, NASH n=97; and Cirrhosis n=711. (A) adjustment for demographic characteristics only (gender, age and ethnicity). (B) adjustment for demographics plus CVD and metabolic risk factors (obesity, type 2 diabetes mellitus, CHF, ischaemic stroke, myocardial infarction, chronic kidney disease, peripheral vascular disease, hypertension, hyperlipidaemia, ischaemic heart disease, and atrial fibrillation). (C) adjustment for demographics and liver-related events (hepatocellular carcinoma, hepatic failure, oesophageal varices, portal hypertension, splenomegaly, and ascites). (D) adjustment for demographics, CVD, metabolic risk factors, and liver-related events.
