# Supplementary material for: Hospital admission with non-alcoholic fatty liver disease is associated with increased all-cause mortality independent of cardiovascular risk factors
Source: PLoS One. 2020 Oct 27;15(10):e0241357. doi: 10.1371/journal.pone.0241357 (PMC7591046; doi:10.1371/journal.pone.0241357)
Supplement: S1 File — (DOCX) [file pone.0241357.s006.docx]

## **Code used in analyses in R 4.0**

install.packages("tidyverse")

install.packages("stringr")

install.packages("devtools")

devtools::install_github("laresbernardo/lares")

install.packages("survival")

install.packages("survminer")

install.packages("ggplot2")

library(readxl)

df <- read_excel("dataset_v2.xlsx")

View(df)

library(stringr)

library(dplyr)

library(tidyr)

library(lares)

library(survival)

library(survminer)

df <- replaceall(df, "NULL", "NA", quiet=TRUE)

df_excl_Alc1 <- df %>% filter_at(vars(d1), all_vars(str_detect(., pattern = 'F10')))

df_excl_Alc2 <- df %>% filter_at(vars(d2), all_vars(str_detect(., pattern = 'F10')))

df_excl_Alc3 <- df %>% filter_at(vars(d3), all_vars(str_detect(., pattern = 'F10')))

df_excl_Alc4 <- df %>% filter_at(vars(d4), all_vars(str_detect(., pattern = 'F10')))

df_excl_Alc5 <- df %>% filter_at(vars(d5), all_vars(str_detect(., pattern = 'F10')))

df_excl_Alc6 <- df %>% filter_at(vars(d6), all_vars(str_detect(., pattern = 'F10')))

df_excl_Alc7 <- df %>% filter_at(vars(d7), all_vars(str_detect(., pattern = 'F10')))

df_excl_Alc8 <- df %>% filter_at(vars(d8), all_vars(str_detect(., pattern = 'F10')))

df_excl_Alc9 <- df %>% filter_at(vars(d9), all_vars(str_detect(., pattern = 'F10')))

df_excl_Alc10 <- df %>% filter_at(vars(d10), all_vars(str_detect(., pattern = 'F10')))

df_excl_Alc11 <- df %>% filter_at(vars(d11), all_vars(str_detect(., pattern = 'F10')))

df_excl_Alc12 <- df %>% filter_at(vars(d12), all_vars(str_detect(., pattern = 'F10')))

df_excl_Alc13 <- df %>% filter_at(vars(d13), all_vars(str_detect(., pattern = 'F10')))

df_excl_Alc14 <- df %>% filter_at(vars(d14), all_vars(str_detect(., pattern = 'F10')))

df_excl_Alc15 <- df %>% filter_at(vars(d15), all_vars(str_detect(., pattern = 'F10')))

df_excl_Alc16 <- df %>% filter_at(vars(d16), all_vars(str_detect(., pattern = 'F10')))

df_excl_Alc17 <- df %>% filter_at(vars(d17), all_vars(str_detect(., pattern = 'F10')))

df_excl_Alc18 <- df %>% filter_at(vars(d18), all_vars(str_detect(., pattern = 'F10')))

df_excl_Alc19 <- df %>% filter_at(vars(d19), all_vars(str_detect(., pattern = 'F10')))

df_excl_Alc20 <- df %>% filter_at(vars(d20), all_vars(str_detect(., pattern = 'F10')))

df_excl_Alc21 <- df %>% filter_at(vars(d21), all_vars(str_detect(., pattern = 'F10')))

df_excl_Alc22 <- df %>% filter_at(vars(d22), all_vars(str_detect(., pattern = 'F10')))

df_excl_Alc23 <- df %>% filter_at(vars(d23), all_vars(str_detect(., pattern = 'F10')))

df_excl_Alc24 <- df %>% filter_at(vars(d24), all_vars(str_detect(., pattern = 'F10')))

df_excl_Alc25 <- df %>% filter_at(vars(d25), all_vars(str_detect(., pattern = 'F10')))

df_excl_Alc26 <- df %>% filter_at(vars(d26), all_vars(str_detect(., pattern = 'F10')))

df_excl_Alc27 <- df %>% filter_at(vars(d27), all_vars(str_detect(., pattern = 'F10')))

df_excl_Alc28 <- df %>% filter_at(vars(d28), all_vars(str_detect(., pattern = 'F10')))

df_excl_Alc29 <- df %>% filter_at(vars(d29), all_vars(str_detect(., pattern = 'F10')))

df_excl_Alc30 <- df %>% filter_at(vars(d30), all_vars(str_detect(., pattern = 'F10')))

df_excl_Alc31 <- df %>% filter_at(vars(d31), all_vars(str_detect(., pattern = 'F10')))

df_excl_Alc32 <- df %>% filter_at(vars(d32), all_vars(str_detect(., pattern = 'F10')))

df_excl_Alc33 <- df %>% filter_at(vars(d33), all_vars(str_detect(., pattern = 'F10')))

df_excl_Alc34 <- df %>% filter_at(vars(d34), all_vars(str_detect(., pattern = 'F10')))

df_excl_AlcLiv1 <- df %>% filter_at(vars(d1), all_vars(str_detect(., pattern = 'K70')))

df_excl_AlcLiv2 <- df %>% filter_at(vars(d2), all_vars(str_detect(., pattern = 'F10')))

df_excl_AlcLiv3 <- df %>% filter_at(vars(d3), all_vars(str_detect(., pattern = 'F10')))

df_excl_AlcLiv4 <- df %>% filter_at(vars(d4), all_vars(str_detect(., pattern = 'F10')))

df_excl_AlcLiv5 <- df %>% filter_at(vars(d5), all_vars(str_detect(., pattern = 'F10')))

df_excl_AlcLiv6 <- df %>% filter_at(vars(d6), all_vars(str_detect(., pattern = 'F10')))

df_excl_AlcLiv7 <- df %>% filter_at(vars(d7), all_vars(str_detect(., pattern = 'F10')))

df_excl_AlcLiv8 <- df %>% filter_at(vars(d8), all_vars(str_detect(., pattern = 'F10')))

df_excl_AlcLiv9 <- df %>% filter_at(vars(d9), all_vars(str_detect(., pattern = 'F10')))

df_excl_AlcLiv10 <- df %>% filter_at(vars(d10), all_vars(str_detect(., pattern = 'F10')))

df_excl_AlcLiv11 <- df %>% filter_at(vars(d11), all_vars(str_detect(., pattern = 'F10')))

df_excl_AlcLiv12 <- df %>% filter_at(vars(d12), all_vars(str_detect(., pattern = 'F10')))

df_excl_AlcLiv13 <- df %>% filter_at(vars(d13), all_vars(str_detect(., pattern = 'F10')))

df_excl_AlcLiv14 <- df %>% filter_at(vars(d14), all_vars(str_detect(., pattern = 'F10')))

df_excl_AlcLiv15 <- df %>% filter_at(vars(d15), all_vars(str_detect(., pattern = 'F10')))

df_excl_AlcLiv16 <- df %>% filter_at(vars(d16), all_vars(str_detect(., pattern = 'F10')))

df_excl_AlcLiv17 <- df %>% filter_at(vars(d17), all_vars(str_detect(., pattern = 'F10')))

df_excl_AlcLiv18 <- df %>% filter_at(vars(d18), all_vars(str_detect(., pattern = 'F10')))

df_excl_AlcLiv19 <- df %>% filter_at(vars(d19), all_vars(str_detect(., pattern = 'F10')))

df_excl_AlcLiv20 <- df %>% filter_at(vars(d20), all_vars(str_detect(., pattern = 'F10')))

df_excl_AlcLiv21 <- df %>% filter_at(vars(d21), all_vars(str_detect(., pattern = 'F10')))

df_excl_AlcLiv22 <- df %>% filter_at(vars(d22), all_vars(str_detect(., pattern = 'F10')))

df_excl_AlcLiv23 <- df %>% filter_at(vars(d23), all_vars(str_detect(., pattern = 'F10')))

df_excl_AlcLiv24 <- df %>% filter_at(vars(d24), all_vars(str_detect(., pattern = 'F10')))

df_excl_AlcLiv25 <- df %>% filter_at(vars(d25), all_vars(str_detect(., pattern = 'F10')))

df_excl_AlcLiv26 <- df %>% filter_at(vars(d26), all_vars(str_detect(., pattern = 'F10')))

df_excl_AlcLiv27 <- df %>% filter_at(vars(d27), all_vars(str_detect(., pattern = 'F10')))

df_excl_AlcLiv28 <- df %>% filter_at(vars(d28), all_vars(str_detect(., pattern = 'F10')))

df_excl_AlcLiv29 <- df %>% filter_at(vars(d29), all_vars(str_detect(., pattern = 'F10')))

df_excl_AlcLiv30 <- df %>% filter_at(vars(d30), all_vars(str_detect(., pattern = 'F10')))

df_excl_AlcLiv31 <- df %>% filter_at(vars(d31), all_vars(str_detect(., pattern = 'F10')))

df_excl_AlcLiv32 <- df %>% filter_at(vars(d32), all_vars(str_detect(., pattern = 'F10')))

df_excl_AlcLiv33 <- df %>% filter_at(vars(d33), all_vars(str_detect(., pattern = 'F10')))

df_excl_AlcLiv34 <- df %>% filter_at(vars(d34), all_vars(str_detect(., pattern = 'F10')))

df_excl_PBC1 <- df %>% filter_at(vars(d1), all_vars(str_detect(., pattern = 'K743')))

df_excl_PBC2 <- df %>% filter_at(vars(d2), all_vars(str_detect(., pattern = 'K743')))

df_excl_PBC3 <- df %>% filter_at(vars(d3), all_vars(str_detect(., pattern = 'K743')))

df_excl_PBC4 <- df %>% filter_at(vars(d4), all_vars(str_detect(., pattern = 'K743')))

df_excl_PBC5 <- df %>% filter_at(vars(d5), all_vars(str_detect(., pattern = 'K743')))

df_excl_PBC6 <- df %>% filter_at(vars(d6), all_vars(str_detect(., pattern = 'K743')))

df_excl_PBC7 <- df %>% filter_at(vars(d7), all_vars(str_detect(., pattern = 'K743')))

df_excl_PBC8 <- df %>% filter_at(vars(d8), all_vars(str_detect(., pattern = 'K743')))

df_excl_PBC9 <- df %>% filter_at(vars(d9), all_vars(str_detect(., pattern = 'K743')))

df_excl_PBC10 <- df %>% filter_at(vars(d10), all_vars(str_detect(., pattern = 'K743')))

df_excl_PBC11 <- df %>% filter_at(vars(d11), all_vars(str_detect(., pattern = 'K743')))

df_excl_PBC12 <- df %>% filter_at(vars(d12), all_vars(str_detect(., pattern = 'K743')))

df_excl_PBC13 <- df %>% filter_at(vars(d13), all_vars(str_detect(., pattern = 'K743')))

df_excl_PBC14 <- df %>% filter_at(vars(d14), all_vars(str_detect(., pattern = 'K743')))

df_excl_PBC15 <- df %>% filter_at(vars(d15), all_vars(str_detect(., pattern = 'K743')))

df_excl_PBC16 <- df %>% filter_at(vars(d16), all_vars(str_detect(., pattern = 'K743')))

df_excl_PBC17 <- df %>% filter_at(vars(d17), all_vars(str_detect(., pattern = 'K743')))

df_excl_PBC18 <- df %>% filter_at(vars(d18), all_vars(str_detect(., pattern = 'K743')))

df_excl_PBC19 <- df %>% filter_at(vars(d19), all_vars(str_detect(., pattern = 'K743')))

df_excl_PBC20 <- df %>% filter_at(vars(d20), all_vars(str_detect(., pattern = 'K743')))

df_excl_PBC21 <- df %>% filter_at(vars(d21), all_vars(str_detect(., pattern = 'K743')))

df_excl_PBC22 <- df %>% filter_at(vars(d22), all_vars(str_detect(., pattern = 'K743')))

df_excl_PBC23 <- df %>% filter_at(vars(d23), all_vars(str_detect(., pattern = 'K743')))

df_excl_PBC24 <- df %>% filter_at(vars(d24), all_vars(str_detect(., pattern = 'K743')))

df_excl_PBC25 <- df %>% filter_at(vars(d25), all_vars(str_detect(., pattern = 'K743')))

df_excl_PBC26 <- df %>% filter_at(vars(d26), all_vars(str_detect(., pattern = 'K743')))

df_excl_PBC27 <- df %>% filter_at(vars(d27), all_vars(str_detect(., pattern = 'K743')))

df_excl_PBC28 <- df %>% filter_at(vars(d28), all_vars(str_detect(., pattern = 'K743')))

df_excl_PBC29 <- df %>% filter_at(vars(d29), all_vars(str_detect(., pattern = 'K743')))

df_excl_PBC30 <- df %>% filter_at(vars(d30), all_vars(str_detect(., pattern = 'K743')))

df_excl_PBC31 <- df %>% filter_at(vars(d31), all_vars(str_detect(., pattern = 'K743')))

df_excl_PBC32 <- df %>% filter_at(vars(d32), all_vars(str_detect(., pattern = 'K743')))

df_excl_PBC33 <- df %>% filter_at(vars(d33), all_vars(str_detect(., pattern = 'K743')))

df_excl_PBC34 <- df %>% filter_at(vars(d34), all_vars(str_detect(., pattern = 'K743')))

df_excl_PSC1 <- df %>% filter_at(vars(d1), all_vars(str_detect(., pattern = 'K783')))

df_excl_PSC2 <- df %>% filter_at(vars(d2), all_vars(str_detect(., pattern = 'K783')))

df_excl_PSC3 <- df %>% filter_at(vars(d3), all_vars(str_detect(., pattern = 'K783')))

df_excl_PSC4 <- df %>% filter_at(vars(d4), all_vars(str_detect(., pattern = 'K783')))

df_excl_PSC5 <- df %>% filter_at(vars(d5), all_vars(str_detect(., pattern = 'K783')))

df_excl_PSC6 <- df %>% filter_at(vars(d6), all_vars(str_detect(., pattern = 'K783')))

df_excl_PSC7 <- df %>% filter_at(vars(d7), all_vars(str_detect(., pattern = 'K783')))

df_excl_PSC8 <- df %>% filter_at(vars(d8), all_vars(str_detect(., pattern = 'K783')))

df_excl_PSC9 <- df %>% filter_at(vars(d9), all_vars(str_detect(., pattern = 'K783')))

df_excl_PSC10 <- df %>% filter_at(vars(d10), all_vars(str_detect(., pattern = 'K783')))

df_excl_PSC11 <- df %>% filter_at(vars(d11), all_vars(str_detect(., pattern = 'K783')))

df_excl_PSC12 <- df %>% filter_at(vars(d12), all_vars(str_detect(., pattern = 'K783')))

df_excl_PSC13 <- df %>% filter_at(vars(d13), all_vars(str_detect(., pattern = 'K783')))

df_excl_PSC14 <- df %>% filter_at(vars(d14), all_vars(str_detect(., pattern = 'K783')))

df_excl_PSC15 <- df %>% filter_at(vars(d15), all_vars(str_detect(., pattern = 'K783')))

df_excl_PSC16 <- df %>% filter_at(vars(d16), all_vars(str_detect(., pattern = 'K783')))

df_excl_PSC17 <- df %>% filter_at(vars(d17), all_vars(str_detect(., pattern = 'K783')))

df_excl_PSC18 <- df %>% filter_at(vars(d18), all_vars(str_detect(., pattern = 'K783')))

df_excl_PSC19 <- df %>% filter_at(vars(d19), all_vars(str_detect(., pattern = 'K783')))

df_excl_PSC20 <- df %>% filter_at(vars(d20), all_vars(str_detect(., pattern = 'K783')))

df_excl_PSC21 <- df %>% filter_at(vars(d21), all_vars(str_detect(., pattern = 'K783')))

df_excl_PSC22 <- df %>% filter_at(vars(d22), all_vars(str_detect(., pattern = 'K783')))

df_excl_PSC23 <- df %>% filter_at(vars(d23), all_vars(str_detect(., pattern = 'K783')))

df_excl_PSC24 <- df %>% filter_at(vars(d24), all_vars(str_detect(., pattern = 'K783')))

df_excl_PSC25 <- df %>% filter_at(vars(d25), all_vars(str_detect(., pattern = 'K783')))

df_excl_PSC26 <- df %>% filter_at(vars(d26), all_vars(str_detect(., pattern = 'K783')))

df_excl_PSC27 <- df %>% filter_at(vars(d27), all_vars(str_detect(., pattern = 'K783')))

df_excl_PSC28 <- df %>% filter_at(vars(d28), all_vars(str_detect(., pattern = 'K783')))

df_excl_PSC29 <- df %>% filter_at(vars(d29), all_vars(str_detect(., pattern = 'K783')))

df_excl_PSC30 <- df %>% filter_at(vars(d30), all_vars(str_detect(., pattern = 'K783')))

df_excl_PSC31 <- df %>% filter_at(vars(d31), all_vars(str_detect(., pattern = 'K783')))

df_excl_PSC32 <- df %>% filter_at(vars(d32), all_vars(str_detect(., pattern = 'K783')))

df_excl_PSC33 <- df %>% filter_at(vars(d33), all_vars(str_detect(., pattern = 'K783')))

df_excl_PSC34 <- df %>% filter_at(vars(d34), all_vars(str_detect(., pattern = 'K783')))

df_excl_AIH1 <- df %>% filter_at(vars(d1), all_vars(str_detect(., pattern = 'K754')))

df_excl_AIH2 <- df %>% filter_at(vars(d2), all_vars(str_detect(., pattern = 'K754')))

df_excl_AIH3 <- df %>% filter_at(vars(d3), all_vars(str_detect(., pattern = 'K754')))

df_excl_AIH4 <- df %>% filter_at(vars(d4), all_vars(str_detect(., pattern = 'K754')))

df_excl_AIH5 <- df %>% filter_at(vars(d5), all_vars(str_detect(., pattern = 'K754')))

df_excl_AIH6 <- df %>% filter_at(vars(d6), all_vars(str_detect(., pattern = 'K754')))

df_excl_AIH7 <- df %>% filter_at(vars(d7), all_vars(str_detect(., pattern = 'K754')))

df_excl_AIH8 <- df %>% filter_at(vars(d8), all_vars(str_detect(., pattern = 'K754')))

df_excl_AIH9 <- df %>% filter_at(vars(d9), all_vars(str_detect(., pattern = 'K754')))

df_excl_AIH10 <- df %>% filter_at(vars(d10), all_vars(str_detect(., pattern = 'K754')))

df_excl_AIH11 <- df %>% filter_at(vars(d11), all_vars(str_detect(., pattern = 'K754')))

df_excl_AIH12 <- df %>% filter_at(vars(d12), all_vars(str_detect(., pattern = 'K754')))

df_excl_AIH13 <- df %>% filter_at(vars(d13), all_vars(str_detect(., pattern = 'K754')))

df_excl_AIH14 <- df %>% filter_at(vars(d14), all_vars(str_detect(., pattern = 'K754')))

df_excl_AIH15 <- df %>% filter_at(vars(d15), all_vars(str_detect(., pattern = 'K754')))

df_excl_AIH16 <- df %>% filter_at(vars(d16), all_vars(str_detect(., pattern = 'K754')))

df_excl_AIH17 <- df %>% filter_at(vars(d17), all_vars(str_detect(., pattern = 'K754')))

df_excl_AIH18 <- df %>% filter_at(vars(d18), all_vars(str_detect(., pattern = 'K754')))

df_excl_AIH19 <- df %>% filter_at(vars(d19), all_vars(str_detect(., pattern = 'K754')))

df_excl_AIH20 <- df %>% filter_at(vars(d20), all_vars(str_detect(., pattern = 'K754')))

df_excl_AIH21 <- df %>% filter_at(vars(d21), all_vars(str_detect(., pattern = 'K754')))

df_excl_AIH22 <- df %>% filter_at(vars(d22), all_vars(str_detect(., pattern = 'K754')))

df_excl_AIH23 <- df %>% filter_at(vars(d23), all_vars(str_detect(., pattern = 'K754')))

df_excl_AIH24 <- df %>% filter_at(vars(d24), all_vars(str_detect(., pattern = 'K754')))

df_excl_AIH25 <- df %>% filter_at(vars(d25), all_vars(str_detect(., pattern = 'K754')))

df_excl_AIH26 <- df %>% filter_at(vars(d26), all_vars(str_detect(., pattern = 'K754')))

df_excl_AIH27 <- df %>% filter_at(vars(d27), all_vars(str_detect(., pattern = 'K754')))

df_excl_AIH28 <- df %>% filter_at(vars(d28), all_vars(str_detect(., pattern = 'K754')))

df_excl_AIH29 <- df %>% filter_at(vars(d29), all_vars(str_detect(., pattern = 'K754')))

df_excl_AIH30 <- df %>% filter_at(vars(d30), all_vars(str_detect(., pattern = 'K754')))

df_excl_AIH31 <- df %>% filter_at(vars(d31), all_vars(str_detect(., pattern = 'K754')))

df_excl_AIH32 <- df %>% filter_at(vars(d32), all_vars(str_detect(., pattern = 'K754')))

df_excl_AIH33 <- df %>% filter_at(vars(d33), all_vars(str_detect(., pattern = 'K754')))

df_excl_AIH34 <- df %>% filter_at(vars(d34), all_vars(str_detect(., pattern = 'K754')))

df_excl_WDHH1 <- df %>% filter_at(vars(d1), all_vars(str_detect(., pattern = 'E83')))

df_excl_WDHH2 <- df %>% filter_at(vars(d2), all_vars(str_detect(., pattern = 'K754')))

df_excl_WDHH3 <- df %>% filter_at(vars(d3), all_vars(str_detect(., pattern = 'K754')))

df_excl_WDHH4 <- df %>% filter_at(vars(d4), all_vars(str_detect(., pattern = 'K754')))

df_excl_WDHH5 <- df %>% filter_at(vars(d5), all_vars(str_detect(., pattern = 'K754')))

df_excl_WDHH6 <- df %>% filter_at(vars(d6), all_vars(str_detect(., pattern = 'K754')))

df_excl_WDHH7 <- df %>% filter_at(vars(d7), all_vars(str_detect(., pattern = 'K754')))

df_excl_WDHH8 <- df %>% filter_at(vars(d8), all_vars(str_detect(., pattern = 'K754')))

df_excl_WDHH9 <- df %>% filter_at(vars(d9), all_vars(str_detect(., pattern = 'K754')))

df_excl_WDHH10 <- df %>% filter_at(vars(d10), all_vars(str_detect(., pattern = 'K754')))

df_excl_WDHH11 <- df %>% filter_at(vars(d11), all_vars(str_detect(., pattern = 'K754')))

df_excl_WDHH12 <- df %>% filter_at(vars(d12), all_vars(str_detect(., pattern = 'K754')))

df_excl_WDHH13 <- df %>% filter_at(vars(d13), all_vars(str_detect(., pattern = 'K754')))

df_excl_WDHH14 <- df %>% filter_at(vars(d14), all_vars(str_detect(., pattern = 'K754')))

df_excl_WDHH15 <- df %>% filter_at(vars(d15), all_vars(str_detect(., pattern = 'K754')))

df_excl_WDHH16 <- df %>% filter_at(vars(d16), all_vars(str_detect(., pattern = 'K754')))

df_excl_WDHH17 <- df %>% filter_at(vars(d17), all_vars(str_detect(., pattern = 'K754')))

df_excl_WDHH18 <- df %>% filter_at(vars(d18), all_vars(str_detect(., pattern = 'K754')))

df_excl_WDHH19 <- df %>% filter_at(vars(d19), all_vars(str_detect(., pattern = 'K754')))

df_excl_WDHH20 <- df %>% filter_at(vars(d20), all_vars(str_detect(., pattern = 'K754')))

df_excl_WDHH21 <- df %>% filter_at(vars(d21), all_vars(str_detect(., pattern = 'K754')))

df_excl_WDHH22 <- df %>% filter_at(vars(d22), all_vars(str_detect(., pattern = 'K754')))

df_excl_WDHH23 <- df %>% filter_at(vars(d23), all_vars(str_detect(., pattern = 'K754')))

df_excl_WDHH24 <- df %>% filter_at(vars(d24), all_vars(str_detect(., pattern = 'K754')))

df_excl_WDHH25 <- df %>% filter_at(vars(d25), all_vars(str_detect(., pattern = 'K754')))

df_excl_WDHH26 <- df %>% filter_at(vars(d26), all_vars(str_detect(., pattern = 'K754')))

df_excl_WDHH27 <- df %>% filter_at(vars(d27), all_vars(str_detect(., pattern = 'K754')))

df_excl_WDHH28 <- df %>% filter_at(vars(d28), all_vars(str_detect(., pattern = 'K754')))

df_excl_WDHH29 <- df %>% filter_at(vars(d29), all_vars(str_detect(., pattern = 'K754')))

df_excl_WDHH30 <- df %>% filter_at(vars(d30), all_vars(str_detect(., pattern = 'K754')))

df_excl_WDHH31 <- df %>% filter_at(vars(d31), all_vars(str_detect(., pattern = 'K754')))

df_excl_WDHH32 <- df %>% filter_at(vars(d32), all_vars(str_detect(., pattern = 'K754')))

df_excl_WDHH33 <- df %>% filter_at(vars(d33), all_vars(str_detect(., pattern = 'K754')))

df_excl_WDHH34 <- df %>% filter_at(vars(d34), all_vars(str_detect(., pattern = 'K754')))

df_excl_Viral1 <- df %>% filter_at(vars(d1), all_vars(str_detect(., pattern = 'B1')))

df_excl_Viral2 <- df %>% filter_at(vars(d2), all_vars(str_detect(., pattern = 'B1')))

df_excl_Viral3 <- df %>% filter_at(vars(d3), all_vars(str_detect(., pattern = 'B1')))

df_excl_Viral4 <- df %>% filter_at(vars(d4), all_vars(str_detect(., pattern = 'B1')))

df_excl_Viral5 <- df %>% filter_at(vars(d5), all_vars(str_detect(., pattern = 'B1')))

df_excl_Viral6 <- df %>% filter_at(vars(d6), all_vars(str_detect(., pattern = 'B1')))

df_excl_Viral7 <- df %>% filter_at(vars(d7), all_vars(str_detect(., pattern = 'B1')))

df_excl_Viral8 <- df %>% filter_at(vars(d8), all_vars(str_detect(., pattern = 'B1')))

df_excl_Viral9 <- df %>% filter_at(vars(d9), all_vars(str_detect(., pattern = 'B1')))

df_excl_Viral10 <- df %>% filter_at(vars(d10), all_vars(str_detect(., pattern = 'B1')))

df_excl_Viral11 <- df %>% filter_at(vars(d11), all_vars(str_detect(., pattern = 'B1')))

df_excl_Viral12 <- df %>% filter_at(vars(d12), all_vars(str_detect(., pattern = 'B1')))

df_excl_Viral13 <- df %>% filter_at(vars(d13), all_vars(str_detect(., pattern = 'B1')))

df_excl_Viral14 <- df %>% filter_at(vars(d14), all_vars(str_detect(., pattern = 'B1')))

df_excl_Viral15 <- df %>% filter_at(vars(d15), all_vars(str_detect(., pattern = 'B1')))

df_excl_Viral16 <- df %>% filter_at(vars(d16), all_vars(str_detect(., pattern = 'B1')))

df_excl_Viral17 <- df %>% filter_at(vars(d17), all_vars(str_detect(., pattern = 'B1')))

df_excl_Viral18 <- df %>% filter_at(vars(d18), all_vars(str_detect(., pattern = 'B1')))

df_excl_Viral19 <- df %>% filter_at(vars(d19), all_vars(str_detect(., pattern = 'B1')))

df_excl_Viral20 <- df %>% filter_at(vars(d20), all_vars(str_detect(., pattern = 'B1')))

df_excl_Viral21 <- df %>% filter_at(vars(d21), all_vars(str_detect(., pattern = 'B1')))

df_excl_Viral22 <- df %>% filter_at(vars(d22), all_vars(str_detect(., pattern = 'B1')))

df_excl_Viral23 <- df %>% filter_at(vars(d23), all_vars(str_detect(., pattern = 'B1')))

df_excl_Viral24 <- df %>% filter_at(vars(d24), all_vars(str_detect(., pattern = 'B1')))

df_excl_Viral25 <- df %>% filter_at(vars(d25), all_vars(str_detect(., pattern = 'B1')))

df_excl_Viral26 <- df %>% filter_at(vars(d26), all_vars(str_detect(., pattern = 'B1')))

df_excl_Viral27 <- df %>% filter_at(vars(d27), all_vars(str_detect(., pattern = 'B1')))

df_excl_Viral28 <- df %>% filter_at(vars(d28), all_vars(str_detect(., pattern = 'B1')))

df_excl_Viral29 <- df %>% filter_at(vars(d29), all_vars(str_detect(., pattern = 'B1')))

df_excl_Viral30 <- df %>% filter_at(vars(d30), all_vars(str_detect(., pattern = 'B1')))

df_excl_Viral31 <- df %>% filter_at(vars(d31), all_vars(str_detect(., pattern = 'B1')))

df_excl_Viral32 <- df %>% filter_at(vars(d32), all_vars(str_detect(., pattern = 'B1')))

df_excl_Viral33 <- df %>% filter_at(vars(d33), all_vars(str_detect(., pattern = 'B1')))

df_excl_Viral34 <- df %>% filter_at(vars(d34), all_vars(str_detect(., pattern = 'B1')))

df_allexcld <- rbind(df_excl_Alc1, df_excl_Alc2, df_excl_Alc3, df_excl_Alc4, df_excl_Alc5, df_excl_Alc6, df_excl_Alc7, df_excl_Alc8, df_excl_Alc9, df_excl_Alc10, df_excl_Alc11, df_excl_Alc12, df_excl_Alc13, df_excl_Alc14, df_excl_Alc15, df_excl_Alc16, df_excl_Alc17, df_excl_Alc18, df_excl_Alc19, df_excl_Alc20, df_excl_Alc21, df_excl_Alc22, df_excl_Alc23, df_excl_Alc24, df_excl_Alc25, df_excl_Alc26, df_excl_Alc27, df_excl_Alc28, df_excl_Alc29, df_excl_Alc30, df_excl_Alc31, df_excl_Alc32, df_excl_Alc33, df_excl_Alc34, df_excl_AlcLiv1, df_excl_AlcLiv2, df_excl_AlcLiv3, df_excl_AlcLiv4, df_excl_AlcLiv5, df_excl_AlcLiv6, df_excl_AlcLiv7, df_excl_AlcLiv8, df_excl_AlcLiv9, df_excl_AlcLiv10, df_excl_AlcLiv11, df_excl_AlcLiv12, df_excl_AlcLiv13, df_excl_AlcLiv14, df_excl_AlcLiv15, df_excl_AlcLiv16, df_excl_AlcLiv17, df_excl_AlcLiv18, df_excl_AlcLiv19, df_excl_AlcLiv20, df_excl_AlcLiv21, df_excl_AlcLiv22, df_excl_AlcLiv23, df_excl_AlcLiv24, df_excl_AlcLiv25, df_excl_AlcLiv26, df_excl_AlcLiv27, df_excl_AlcLiv28, df_excl_AlcLiv29, df_excl_AlcLiv30, df_excl_AlcLiv31, df_excl_AlcLiv32, df_excl_AlcLiv33, df_excl_AlcLiv34, df_excl_PBC1, df_excl_PBC2, df_excl_PBC3, df_excl_PBC4, df_excl_PBC5, df_excl_PBC6, df_excl_PBC7, df_excl_PBC8, df_excl_PBC9, df_excl_PBC10, df_excl_PBC11, df_excl_PBC12, df_excl_PBC13, df_excl_PBC14, df_excl_PBC15, df_excl_PBC16, df_excl_PBC17, df_excl_PBC18, df_excl_PBC19, df_excl_PBC20, df_excl_PBC21, df_excl_PBC22, df_excl_PBC23, df_excl_PBC24, df_excl_PBC25, df_excl_PBC26, df_excl_PBC27, df_excl_PBC28, df_excl_PBC29, df_excl_PBC30, df_excl_PBC31, df_excl_PBC32, df_excl_PBC33, df_excl_PBC34, df_excl_PSC1, df_excl_PSC2, df_excl_PSC3, df_excl_PSC4, df_excl_PSC5, df_excl_PSC6, df_excl_PSC7, df_excl_PSC8, df_excl_PSC9, df_excl_PSC10, df_excl_PSC11, df_excl_PSC12, df_excl_PSC13, df_excl_PSC14, df_excl_PSC15, df_excl_PSC16, df_excl_PSC17, df_excl_PSC18, df_excl_PSC19, df_excl_PSC20, df_excl_PSC21, df_excl_PSC22, df_excl_PSC23, df_excl_PSC24, df_excl_PSC25, df_excl_PSC26, df_excl_PSC27, df_excl_PSC28, df_excl_PSC29, df_excl_PSC30, df_excl_PSC31, df_excl_PSC32, df_excl_PSC33, df_excl_PSC34, df_excl_AIH1, df_excl_AIH2, df_excl_AIH3, df_excl_AIH4, df_excl_AIH5, df_excl_AIH6, df_excl_AIH7, df_excl_AIH8, df_excl_AIH9, df_excl_AIH10, df_excl_AIH11, df_excl_AIH12, df_excl_AIH13, df_excl_AIH14, df_excl_AIH15, df_excl_AIH16, df_excl_AIH17, df_excl_AIH18, df_excl_AIH19, df_excl_AIH20, df_excl_AIH21, df_excl_AIH22, df_excl_AIH23, df_excl_AIH24, df_excl_AIH25, df_excl_AIH26, df_excl_AIH27, df_excl_AIH28, df_excl_AIH29, df_excl_AIH30, df_excl_AIH31, df_excl_AIH32, df_excl_AIH33, df_excl_AIH34, df_excl_WDHH1, df_excl_WDHH2, df_excl_WDHH3, df_excl_WDHH4, df_excl_WDHH5, df_excl_WDHH6, df_excl_WDHH7, df_excl_WDHH8, df_excl_WDHH9, df_excl_WDHH10, df_excl_WDHH11, df_excl_WDHH12, df_excl_WDHH13, df_excl_WDHH14, df_excl_WDHH15, df_excl_WDHH16, df_excl_WDHH17, df_excl_WDHH18, df_excl_WDHH19, df_excl_WDHH20, df_excl_WDHH21, df_excl_WDHH22, df_excl_WDHH23, df_excl_WDHH24, df_excl_WDHH25, df_excl_WDHH26, df_excl_WDHH27, df_excl_WDHH28, df_excl_WDHH29, df_excl_WDHH30, df_excl_WDHH31, df_excl_WDHH32, df_excl_WDHH33, df_excl_WDHH34, df_excl_Viral1, df_excl_Viral2, df_excl_Viral3, df_excl_Viral4, df_excl_Viral5, df_excl_Viral6, df_excl_Viral7, df_excl_Viral8, df_excl_Viral9, df_excl_Viral10, df_excl_Viral11, df_excl_Viral12, df_excl_Viral13, df_excl_Viral14, df_excl_Viral15, df_excl_Viral16, df_excl_Viral17, df_excl_Viral18, df_excl_Viral19, df_excl_Viral20, df_excl_Viral21, df_excl_Viral22, df_excl_Viral23, df_excl_Viral24, df_excl_Viral25, df_excl_Viral26, df_excl_Viral27, df_excl_Viral28, df_excl_Viral29, df_excl_Viral30, df_excl_Viral31, df_excl_Viral32, df_excl_Viral33, df_excl_Viral34)

df_clean <- anti_join(df, df_allexcld, by = "databaseID")

df_clean$Crytogenic_Cirrhosis <- as.factor(df_clean$Crytogenic_Cirrhosis)

df_clean$NAFLD <- as.factor(df_clean$NAFLD)

df_clean$NASH <- as.factor(df_clean$NASH)

df_clean$NAFL <- df_clean$NAFLD

df_clean$NAFLD <- ifelse(df_clean$NAFL =="0", ifelse(df_clean$NASH == "1", "1", "0"), "1")

df_clean$NAFLD <- as.factor(df_clean$NAFLD)

df_clean$NASH <- as.factor(df_clean$NASH)

df_clean$NAFL <- as.factor(df_clean$NAFL)

df_clean$Crytogenic_Cirrhosis <- as.factor(df_clean$Crytogenic_Cirrhosis)

df_clean %>% group_by(NAFL) %>% summarise(no_rows = length(NAFL))

df_clean %>% group_by(NASH) %>% summarise(no_rows = length(NASH))

df_clean %>% group_by(NAFLD) %>% summarise(no_rows = length(NAFLD))

df_clean %>% group_by(Crytogenic_Cirrhosis) %>% summarise(no_rows = length(Crytogenic_Cirrhosis))

df_clean$group1 <- as.factor(ifelse(df_clean$NAFLD =="0", ifelse(df_clean$Crytogenic_Cirrhosis == "0", "Control", "Cirrhosis"), "NAFLD"))

df_clean %>% group_by(group1) %>% summarise(no_rows = length(group1))

df_clean$group2 <- as.factor(ifelse(df_clean$NAFL =="0", ifelse(df_clean$Crytogenic_Cirrhosis == "0", ifelse(df_clean$NASH == "0", "Control", "NASH"), "Cirrhosis"), "NAFL"))

df_clean %>% group_by(group2) %>% summarise(no_rows = length(group2))

df_clean$NAFLDvsCtrl <- as.factor(ifelse(df_clean$group1 =="NAFLD", "NAFLD", ifelse(df_clean$group1 == "Control", "Control", "NA")))

df_clean$NAFLDvsCtrl <- na_if(df_clean$NAFLDvsCtrl, "NA")

df_clean %>% group_by(NAFLDvsCtrl) %>% summarise(no_rows = length(NAFLDvsCtrl))

df_clean$NAFLDvsCtrl <- droplevels(df_clean$NAFLDvsCtrl)

df_clean$NAFLDvsCtrl <- factor(df_clean$NAFLDvsCtrl, levels = c("Control", "NAFLD"))

df_clean$CirrvsCtrl <- as.factor(ifelse(df_clean$group1 =="Cirrhosis", "Cirrhosis", ifelse(df_clean$group1 == "Control", "Control", "NA")))

df_clean$CirrvsCtrl <- na_if(df_clean$CirrvsCtrl, "NA")

df_clean$CirrvsCtrl <- droplevels(df_clean$CirrvsCtrl)

df_clean %>% group_by(CirrvsCtrl) %>% summarise(no_rows = length(CirrvsCtrl))

df_clean$CirrvsCtrl <- factor(df_clean$CirrvsCtrl, levels = c("Control", "Cirrhosis"))

df_clean$CirrvsCtrl_num <- factor(df_clean$CirrvsCtrl, levels = c("1", "2"))

df_clean$CirrvsNAFLD <- as.factor(ifelse(df_clean$group1 =="NAFLD", "NAFLD", ifelse(df_clean$group1 == "Cirrhosis", "Cirrhosis", "NA")))

df_clean$CirrvsNAFLD <- na_if(df_clean$CirrvsNAFLD, "NA")

df_clean$CirrvsNAFLD <- droplevels(df_clean$CirrvsNAFLD)

df_clean %>% group_by(CirrvsNAFLD) %>% summarise(no_rows = length(CirrvsNAFLD))

df_clean$CirrvsNAFLD <- factor(df_clean$CirrvsNAFLD, levels = c("NAFLD", "Cirrhosis"))

df_clean$NAFLvsCtrl <- as.factor(ifelse(df_clean$group2 =="NAFL", "NAFL", ifelse(df_clean$group2 == "Control", "Control", "NA")))

df_clean$NAFLvsCtrl <- na_if(df_clean$NAFLvsCtrl, "NA")

df_clean$NAFLvsCtrl <- droplevels(df_clean$NAFLvsCtrl)

df_clean$NAFLvsCtrl <- factor(df_clean$NAFLvsCtrl, levels = c("Control", "NAFL"))

df_clean$NASHvsCtrl <- as.factor(ifelse(df_clean$group2 =="NASH", "NASH", ifelse(df_clean$group2 == "Control", "Control", "NA")))

df_clean$NASHvsCtrl <- na_if(df_clean$NASHvsCtrl, "NA")

df_clean$NASHvsCtrl <- droplevels(df_clean$NASHvsCtrl)

df_clean$NASHvsCtrl <- factor(df_clean$NASHvsCtrl, levels = c("Control", "NASH"))

df_clean$CirrvsNASH <- as.factor(ifelse(df_clean$group2 =="NASH", "NASH", ifelse(df_clean$group2 == "Cirrhosis", "Cirrhosis", "NA")))

df_clean$CirrvsNASH <- na_if(df_clean$CirrvsNASH, "NA")

df_clean$CirrvsNASH <- droplevels(df_clean$CirrvsNASH)

df_clean$CirrvsNASH <- factor(df_clean$CirrvsNASH, levels = c("NASH", "Cirrhosis"))

df_clean$CirrvsNAFL <- as.factor(ifelse(df_clean$group2 =="NAFL", "NAFL", ifelse(df_clean$group2 == "Cirrhosis", "Cirrhosis", "NA")))

df_clean$CirrvsNAFL <- na_if(df_clean$CirrvsNAFL, "NA")

df_clean$CirrvsNAFL <- droplevels(df_clean$CirrvsNAFL)

df_clean$CirrvsNAFL <- factor(df_clean$CirrvsNAFL, levels = c("NAFL", "Cirrhosis"))

df_clean$NAFLvsNASH <- as.factor(ifelse(df_clean$group2 =="NASH", "NASH", ifelse(df_clean$group2 == "NAFL", "NAFL", "NA")))

df_clean$NAFLvsNASH <- na_if(df_clean$NAFLvsNASH, "NA")

df_clean$NAFLvsNASH <- droplevels(df_clean$NAFLvsNASH)

df_clean$NAFLvsNASH <- factor(df_clean$NAFLvsNASH, levels = c("NAFL", "NASH"))

df_clean %>% group_by(ethnicf) %>% summarise(no_rows = length(ethnicf))

df_clean$ethnicf <- as.factor(df_clean$ethnicf)

df_clean$ethinc_group <- as.factor(ifelse(df_clean$ethnicf =="1", "White", ifelse(df_clean$ethnicf == "2", "Asian", "Other")))

df_clean %>% group_by(ethinc_group) %>% summarise(no_rows = length(ethinc_group))

df_clean$liver_decomp = df_clean$varicealbleed1 + df_clean$varices1 + df_clean$ascites1 + df_clean$hepatic_fibrosis1 + df_clean$portal_htn1 + df_clean$splenomegaly1 + df_clean$hepatorenal1

df_clean$liver_decomp_cat = ifelse(df_clean$liver_decomp > 0, "1", "0")

df_clean$any_cancer_temp1 = as.numeric(ifelse(str_detect(df_clean$d1, '(C0)|(C1)|(C2)|(C3)|(C4)|(C5)|(C6)|(C7)|(C8)|(C9)'), '1', '0'))

df_clean$any_cancer_temp2 = as.numeric(ifelse(str_detect(df_clean$d2, '(C0)|(C1)|(C2)|(C3)|(C4)|(C5)|(C6)|(C7)|(C8)|(C9)'), '1', '0'))

df_clean$any_cancer_temp3 = as.numeric(ifelse(str_detect(df_clean$d3, '(C0)|(C1)|(C2)|(C3)|(C4)|(C5)|(C6)|(C7)|(C8)|(C9)'), '1', '0'))

df_clean$any_cancer_temp4 = as.numeric(ifelse(str_detect(df_clean$d4, '(C0)|(C1)|(C2)|(C3)|(C4)|(C5)|(C6)|(C7)|(C8)|(C9)'), '1', '0'))

df_clean$any_cancer_temp5 = as.numeric(ifelse(str_detect(df_clean$d5, '(C0)|(C1)|(C2)|(C3)|(C4)|(C5)|(C6)|(C7)|(C8)|(C9)'), '1', '0'))

df_clean$any_cancer_temp6 = as.numeric(ifelse(str_detect(df_clean$d6, '(C0)|(C1)|(C2)|(C3)|(C4)|(C5)|(C6)|(C7)|(C8)|(C9)'), '1', '0'))

df_clean$any_cancer_temp7 = as.numeric(ifelse(str_detect(df_clean$d7, '(C0)|(C1)|(C2)|(C3)|(C4)|(C5)|(C6)|(C7)|(C8)|(C9)'), '1', '0'))

df_clean$any_cancer_temp8 = as.numeric(ifelse(str_detect(df_clean$d8, '(C0)|(C1)|(C2)|(C3)|(C4)|(C5)|(C6)|(C7)|(C8)|(C9)'), '1', '0'))

df_clean$any_cancer_temp9 = as.numeric(ifelse(str_detect(df_clean$d9, '(C0)|(C1)|(C2)|(C3)|(C4)|(C5)|(C6)|(C7)|(C8)|(C9)'), '1', '0'))

df_clean$any_cancer_temp10 = as.numeric(ifelse(str_detect(df_clean$d10, '(C0)|(C1)|(C2)|(C3)|(C4)|(C5)|(C6)|(C7)|(C8)|(C9)'), '1', '0'))

df_clean$any_cancer_temp11 = as.numeric(ifelse(str_detect(df_clean$d11, '(C0)|(C1)|(C2)|(C3)|(C4)|(C5)|(C6)|(C7)|(C8)|(C9)'), '1', '0'))

df_clean$any_cancer_temp12 = as.numeric(ifelse(str_detect(df_clean$d12, '(C0)|(C1)|(C2)|(C3)|(C4)|(C5)|(C6)|(C7)|(C8)|(C9)'), '1', '0'))

df_clean$any_cancer_temp13 = as.numeric(ifelse(str_detect(df_clean$d13, '(C0)|(C1)|(C2)|(C3)|(C4)|(C5)|(C6)|(C7)|(C8)|(C9)'), '1', '0'))

df_clean$any_cancer_temp14 = as.numeric(ifelse(str_detect(df_clean$d14, '(C0)|(C1)|(C2)|(C3)|(C4)|(C5)|(C6)|(C7)|(C8)|(C9)'), '1', '0'))

df_clean$any_cancer_temp15 = as.numeric(ifelse(str_detect(df_clean$d15, '(C0)|(C1)|(C2)|(C3)|(C4)|(C5)|(C6)|(C7)|(C8)|(C9)'), '1', '0'))

df_clean$any_cancer_temp16 = as.numeric(ifelse(str_detect(df_clean$d16, '(C0)|(C1)|(C2)|(C3)|(C4)|(C5)|(C6)|(C7)|(C8)|(C9)'), '1', '0'))

df_clean$any_cancer_temp17 = as.numeric(ifelse(str_detect(df_clean$d17, '(C0)|(C1)|(C2)|(C3)|(C4)|(C5)|(C6)|(C7)|(C8)|(C9)'), '1', '0'))

df_clean$any_cancer_temp18 = as.numeric(ifelse(str_detect(df_clean$d18, '(C0)|(C1)|(C2)|(C3)|(C4)|(C5)|(C6)|(C7)|(C8)|(C9)'), '1', '0'))

df_clean$any_cancer_temp19 = as.numeric(ifelse(str_detect(df_clean$d19, '(C0)|(C1)|(C2)|(C3)|(C4)|(C5)|(C6)|(C7)|(C8)|(C9)'), '1', '0'))

df_clean$any_cancer_temp20 = as.numeric(ifelse(str_detect(df_clean$d20, '(C0)|(C1)|(C2)|(C3)|(C4)|(C5)|(C6)|(C7)|(C8)|(C9)'), '1', '0'))

df_clean$any_cancer_temp21 = as.numeric(ifelse(str_detect(df_clean$d21, '(C0)|(C1)|(C2)|(C3)|(C4)|(C5)|(C6)|(C7)|(C8)|(C9)'), '1', '0'))

df_clean$any_cancer_temp22 = as.numeric(ifelse(str_detect(df_clean$d22, '(C0)|(C1)|(C2)|(C3)|(C4)|(C5)|(C6)|(C7)|(C8)|(C9)'), '1', '0'))

df_clean$any_cancer_temp23 = as.numeric(ifelse(str_detect(df_clean$d23, '(C0)|(C1)|(C2)|(C3)|(C4)|(C5)|(C6)|(C7)|(C8)|(C9)'), '1', '0'))

df_clean$any_cancer_temp24 = as.numeric(ifelse(str_detect(df_clean$d24, '(C0)|(C1)|(C2)|(C3)|(C4)|(C5)|(C6)|(C7)|(C8)|(C9)'), '1', '0'))

df_clean$any_cancer_temp25 = as.numeric(ifelse(str_detect(df_clean$d25, '(C0)|(C1)|(C2)|(C3)|(C4)|(C5)|(C6)|(C7)|(C8)|(C9)'), '1', '0'))

df_clean$any_cancer_temp26 = as.numeric(ifelse(str_detect(df_clean$d26, '(C0)|(C1)|(C2)|(C3)|(C4)|(C5)|(C6)|(C7)|(C8)|(C9)'), '1', '0'))

df_clean$any_cancer_temp27 = as.numeric(ifelse(str_detect(df_clean$d27, '(C0)|(C1)|(C2)|(C3)|(C4)|(C5)|(C6)|(C7)|(C8)|(C9)'), '1', '0'))

df_clean$any_cancer_temp28 = as.numeric(ifelse(str_detect(df_clean$d28, '(C0)|(C1)|(C2)|(C3)|(C4)|(C5)|(C6)|(C7)|(C8)|(C9)'), '1', '0'))

df_clean$any_cancer_temp29 = as.numeric(ifelse(str_detect(df_clean$d29, '(C0)|(C1)|(C2)|(C3)|(C4)|(C5)|(C6)|(C7)|(C8)|(C9)'), '1', '0'))

df_clean$any_cancer_temp30 = as.numeric(ifelse(str_detect(df_clean$d30, '(C0)|(C1)|(C2)|(C3)|(C4)|(C5)|(C6)|(C7)|(C8)|(C9)'), '1', '0'))

df_clean$any_cancer_temp31 = as.numeric(ifelse(str_detect(df_clean$d31, '(C0)|(C1)|(C2)|(C3)|(C4)|(C5)|(C6)|(C7)|(C8)|(C9)'), '1', '0'))

df_clean$any_cancer_temp32 = as.numeric(ifelse(str_detect(df_clean$d32, '(C0)|(C1)|(C2)|(C3)|(C4)|(C5)|(C6)|(C7)|(C8)|(C9)'), '1', '0'))

df_clean$any_cancer_temp33 = as.numeric(ifelse(str_detect(df_clean$d33, '(C0)|(C1)|(C2)|(C3)|(C4)|(C5)|(C6)|(C7)|(C8)|(C9)'), '1', '0'))

df_clean$any_cancer_temp34 = as.numeric(ifelse(str_detect(df_clean$d34, '(C0)|(C1)|(C2)|(C3)|(C4)|(C5)|(C6)|(C7)|(C8)|(C9)'), '1', '0'))

df_clean$all_cancer_temp <- rowSums(df_clean[,c("any_cancer_temp1", "any_cancer_temp2", "any_cancer_temp3", "any_cancer_temp4", "any_cancer_temp5", "any_cancer_temp6", "any_cancer_temp7", "any_cancer_temp8", "any_cancer_temp9", "any_cancer_temp10", "any_cancer_temp11", "any_cancer_temp12", "any_cancer_temp13", "any_cancer_temp14", "any_cancer_temp15", "any_cancer_temp16", "any_cancer_temp17", "any_cancer_temp18", "any_cancer_temp19", "any_cancer_temp20", "any_cancer_temp21", "any_cancer_temp22", "any_cancer_temp23", "any_cancer_temp24", "any_cancer_temp25", "any_cancer_temp26", "any_cancer_temp27", "any_cancer_temp28", "any_cancer_temp29", "any_cancer_temp30", "any_cancer_temp31", "any_cancer_temp32", "any_cancer_temp33", "any_cancer_temp34")], na.rm=TRUE)

df_clean$all_cancer = ifelse(df_clean$all_cancer_temp > 0, "1", "0")

df_clean %>% group_by(all_cancer) %>% summarise(no_rows = length(all_cancer))

df_clean$GI_cancer = df_clean$Anorectalca + df_clean$Colonca + df_clean$Livercancer + df_clean$Oesophagealcancer + df_clean$pancreasca + df_clean$smallintca + df_clean$stomachca

df_clean$GI_cancer = ifelse(df_clean$GI_cancer > 0, "1", "0")

df_clean$nonGI_cancer = df_clean$Breastca + df_clean$Cervical_cancer_all + df_clean$cervicalca + df_clean$Lungca + df_clean$Prostateca + df_clean$AcuteLL + df_clean$CLL + df_clean$CML + df_clean$AML + df_clean$malignmelanoma + df_clean$Hodgkins

df_clean$nonGI_cancer = ifelse(df_clean$nonGI_cancer > 0, "1", "0")

df_clean %>% group_by(deathind) %>% summarise(no_rows = length(deathind))

df_clean$gender <- as.factor(df_clean$gender)

df_clean$female = ifelse(df_clean$gender=="F", "1", "0")

df_clean$White_count = ifelse(df_clean$ethinc_group=="White", "1", "0")

df_clean$Asian_count = ifelse(df_clean$ethinc_group=="Asian", "1", "0")

df_clean$female <- as.factor(df_clean$female)

df_clean$deathind <- as.factor(df_clean$deathind)

df_clean$deathind <- ifelse(df_clean$deathind == "0", "1", "2")

df_clean$deathind <- as.numeric(df_clean$deathind)

df_clean$sex <- factor(df_clean$gender, levels = c("1", "2"), labels = c("F", "M"))

df_clean$sex <- as.numeric(df_clean$sex)

################

## descriptive stats table for main analysis (group1)

group1_sumtab <- df_clean %>% group_by(group1) %>% summarise(age_mean = mean(age), age_sd = sd(age), gender = sum(gender=="F"), White = sum(ethinc_group=="White"), South_Asian = sum(ethinc_group=="Asian"), total = n(), Obesity = sum(Obesity=='1'), T2DM = sum(T2DM=='1'), HYPERLIPIDAEMIA = sum(HYPERLIPIDAEMIA=='1'), HEARTFAILURE = sum(HEARTFAILURE=='1'), AF = sum(AF=='1'), CKD = sum(CKD=='1'), IHD = sum(IHD=='1'), MI = sum(MI=='1'), carotidarterydisease = sum(carotidarterydisease=='1'), ISCHAEMICSTROKE = sum(ISCHAEMICSTROKE=='1'), hyperten = sum(hyperten=='1'), PVD = sum(PVD=='1'), Livercancer = sum(Livercancer=='1'), liver_decomp_cat = sum(liver_decomp_cat=='1'), GI_cancer = sum(GI_cancer=='1'), nonGI_cancer = sum(nonGI_cancer=='1'), all_cancer = sum(all_cancer=='1'), deathind = sum(deathind=='1'))

group1_sumtab$gender_per = (group1_sumtab$gender/group1_sumtab$total*100)

group1_sumtab$White_per = (group1_sumtab$White/group1_sumtab$total*100)

group1_sumtab$South_Asian_per = (group1_sumtab$South_Asian/group1_sumtab$total*100)

group1_sumtab$Obesity_per = (group1_sumtab$Obesity/group1_sumtab$total*100)

group1_sumtab$T2DM_per = (group1_sumtab$T2DM/group1_sumtab$total*100)

group1_sumtab$HYPERLIPIDAEMIA_per = (group1_sumtab$HYPERLIPIDAEMIA/group1_sumtab$total*100)

group1_sumtab$HEARTFAILURE_per = (group1_sumtab$HEARTFAILURE/group1_sumtab$total*100)

group1_sumtab$AF_per = (group1_sumtab$AF/group1_sumtab$total*100)

group1_sumtab$CKD_per = (group1_sumtab$CKD/group1_sumtab$total*100)

group1_sumtab$IHD_per = (group1_sumtab$IHD/group1_sumtab$total*100)

group1_sumtab$MI_per = (group1_sumtab$MI/group1_sumtab$total*100)

group1_sumtab$carotidarterydisease_per = (group1_sumtab$carotidarterydisease/group1_sumtab$total*100)

group1_sumtab$ISCHAEMICSTROKE_per = (group1_sumtab$ISCHAEMICSTROKE/group1_sumtab$total*100)

group1_sumtab$hyperten_per = (group1_sumtab$hyperten/group1_sumtab$total*100)

group1_sumtab$PVD_per = (group1_sumtab$PVD/group1_sumtab$total*100)

group1_sumtab$Livercancer_per = (group1_sumtab$Livercancer/group1_sumtab$total*100)

group1_sumtab$liver_decomp_cat_per = (group1_sumtab$liver_decomp_cat/group1_sumtab$total*100)

group1_sumtab$GI_cancer_per = (group1_sumtab$GI_cancer/group1_sumtab$total*100)

group1_sumtab$nonGI_cancer_per = (group1_sumtab$nonGI_cancer/group1_sumtab$total*100)

group1_sumtab$all_cancer_per = (group1_sumtab$all_cancer/group1_sumtab$total*100)

group1_sumtab$deathind_per = (group1_sumtab$deathind/group1_sumtab$total*100)

group1_sumtab$age_mean_text <- paste(format(round(group1_sumtab$age_mean, 1), nsmall = 1), format(round(group1_sumtab$age_sd, 1), nsmall = 1), sep = ' (', collapse = NULL)

group1_sumtab$gender_text <- paste(group1_sumtab$gender, format(round(group1_sumtab$gender_per, 1), nsmall = 1), sep = ' (', collapse = NULL)

group1_sumtab$White_text <- paste(group1_sumtab$White, format(round(group1_sumtab$White_per, 1), nsmall = 1), sep = ' (', collapse = NULL)

group1_sumtab$South_Asian_text <- paste(group1_sumtab$South_Asian, format(round(group1_sumtab$South_Asian_per, 1), nsmall = 1), sep = ' (', collapse = NULL)

group1_sumtab$Obesity_text <- paste(group1_sumtab$Obesity, format(round(group1_sumtab$Obesity_per, 1), nsmall = 1), sep = ' (', collapse = NULL)

group1_sumtab$T2DM_text <- paste(group1_sumtab$T2DM, format(round(group1_sumtab$T2DM_per, 1), nsmall = 1), sep = ' (', collapse = NULL)

group1_sumtab$HYPERLIPIDAEMIA_text <- paste(group1_sumtab$HYPERLIPIDAEMIA, format(round(group1_sumtab$HYPERLIPIDAEMIA_per, 1), nsmall = 1), sep = ' (', collapse = NULL)

group1_sumtab$HEARTFAILURE_text <- paste(group1_sumtab$HEARTFAILURE, format(round(group1_sumtab$HEARTFAILURE_per, 1), nsmall = 1), sep = ' (', collapse = NULL)

group1_sumtab$AF_text <- paste(group1_sumtab$AF, format(round(group1_sumtab$AF_per, 1), nsmall = 1), sep = ' (', collapse = NULL)

group1_sumtab$CKD_text <- paste(group1_sumtab$CKD, format(round(group1_sumtab$CKD_per, 1), nsmall = 1), sep = ' (', collapse = NULL)

group1_sumtab$IHD_text <- paste(group1_sumtab$IHD, format(round(group1_sumtab$IHD_per, 1), nsmall = 1), sep = ' (', collapse = NULL)

group1_sumtab$MI_text <- paste(group1_sumtab$MI, format(round(group1_sumtab$MI_per, 1), nsmall = 1), sep = ' (', collapse = NULL)

group1_sumtab$carotidarterydisease_text <- paste(group1_sumtab$carotidarterydisease, format(round(group1_sumtab$carotidarterydisease_per, 1), nsmall = 1), sep = ' (', collapse = NULL)

group1_sumtab$ISCHAEMICSTROKE_text <- paste(group1_sumtab$ISCHAEMICSTROKE, format(round(group1_sumtab$ISCHAEMICSTROKE_per, 1), nsmall = 1), sep = ' (', collapse = NULL)

group1_sumtab$hyperten_text <- paste(group1_sumtab$hyperten, format(round(group1_sumtab$hyperten_per, 1), nsmall = 1), sep = ' (', collapse = NULL)

group1_sumtab$PVD_text <- paste(group1_sumtab$PVD, format(round(group1_sumtab$PVD_per, 1), nsmall = 1), sep = ' (', collapse = NULL)

group1_sumtab$Livercancer_text <- paste(group1_sumtab$Livercancer, format(round(group1_sumtab$Livercancer_per, 1), nsmall = 1), sep = ' (', collapse = NULL)

group1_sumtab$liver_decomp_cat_text <- paste(group1_sumtab$liver_decomp_cat, format(round(group1_sumtab$liver_decomp_cat_per, 1), nsmall = 1), sep = ' (', collapse = NULL)

group1_sumtab$GI_cancer_text <- paste(group1_sumtab$GI_cancer, format(round(group1_sumtab$GI_cancer_per, 1), nsmall = 1), sep = ' (', collapse = NULL)

group1_sumtab$nonGI_cancer_text <- paste(group1_sumtab$nonGI_cancer, format(round(group1_sumtab$nonGI_cancer_per, 1), nsmall = 1), sep = ' (', collapse = NULL)

group1_sumtab$all_cancer_text <- paste(group1_sumtab$all_cancer, format(round(group1_sumtab$all_cancer_per, 1), nsmall = 1), sep = ' (', collapse = NULL)

group1_sumtab$deathind_text <- paste(group1_sumtab$deathind, format(round(group1_sumtab$deathind_per, 1), nsmall = 1), sep = ' (', collapse = NULL)

group1_sumtab$age_mean <- paste(group1_sumtab$age_mean_text, '', sep = ')', collapse = NULL)

group1_sumtab$gender <- paste(group1_sumtab$gender_text, '', sep = ')', collapse = NULL)

group1_sumtab$White <- paste(group1_sumtab$White_text, '', sep = ')', collapse = NULL)

group1_sumtab$South_Asian <- paste(group1_sumtab$South_Asian_text, '', sep = ')', collapse = NULL)

group1_sumtab$Obesity <- paste(group1_sumtab$Obesity_text, '', sep = ')', collapse = NULL)

group1_sumtab$T2DM <- paste(group1_sumtab$T2DM_text, '', sep = ')', collapse = NULL)

group1_sumtab$HYPERLIPIDAEMIA <- paste(group1_sumtab$HYPERLIPIDAEMIA_text, '', sep = ')', collapse = NULL)

group1_sumtab$HEARTFAILURE <- paste(group1_sumtab$HEARTFAILURE_text, '', sep = ')', collapse = NULL)

group1_sumtab$AF <- paste(group1_sumtab$AF_text, '', sep = ')', collapse = NULL)

group1_sumtab$CKD <- paste(group1_sumtab$CKD_text, '', sep = ')', collapse = NULL)

group1_sumtab$IHD <- paste(group1_sumtab$IHD_text, '', sep = ')', collapse = NULL)

group1_sumtab$MI <- paste(group1_sumtab$MI_text, '', sep = ')', collapse = NULL)

group1_sumtab$carotidarterydisease <- paste(group1_sumtab$carotidarterydisease_text, '', sep = ')', collapse = NULL)

group1_sumtab$ISCHAEMICSTROKE <- paste(group1_sumtab$ISCHAEMICSTROKE_text, '', sep = ')', collapse = NULL)

group1_sumtab$hyperten <- paste(group1_sumtab$hyperten_text, '', sep = ')', collapse = NULL)

group1_sumtab$PVD <- paste(group1_sumtab$PVD_text, '', sep = ')', collapse = NULL)

group1_sumtab$Livercancer <- paste(group1_sumtab$Livercancer_text, '', sep = ')', collapse = NULL)

group1_sumtab$liver_decomp_cat <- paste(group1_sumtab$liver_decomp_cat_text, '', sep = ')', collapse = NULL)

group1_sumtab$GI_cancer <- paste(group1_sumtab$GI_cancer_text, '', sep = ')', collapse = NULL)

group1_sumtab$nonGI_cancer <- paste(group1_sumtab$nonGI_cancer_text, '', sep = ')', collapse = NULL)

group1_sumtab$all_cancer <- paste(group1_sumtab$all_cancer_text, '', sep = ')', collapse = NULL)

group1_sumtab$deathind <- paste(group1_sumtab$deathind_text, '', sep = ')', collapse = NULL)

rownames(group1_sumtab) = group1_sumtab$group1

group1_sumtab_t <- as.data.frame(t(group1_sumtab))

group1_sumtab_t <- group1_sumtab_t[-c(1),]

group1_sumtab_t$var <- rownames(group1_sumtab_t)

### Run chi-squared tests

NAFLDvsCtrl_age <- t.test(age ~ NAFLDvsCtrl, data = df_clean)

NAFLDvsCtrl_female <- chisq.test(x = table(df_clean$NAFLDvsCtrl, df_clean$female))

NAFLDvsCtrl_White_count <- chisq.test(x = table(df_clean$NAFLDvsCtrl, df_clean$White_count))

NAFLDvsCtrl_Asian_count <- chisq.test(x = table(df_clean$NAFLDvsCtrl, df_clean$Asian_count))

NAFLDvsCtrl_Obesity <- chisq.test(x = table(df_clean$NAFLDvsCtrl, df_clean$Obesity))

NAFLDvsCtrl_T2DM <- chisq.test(x = table(df_clean$NAFLDvsCtrl, df_clean$T2DM))

NAFLDvsCtrl_HYPERLIPIDAEMIA <- chisq.test(x = table(df_clean$NAFLDvsCtrl, df_clean$HYPERLIPIDAEMIA))

NAFLDvsCtrl_HEARTFAILURE <- chisq.test(x = table(df_clean$NAFLDvsCtrl, df_clean$HEARTFAILURE))

NAFLDvsCtrl_AF <- chisq.test(x = table(df_clean$NAFLDvsCtrl, df_clean$AF))

NAFLDvsCtrl_CKD <- chisq.test(x = table(df_clean$NAFLDvsCtrl, df_clean$CKD))

NAFLDvsCtrl_IHD <- chisq.test(x = table(df_clean$NAFLDvsCtrl, df_clean$IHD))

NAFLDvsCtrl_MI <- chisq.test(x = table(df_clean$NAFLDvsCtrl, df_clean$MI))

NAFLDvsCtrl_carotidarterydisease <- chisq.test(x = table(df_clean$NAFLDvsCtrl, df_clean$carotidarterydisease))

NAFLDvsCtrl_ISCHAEMICSTROKE <- chisq.test(x = table(df_clean$NAFLDvsCtrl, df_clean$ISCHAEMICSTROKE))

NAFLDvsCtrl_hyperten <- chisq.test(x = table(df_clean$NAFLDvsCtrl, df_clean$hyperten))

NAFLDvsCtrl_PVD <- chisq.test(x = table(df_clean$NAFLDvsCtrl, df_clean$PVD))

NAFLDvsCtrl_Livercancer <- chisq.test(x = table(df_clean$NAFLDvsCtrl, df_clean$Livercancer))

NAFLDvsCtrl_liver_decomp_cat <- chisq.test(x = table(df_clean$NAFLDvsCtrl, df_clean$liver_decomp_cat))

NAFLDvsCtrl_GI_cancer <- chisq.test(x = table(df_clean$NAFLDvsCtrl, df_clean$GI_cancer))

NAFLDvsCtrl_nonGI_cancer <- chisq.test(x = table(df_clean$NAFLDvsCtrl, df_clean$nonGI_cancer))

NAFLDvsCtrl_all_cancer <- chisq.test(x = table(df_clean$NAFLDvsCtrl, df_clean$all_cancer))

NAFLDvsCtrl_deathind <- chisq.test(x = table(df_clean$NAFLDvsCtrl, df_clean$deathind))

NAFLDvsCtrl_pval <- data.frame(c(NAFLDvsCtrl_age$p.value, NA, NAFLDvsCtrl_female$p.value, NAFLDvsCtrl_White_count$p.value, NAFLDvsCtrl_Asian_count$p.value, "1", NAFLDvsCtrl_Obesity$p.value, NAFLDvsCtrl_T2DM$p.value, NAFLDvsCtrl_HYPERLIPIDAEMIA$p.value, NAFLDvsCtrl_HEARTFAILURE$p.value, NAFLDvsCtrl_AF$p.value, NAFLDvsCtrl_CKD$p.value, NAFLDvsCtrl_IHD$p.value, NAFLDvsCtrl_MI$p.value, NAFLDvsCtrl_carotidarterydisease$p.value, NAFLDvsCtrl_ISCHAEMICSTROKE$p.value, NAFLDvsCtrl_hyperten$p.value, NAFLDvsCtrl_PVD$p.value, NAFLDvsCtrl_Livercancer$p.value, NAFLDvsCtrl_liver_decomp_cat$p.value, NAFLDvsCtrl_GI_cancer$p.value, NAFLDvsCtrl_nonGI_cancer$p.value, NAFLDvsCtrl_all_cancer$p.value, NAFLDvsCtrl_deathind$p.value))

NAFLDvsCtrl_pval <- NAFLDvsCtrl_pval %>% rename(NAFLDvsCtrl_pval = c(1))

NAFLDvsCtrl_pval$var <- c("age_mean","age_sd","gender","White","South_Asian","total","Obesity","T2DM","HYPERLIPIDAEMIA","HEARTFAILURE","AF","CKD","IHD","MI","carotidarterydisease","ISCHAEMICSTROKE","hyperten","PVD","Livercancer","liver_decomp_cat","GI_cancer","nonGI_cancer","all_cancer","deathind")

CirrvsCtrl_age <- t.test(age ~ CirrvsCtrl, data = df_clean)

CirrvsCtrl_female <- chisq.test(x = table(df_clean$CirrvsCtrl, df_clean$female))

CirrvsCtrl_White_count <- chisq.test(x = table(df_clean$CirrvsCtrl, df_clean$White_count))

CirrvsCtrl_Asian_count <- chisq.test(x = table(df_clean$CirrvsCtrl, df_clean$Asian_count))

CirrvsCtrl_Obesity <- chisq.test(x = table(df_clean$CirrvsCtrl, df_clean$Obesity))

CirrvsCtrl_T2DM <- chisq.test(x = table(df_clean$CirrvsCtrl, df_clean$T2DM))

CirrvsCtrl_HYPERLIPIDAEMIA <- chisq.test(x = table(df_clean$CirrvsCtrl, df_clean$HYPERLIPIDAEMIA))

CirrvsCtrl_HEARTFAILURE <- chisq.test(x = table(df_clean$CirrvsCtrl, df_clean$HEARTFAILURE))

CirrvsCtrl_AF <- chisq.test(x = table(df_clean$CirrvsCtrl, df_clean$AF))

CirrvsCtrl_CKD <- chisq.test(x = table(df_clean$CirrvsCtrl, df_clean$CKD))

CirrvsCtrl_IHD <- chisq.test(x = table(df_clean$CirrvsCtrl, df_clean$IHD))

CirrvsCtrl_MI <- chisq.test(x = table(df_clean$CirrvsCtrl, df_clean$MI))

CirrvsCtrl_carotidarterydisease <- chisq.test(x = table(df_clean$CirrvsCtrl, df_clean$carotidarterydisease))

CirrvsCtrl_ISCHAEMICSTROKE <- chisq.test(x = table(df_clean$CirrvsCtrl, df_clean$ISCHAEMICSTROKE))

CirrvsCtrl_hyperten <- chisq.test(x = table(df_clean$CirrvsCtrl, df_clean$hyperten))

CirrvsCtrl_PVD <- chisq.test(x = table(df_clean$CirrvsCtrl, df_clean$PVD))

CirrvsCtrl_Livercancer <- chisq.test(x = table(df_clean$CirrvsCtrl, df_clean$Livercancer))

CirrvsCtrl_liver_decomp_cat <- chisq.test(x = table(df_clean$CirrvsCtrl, df_clean$liver_decomp_cat))

CirrvsCtrl_GI_cancer <- chisq.test(x = table(df_clean$CirrvsCtrl, df_clean$GI_cancer))

CirrvsCtrl_nonGI_cancer <- chisq.test(x = table(df_clean$CirrvsCtrl, df_clean$nonGI_cancer))

CirrvsCtrl_all_cancer <- chisq.test(x = table(df_clean$CirrvsCtrl, df_clean$all_cancer))

CirrvsCtrl_deathind <- chisq.test(x = table(df_clean$CirrvsCtrl, df_clean$deathind))

CirrvsCtrl_pval <- data.frame(c(CirrvsCtrl_age$p.value, NA, CirrvsCtrl_female$p.value, CirrvsCtrl_White_count$p.value, CirrvsCtrl_Asian_count$p.value, "1", CirrvsCtrl_Obesity$p.value, CirrvsCtrl_T2DM$p.value, CirrvsCtrl_HYPERLIPIDAEMIA$p.value, CirrvsCtrl_HEARTFAILURE$p.value, CirrvsCtrl_AF$p.value, CirrvsCtrl_CKD$p.value, CirrvsCtrl_IHD$p.value, CirrvsCtrl_MI$p.value, CirrvsCtrl_carotidarterydisease$p.value, CirrvsCtrl_ISCHAEMICSTROKE$p.value, CirrvsCtrl_hyperten$p.value, CirrvsCtrl_PVD$p.value, CirrvsCtrl_Livercancer$p.value, CirrvsCtrl_liver_decomp_cat$p.value, CirrvsCtrl_GI_cancer$p.value, CirrvsCtrl_nonGI_cancer$p.value, CirrvsCtrl_all_cancer$p.value, CirrvsCtrl_deathind$p.value))

CirrvsCtrl_pval <- CirrvsCtrl_pval %>% rename(CirrvsCtrl_pval = c(1))

CirrvsCtrl_pval$var <- c("age_mean","age_sd","gender","White","South_Asian","total","Obesity","T2DM","HYPERLIPIDAEMIA","HEARTFAILURE","AF","CKD","IHD","MI","carotidarterydisease","ISCHAEMICSTROKE","hyperten","PVD","Livercancer","liver_decomp_cat","GI_cancer","nonGI_cancer","all_cancer","deathind")

CirrvsNAFLD_age <- t.test(age ~ CirrvsNAFLD, data = df_clean)

CirrvsNAFLD_female <- chisq.test(x = table(df_clean$CirrvsNAFLD, df_clean$female))

CirrvsNAFLD_White_count <- chisq.test(x = table(df_clean$CirrvsNAFLD, df_clean$White_count))

CirrvsNAFLD_Asian_count <- chisq.test(x = table(df_clean$CirrvsNAFLD, df_clean$Asian_count))

CirrvsNAFLD_Obesity <- chisq.test(x = table(df_clean$CirrvsNAFLD, df_clean$Obesity))

CirrvsNAFLD_T2DM <- chisq.test(x = table(df_clean$CirrvsNAFLD, df_clean$T2DM))

CirrvsNAFLD_HYPERLIPIDAEMIA <- chisq.test(x = table(df_clean$CirrvsNAFLD, df_clean$HYPERLIPIDAEMIA))

CirrvsNAFLD_HEARTFAILURE <- chisq.test(x = table(df_clean$CirrvsNAFLD, df_clean$HEARTFAILURE))

CirrvsNAFLD_AF <- chisq.test(x = table(df_clean$CirrvsNAFLD, df_clean$AF))

CirrvsNAFLD_CKD <- chisq.test(x = table(df_clean$CirrvsNAFLD, df_clean$CKD))

CirrvsNAFLD_IHD <- chisq.test(x = table(df_clean$CirrvsNAFLD, df_clean$IHD))

CirrvsNAFLD_MI <- chisq.test(x = table(df_clean$CirrvsNAFLD, df_clean$MI))

CirrvsNAFLD_carotidarterydisease <- chisq.test(x = table(df_clean$CirrvsNAFLD, df_clean$carotidarterydisease))

CirrvsNAFLD_ISCHAEMICSTROKE <- chisq.test(x = table(df_clean$CirrvsNAFLD, df_clean$ISCHAEMICSTROKE))

CirrvsNAFLD_hyperten <- chisq.test(x = table(df_clean$CirrvsNAFLD, df_clean$hyperten))

CirrvsNAFLD_PVD <- chisq.test(x = table(df_clean$CirrvsNAFLD, df_clean$PVD))

CirrvsNAFLD_Livercancer <- chisq.test(x = table(df_clean$CirrvsNAFLD, df_clean$Livercancer))

CirrvsNAFLD_liver_decomp_cat <- chisq.test(x = table(df_clean$CirrvsNAFLD, df_clean$liver_decomp_cat))

CirrvsNAFLD_GI_cancer <- chisq.test(x = table(df_clean$CirrvsNAFLD, df_clean$GI_cancer))

CirrvsNAFLD_nonGI_cancer <- chisq.test(x = table(df_clean$CirrvsNAFLD, df_clean$nonGI_cancer))

CirrvsNAFLD_all_cancer <- chisq.test(x = table(df_clean$CirrvsNAFLD, df_clean$all_cancer))

CirrvsNAFLD_deathind <- chisq.test(x = table(df_clean$CirrvsNAFLD, df_clean$deathind))

CirrvsNAFLD_pval <- data.frame(c(CirrvsNAFLD_age$p.value, NA, CirrvsNAFLD_female$p.value, CirrvsNAFLD_White_count$p.value, CirrvsNAFLD_Asian_count$p.value, "1", CirrvsNAFLD_Obesity$p.value, CirrvsNAFLD_T2DM$p.value, CirrvsNAFLD_HYPERLIPIDAEMIA$p.value, CirrvsNAFLD_HEARTFAILURE$p.value, CirrvsNAFLD_AF$p.value, CirrvsNAFLD_CKD$p.value, CirrvsNAFLD_IHD$p.value, CirrvsNAFLD_MI$p.value, CirrvsNAFLD_carotidarterydisease$p.value, CirrvsNAFLD_ISCHAEMICSTROKE$p.value, CirrvsNAFLD_hyperten$p.value, CirrvsNAFLD_PVD$p.value, CirrvsNAFLD_Livercancer$p.value, CirrvsNAFLD_liver_decomp_cat$p.value, CirrvsNAFLD_GI_cancer$p.value, CirrvsNAFLD_nonGI_cancer$p.value, CirrvsNAFLD_all_cancer$p.value, CirrvsNAFLD_deathind$p.value))

CirrvsNAFLD_pval <- CirrvsNAFLD_pval %>% rename(CirrvsNAFLD_pval = c(1))

CirrvsNAFLD_pval$var <- c("age_mean","age_sd","gender","White","South_Asian","total","Obesity","T2DM","HYPERLIPIDAEMIA","HEARTFAILURE","AF","CKD","IHD","MI","carotidarterydisease","ISCHAEMICSTROKE","hyperten","PVD","Livercancer","liver_decomp_cat","GI_cancer","nonGI_cancer","all_cancer","deathind")

group1_sumtab_v2 <- merge(group1_sumtab_t, NAFLDvsCtrl_pval, by="var", all=TRUE)

group1_sumtab_v2 <- merge(group1_sumtab_v2, CirrvsCtrl_pval, by="var", all=TRUE)

group1_sumtab_v2 <- merge(group1_sumtab_v2, CirrvsNAFLD_pval, by="var", all=TRUE)

group1_sumtab_v2 <- group1_sumtab_v2 %>% drop_na()

group1_sumtab_v2$NAFLDvsCtrl_qval <- p.adjust(group1_sumtab_v2$NAFLDvsCtrl_pval, method = "BH")

group1_sumtab_v2$CirrvsCtrl_qval <- p.adjust(group1_sumtab_v2$CirrvsCtrl_pval, method = "BH")

group1_sumtab_v2$CirrvsNAFLD_qval <- p.adjust(group1_sumtab_v2$CirrvsNAFLD_pval, method = "BH")

group1_sumtab_v2$NAFLDvsCtrl_qval <- ifelse(group1_sumtab_v2$NAFLDvsCtrl_qval <.01, format(group1_sumtab_v2$NAFLDvsCtrl_qval, scientific=T, digits=2), format(round(group1_sumtab_v2$NAFLDvsCtrl_qval, 2), nsmall = 2))

group1_sumtab_v2$CirrvsCtrl_qval <- ifelse(group1_sumtab_v2$CirrvsCtrl_qval <.01, format(group1_sumtab_v2$CirrvsCtrl_qval, scientific=T, digits=2), format(round(group1_sumtab_v2$CirrvsCtrl_qval, 2), nsmall = 2))

group1_sumtab_v2$CirrvsNAFLD_qval <- ifelse(group1_sumtab_v2$CirrvsNAFLD_qval <.01, format(group1_sumtab_v2$CirrvsNAFLD_qval, scientific=T, digits=2), format(round(group1_sumtab_v2$CirrvsNAFLD_qval, 2), nsmall = 2))

write.table(group1_sumtab_v2, file="group1_sumtab_v2.csv", sep=",")

############

## run adjusted log oddsratios for group1

NAFLDvsCtrl_liver_decomp_cat_OR <- glm(df_clean$NAFLDvsCtrl ~ df_clean$liver_decomp_cat + df_clean$age + df_clean$gender, family = binomial, data = df_clean)

NAFLDvsCtrl_liver_decomp_cat_OR_tab <- as.data.frame(confint(NAFLDvsCtrl_liver_decomp_cat_OR))

NAFLDvsCtrl_liver_decomp_cat_OR_tab$OR <- summary(NAFLDvsCtrl_liver_decomp_cat_OR)$coefficients[,1]

NAFLDvsCtrl_liver_decomp_cat_OR_tab$z_score <- summary(NAFLDvsCtrl_liver_decomp_cat_OR)$coefficients[,3]

NAFLDvsCtrl_liver_decomp_cat_OR_tab$NAFLDvsCtrl_pval <- 2*pnorm(-abs(NAFLDvsCtrl_liver_decomp_cat_OR_tab$z_score))

NAFLDvsCtrl_liver_decomp_cat_OR_tab <- NAFLDvsCtrl_liver_decomp_cat_OR_tab %>% rename(lower = c(1))

NAFLDvsCtrl_liver_decomp_cat_OR_tab <- NAFLDvsCtrl_liver_decomp_cat_OR_tab %>% rename(upper = c(2))

NAFLDvsCtrl_liver_decomp_cat_OR_tab$NAFLDvsCtrl_text <- paste(format(round(NAFLDvsCtrl_liver_decomp_cat_OR_tab$OR, 1), nsmall = 1), format(round(NAFLDvsCtrl_liver_decomp_cat_OR_tab$lower, 1), nsmall = 1), sep = ' (', collapse = NULL)

NAFLDvsCtrl_liver_decomp_cat_OR_tab$NAFLDvsCtrl_text <- paste(NAFLDvsCtrl_liver_decomp_cat_OR_tab$NAFLDvsCtrl_text, format(round(NAFLDvsCtrl_liver_decomp_cat_OR_tab$upper, 1), nsmall = 1), sep = '-', collapse = NULL)

NAFLDvsCtrl_liver_decomp_cat_OR_tab$NAFLDvsCtrl_text <- paste(NAFLDvsCtrl_liver_decomp_cat_OR_tab$NAFLDvsCtrl_text, '', sep = ')', collapse = NULL)

NAFLDvsCtrl_liver_decomp_cat_OR_tab$var <- "liver_decomp_cat"

NAFLDvsCtrl_liver_decomp_cat_OR_tab <- NAFLDvsCtrl_liver_decomp_cat_OR_tab %>% slice(2)

NAFLDvsCtrl_liver_decomp_cat_OR_tab <- NAFLDvsCtrl_liver_decomp_cat_OR_tab %>% select(5:7)

NAFLDvsCtrl_Livercancer_OR <- glm(df_clean$NAFLDvsCtrl ~ df_clean$Livercancer + df_clean$age + df_clean$gender, family = binomial, data = df_clean)

NAFLDvsCtrl_Livercancer_OR_tab <- as.data.frame(confint(NAFLDvsCtrl_Livercancer_OR))

NAFLDvsCtrl_Livercancer_OR_tab$OR <- summary(NAFLDvsCtrl_Livercancer_OR)$coefficients[,1]

NAFLDvsCtrl_Livercancer_OR_tab$z_score <- summary(NAFLDvsCtrl_Livercancer_OR)$coefficients[,3]

NAFLDvsCtrl_Livercancer_OR_tab$NAFLDvsCtrl_pval <- 2*pnorm(-abs(NAFLDvsCtrl_Livercancer_OR_tab$z_score))

NAFLDvsCtrl_Livercancer_OR_tab <- NAFLDvsCtrl_Livercancer_OR_tab %>% rename(lower = c(1))

NAFLDvsCtrl_Livercancer_OR_tab <- NAFLDvsCtrl_Livercancer_OR_tab %>% rename(upper = c(2))

NAFLDvsCtrl_Livercancer_OR_tab$NAFLDvsCtrl_text <- paste(format(round(NAFLDvsCtrl_Livercancer_OR_tab$OR, 1), nsmall = 1), format(round(NAFLDvsCtrl_Livercancer_OR_tab$lower, 1), nsmall = 1), sep = ' (', collapse = NULL)

NAFLDvsCtrl_Livercancer_OR_tab$NAFLDvsCtrl_text <- paste(NAFLDvsCtrl_Livercancer_OR_tab$NAFLDvsCtrl_text, format(round(NAFLDvsCtrl_Livercancer_OR_tab$upper, 1), nsmall = 1), sep = '-', collapse = NULL)

NAFLDvsCtrl_Livercancer_OR_tab$NAFLDvsCtrl_text <- paste(NAFLDvsCtrl_Livercancer_OR_tab$NAFLDvsCtrl_text, '', sep = ')', collapse = NULL)

NAFLDvsCtrl_Livercancer_OR_tab$var <- "Livercancer"

NAFLDvsCtrl_Livercancer_OR_tab <- NAFLDvsCtrl_Livercancer_OR_tab %>% slice(2)

NAFLDvsCtrl_Livercancer_OR_tab <- NAFLDvsCtrl_Livercancer_OR_tab %>% select(5:7)

CirrvsCtrl_liver_decomp_cat_OR <- glm(df_clean$CirrvsCtrl ~ df_clean$liver_decomp_cat + df_clean$age + df_clean$gender, family = binomial, data = df_clean)

CirrvsCtrl_liver_decomp_cat_OR_tab <- as.data.frame(confint(CirrvsCtrl_liver_decomp_cat_OR))

CirrvsCtrl_liver_decomp_cat_OR_tab$OR <- summary(CirrvsCtrl_liver_decomp_cat_OR)$coefficients[,1]

CirrvsCtrl_liver_decomp_cat_OR_tab$z_score <- summary(CirrvsCtrl_liver_decomp_cat_OR)$coefficients[,3]

CirrvsCtrl_liver_decomp_cat_OR_tab$CirrvsCtrl_pval <- 2*pnorm(-abs(CirrvsCtrl_liver_decomp_cat_OR_tab$z_score))

CirrvsCtrl_liver_decomp_cat_OR_tab <- CirrvsCtrl_liver_decomp_cat_OR_tab %>% rename(lower = c(1))

CirrvsCtrl_liver_decomp_cat_OR_tab <- CirrvsCtrl_liver_decomp_cat_OR_tab %>% rename(upper = c(2))

CirrvsCtrl_liver_decomp_cat_OR_tab$CirrvsCtrl_text <- paste(format(round(CirrvsCtrl_liver_decomp_cat_OR_tab$OR, 1), nsmall = 1), format(round(CirrvsCtrl_liver_decomp_cat_OR_tab$lower, 1), nsmall = 1), sep = ' (', collapse = NULL)

CirrvsCtrl_liver_decomp_cat_OR_tab$CirrvsCtrl_text <- paste(CirrvsCtrl_liver_decomp_cat_OR_tab$CirrvsCtrl_text, format(round(CirrvsCtrl_liver_decomp_cat_OR_tab$upper, 1), nsmall = 1), sep = '-', collapse = NULL)

CirrvsCtrl_liver_decomp_cat_OR_tab$CirrvsCtrl_text <- paste(CirrvsCtrl_liver_decomp_cat_OR_tab$CirrvsCtrl_text, '', sep = ')', collapse = NULL)

CirrvsCtrl_liver_decomp_cat_OR_tab$var <- "liver_decomp_cat"

CirrvsCtrl_liver_decomp_cat_OR_tab <- CirrvsCtrl_liver_decomp_cat_OR_tab %>% slice(2)

CirrvsCtrl_liver_decomp_cat_OR_tab <- CirrvsCtrl_liver_decomp_cat_OR_tab %>% select(5:7)

CirrvsCtrl_Livercancer_OR <- glm(df_clean$CirrvsCtrl ~ df_clean$Livercancer + df_clean$age + df_clean$gender, family = binomial, data = df_clean)

CirrvsCtrl_Livercancer_OR_tab <- as.data.frame(confint(CirrvsCtrl_Livercancer_OR))

CirrvsCtrl_Livercancer_OR_tab$OR <- summary(CirrvsCtrl_Livercancer_OR)$coefficients[,1]

CirrvsCtrl_Livercancer_OR_tab$z_score <- summary(CirrvsCtrl_Livercancer_OR)$coefficients[,3]

CirrvsCtrl_Livercancer_OR_tab$CirrvsCtrl_pval <- 2*pnorm(-abs(CirrvsCtrl_Livercancer_OR_tab$z_score))

CirrvsCtrl_Livercancer_OR_tab <- CirrvsCtrl_Livercancer_OR_tab %>% rename(lower = c(1))

CirrvsCtrl_Livercancer_OR_tab <- CirrvsCtrl_Livercancer_OR_tab %>% rename(upper = c(2))

CirrvsCtrl_Livercancer_OR_tab$CirrvsCtrl_text <- paste(format(round(CirrvsCtrl_Livercancer_OR_tab$OR, 1), nsmall = 1), format(round(CirrvsCtrl_Livercancer_OR_tab$lower, 1), nsmall = 1), sep = ' (', collapse = NULL)

CirrvsCtrl_Livercancer_OR_tab$CirrvsCtrl_text <- paste(CirrvsCtrl_Livercancer_OR_tab$CirrvsCtrl_text, format(round(CirrvsCtrl_Livercancer_OR_tab$upper, 1), nsmall = 1), sep = '-', collapse = NULL)

CirrvsCtrl_Livercancer_OR_tab$CirrvsCtrl_text <- paste(CirrvsCtrl_Livercancer_OR_tab$CirrvsCtrl_text, '', sep = ')', collapse = NULL)

CirrvsCtrl_Livercancer_OR_tab$var <- "Livercancer"

CirrvsCtrl_Livercancer_OR_tab <- CirrvsCtrl_Livercancer_OR_tab %>% slice(2)

CirrvsCtrl_Livercancer_OR_tab <- CirrvsCtrl_Livercancer_OR_tab %>% select(5:7)

CirrvsNAFLD_liver_decomp_cat_OR <- glm(df_clean$CirrvsNAFLD ~ df_clean$liver_decomp_cat + df_clean$age + df_clean$gender, family = binomial, data = df_clean)

CirrvsNAFLD_liver_decomp_cat_OR_tab <- as.data.frame(confint(CirrvsNAFLD_liver_decomp_cat_OR))

CirrvsNAFLD_liver_decomp_cat_OR_tab$OR <- summary(CirrvsNAFLD_liver_decomp_cat_OR)$coefficients[,1]

CirrvsNAFLD_liver_decomp_cat_OR_tab$z_score <- summary(CirrvsNAFLD_liver_decomp_cat_OR)$coefficients[,3]

CirrvsNAFLD_liver_decomp_cat_OR_tab$CirrvsNAFLD_pval <- 2*pnorm(-abs(CirrvsNAFLD_liver_decomp_cat_OR_tab$z_score))

CirrvsNAFLD_liver_decomp_cat_OR_tab <- CirrvsNAFLD_liver_decomp_cat_OR_tab %>% rename(lower = c(1))

CirrvsNAFLD_liver_decomp_cat_OR_tab <- CirrvsNAFLD_liver_decomp_cat_OR_tab %>% rename(upper = c(2))

CirrvsNAFLD_liver_decomp_cat_OR_tab$CirrvsNAFLD_text <- paste(format(round(CirrvsNAFLD_liver_decomp_cat_OR_tab$OR, 1), nsmall = 1), format(round(CirrvsNAFLD_liver_decomp_cat_OR_tab$lower, 1), nsmall = 1), sep = ' (', collapse = NULL)

CirrvsNAFLD_liver_decomp_cat_OR_tab$CirrvsNAFLD_text <- paste(CirrvsNAFLD_liver_decomp_cat_OR_tab$CirrvsNAFLD_text, format(round(CirrvsNAFLD_liver_decomp_cat_OR_tab$upper, 1), nsmall = 1), sep = '-', collapse = NULL)

CirrvsNAFLD_liver_decomp_cat_OR_tab$CirrvsNAFLD_text <- paste(CirrvsNAFLD_liver_decomp_cat_OR_tab$CirrvsNAFLD_text, '', sep = ')', collapse = NULL)

CirrvsNAFLD_liver_decomp_cat_OR_tab$var <- "liver_decomp_cat"

CirrvsNAFLD_liver_decomp_cat_OR_tab <- CirrvsNAFLD_liver_decomp_cat_OR_tab %>% slice(2)

CirrvsNAFLD_liver_decomp_cat_OR_tab <- CirrvsNAFLD_liver_decomp_cat_OR_tab %>% select(5:7)

CirrvsNAFLD_Livercancer_OR <- glm(df_clean$CirrvsNAFLD ~ df_clean$Livercancer + df_clean$age + df_clean$gender, family = binomial, data = df_clean)

CirrvsNAFLD_Livercancer_OR_tab <- as.data.frame(confint(CirrvsNAFLD_Livercancer_OR))

CirrvsNAFLD_Livercancer_OR_tab$OR <- summary(CirrvsNAFLD_Livercancer_OR)$coefficients[,1]

CirrvsNAFLD_Livercancer_OR_tab$z_score <- summary(CirrvsNAFLD_Livercancer_OR)$coefficients[,3]

CirrvsNAFLD_Livercancer_OR_tab$CirrvsNAFLD_pval <- 2*pnorm(-abs(CirrvsNAFLD_Livercancer_OR_tab$z_score))

CirrvsNAFLD_Livercancer_OR_tab <- CirrvsNAFLD_Livercancer_OR_tab %>% rename(lower = c(1))

CirrvsNAFLD_Livercancer_OR_tab <- CirrvsNAFLD_Livercancer_OR_tab %>% rename(upper = c(2))

CirrvsNAFLD_Livercancer_OR_tab$CirrvsNAFLD_text <- paste(format(round(CirrvsNAFLD_Livercancer_OR_tab$OR, 1), nsmall = 1), format(round(CirrvsNAFLD_Livercancer_OR_tab$lower, 1), nsmall = 1), sep = ' (', collapse = NULL)

CirrvsNAFLD_Livercancer_OR_tab$CirrvsNAFLD_text <- paste(CirrvsNAFLD_Livercancer_OR_tab$CirrvsNAFLD_text, format(round(CirrvsNAFLD_Livercancer_OR_tab$upper, 1), nsmall = 1), sep = '-', collapse = NULL)

CirrvsNAFLD_Livercancer_OR_tab$CirrvsNAFLD_text <- paste(CirrvsNAFLD_Livercancer_OR_tab$CirrvsNAFLD_text, '', sep = ')', collapse = NULL)

CirrvsNAFLD_Livercancer_OR_tab$var <- "Livercancer"

CirrvsNAFLD_Livercancer_OR_tab <- CirrvsNAFLD_Livercancer_OR_tab %>% slice(2)

CirrvsNAFLD_Livercancer_OR_tab <- CirrvsNAFLD_Livercancer_OR_tab %>% select(5:7)

## cox ph mortality

NAFLDvsCtrl_mort_demog <- coxph(Surv(mortalit, deathind) ~ NAFLDvsCtrl + age + gender, data = df_clean)

NAFLDvsCtrl_mort_demog_tab <- as.data.frame(confint(NAFLDvsCtrl_mort_demog))

NAFLDvsCtrl_mort_demog_tab$HR <- coef(NAFLDvsCtrl_mort_demog)

NAFLDvsCtrl_mort_demog_tab <- NAFLDvsCtrl_mort_demog_tab %>% rename(lower = c(1))

NAFLDvsCtrl_mort_demog_tab <- NAFLDvsCtrl_mort_demog_tab %>% rename(upper = c(2))

NAFLDvsCtrl_mort_demog_tab$HR <- exp(NAFLDvsCtrl_mort_demog_tab$HR)

NAFLDvsCtrl_mort_demog_tab$lower <- exp(NAFLDvsCtrl_mort_demog_tab$lower)

NAFLDvsCtrl_mort_demog_tab$upper <- exp(NAFLDvsCtrl_mort_demog_tab$upper)

NAFLDvsCtrl_mort_demog_tab$z_score <- summary(NAFLDvsCtrl_mort_demog)$coefficients[,4]

NAFLDvsCtrl_mort_demog_tab$NAFLDvsCtrl_pval <- 2*pnorm(-abs(NAFLDvsCtrl_mort_demog_tab$z_score))

NAFLDvsCtrl_mort_demog_tab$NAFLDvsCtrl_text <- paste(format(round(NAFLDvsCtrl_mort_demog_tab$HR, 1), nsmall = 1), format(round(NAFLDvsCtrl_mort_demog_tab$lower, 1), nsmall = 1), sep = ' (', collapse = NULL)

NAFLDvsCtrl_mort_demog_tab$NAFLDvsCtrl_text <- paste(NAFLDvsCtrl_mort_demog_tab$NAFLDvsCtrl_text, format(round(NAFLDvsCtrl_mort_demog_tab$upper, 1), nsmall = 1), sep = '-', collapse = NULL)

NAFLDvsCtrl_mort_demog_tab$NAFLDvsCtrl_text <- paste(NAFLDvsCtrl_mort_demog_tab$NAFLDvsCtrl_text, '', sep = ')', collapse = NULL)

NAFLDvsCtrl_mort_demog_tab$var <- "mort_demog"

NAFLDvsCtrl_mort_demog_tab <- NAFLDvsCtrl_mort_demog_tab %>% slice(1)

NAFLDvsCtrl_mort_demog_tab <- NAFLDvsCtrl_mort_demog_tab %>% select(5:7)

NAFLDvsCtrl_mort_CVD <- coxph(Surv(mortalit, deathind) ~ NAFLDvsCtrl + age + gender + T2DM + Obesity + HYPERLIPIDAEMIA + HEARTFAILURE + AF + CKD + IHD + MI + ISCHAEMICSTROKE + hyperten + PVD, data = df_clean)

NAFLDvsCtrl_mort_CVD_tab <- as.data.frame(confint(NAFLDvsCtrl_mort_CVD))

NAFLDvsCtrl_mort_CVD_tab$HR <- coef(NAFLDvsCtrl_mort_CVD)

NAFLDvsCtrl_mort_CVD_tab <- NAFLDvsCtrl_mort_CVD_tab %>% rename(lower = c(1))

NAFLDvsCtrl_mort_CVD_tab <- NAFLDvsCtrl_mort_CVD_tab %>% rename(upper = c(2))

NAFLDvsCtrl_mort_CVD_tab$HR <- exp(NAFLDvsCtrl_mort_CVD_tab$HR)

NAFLDvsCtrl_mort_CVD_tab$lower <- exp(NAFLDvsCtrl_mort_CVD_tab$lower)

NAFLDvsCtrl_mort_CVD_tab$upper <- exp(NAFLDvsCtrl_mort_CVD_tab$upper)

NAFLDvsCtrl_mort_CVD_tab$z_score <- summary(NAFLDvsCtrl_mort_CVD)$coefficients[,4]

NAFLDvsCtrl_mort_CVD_tab$NAFLDvsCtrl_pval <- 2*pnorm(-abs(NAFLDvsCtrl_mort_CVD_tab$z_score))

NAFLDvsCtrl_mort_CVD_tab$NAFLDvsCtrl_text <- paste(format(round(NAFLDvsCtrl_mort_CVD_tab$HR, 1), nsmall = 1), format(round(NAFLDvsCtrl_mort_CVD_tab$lower, 1), nsmall = 1), sep = ' (', collapse = NULL)

NAFLDvsCtrl_mort_CVD_tab$NAFLDvsCtrl_text <- paste(NAFLDvsCtrl_mort_CVD_tab$NAFLDvsCtrl_text, format(round(NAFLDvsCtrl_mort_CVD_tab$upper, 1), nsmall = 1), sep = '-', collapse = NULL)

NAFLDvsCtrl_mort_CVD_tab$NAFLDvsCtrl_text <- paste(NAFLDvsCtrl_mort_CVD_tab$NAFLDvsCtrl_text, '', sep = ')', collapse = NULL)

NAFLDvsCtrl_mort_CVD_tab$var <- "mort_CVD"

NAFLDvsCtrl_mort_CVD_tab <- NAFLDvsCtrl_mort_CVD_tab %>% slice(1)

NAFLDvsCtrl_mort_CVD_tab <- NAFLDvsCtrl_mort_CVD_tab %>% select(5:7)

NAFLDvsCtrl_mort_liver <- coxph(Surv(mortalit, deathind) ~ NAFLDvsCtrl + age + gender + Livercancer + liver_decomp_cat, data = df_clean)

NAFLDvsCtrl_mort_liver_tab <- as.data.frame(confint(NAFLDvsCtrl_mort_liver))

NAFLDvsCtrl_mort_liver_tab$HR <- coef(NAFLDvsCtrl_mort_liver)

NAFLDvsCtrl_mort_liver_tab <- NAFLDvsCtrl_mort_liver_tab %>% rename(lower = c(1))

NAFLDvsCtrl_mort_liver_tab <- NAFLDvsCtrl_mort_liver_tab %>% rename(upper = c(2))

NAFLDvsCtrl_mort_liver_tab$HR <- exp(NAFLDvsCtrl_mort_liver_tab$HR)

NAFLDvsCtrl_mort_liver_tab$lower <- exp(NAFLDvsCtrl_mort_liver_tab$lower)

NAFLDvsCtrl_mort_liver_tab$upper <- exp(NAFLDvsCtrl_mort_liver_tab$upper)

NAFLDvsCtrl_mort_liver_tab$z_score <- summary(NAFLDvsCtrl_mort_liver)$coefficients[,4]

NAFLDvsCtrl_mort_liver_tab$NAFLDvsCtrl_pval <- 2*pnorm(-abs(NAFLDvsCtrl_mort_liver_tab$z_score))

NAFLDvsCtrl_mort_liver_tab$NAFLDvsCtrl_text <- paste(format(round(NAFLDvsCtrl_mort_liver_tab$HR, 1), nsmall = 1), format(round(NAFLDvsCtrl_mort_liver_tab$lower, 1), nsmall = 1), sep = ' (', collapse = NULL)

NAFLDvsCtrl_mort_liver_tab$NAFLDvsCtrl_text <- paste(NAFLDvsCtrl_mort_liver_tab$NAFLDvsCtrl_text, format(round(NAFLDvsCtrl_mort_liver_tab$upper, 1), nsmall = 1), sep = '-', collapse = NULL)

NAFLDvsCtrl_mort_liver_tab$NAFLDvsCtrl_text <- paste(NAFLDvsCtrl_mort_liver_tab$NAFLDvsCtrl_text, '', sep = ')', collapse = NULL)

NAFLDvsCtrl_mort_liver_tab$var <- "mort_liver"

NAFLDvsCtrl_mort_liver_tab <- NAFLDvsCtrl_mort_liver_tab %>% slice(1)

NAFLDvsCtrl_mort_liver_tab <- NAFLDvsCtrl_mort_liver_tab %>% select(5:7)

NAFLDvsCtrl_mort_CVDLiv <- coxph(Surv(mortalit, deathind) ~ NAFLDvsCtrl + age + gender + T2DM + Obesity + HYPERLIPIDAEMIA + HEARTFAILURE + AF + CKD + IHD + MI + ISCHAEMICSTROKE + hyperten + PVD + Livercancer + liver_decomp_cat, data = df_clean)

NAFLDvsCtrl_mort_CVDLiv_tab <- as.data.frame(confint(NAFLDvsCtrl_mort_CVDLiv))

NAFLDvsCtrl_mort_CVDLiv_tab$HR <- coef(NAFLDvsCtrl_mort_CVDLiv)

NAFLDvsCtrl_mort_CVDLiv_tab <- NAFLDvsCtrl_mort_CVDLiv_tab %>% rename(lower = c(1))

NAFLDvsCtrl_mort_CVDLiv_tab <- NAFLDvsCtrl_mort_CVDLiv_tab %>% rename(upper = c(2))

NAFLDvsCtrl_mort_CVDLiv_tab$HR <- exp(NAFLDvsCtrl_mort_CVDLiv_tab$HR)

NAFLDvsCtrl_mort_CVDLiv_tab$lower <- exp(NAFLDvsCtrl_mort_CVDLiv_tab$lower)

NAFLDvsCtrl_mort_CVDLiv_tab$upper <- exp(NAFLDvsCtrl_mort_CVDLiv_tab$upper)

NAFLDvsCtrl_mort_CVDLiv_tab$z_score <- summary(NAFLDvsCtrl_mort_CVDLiv)$coefficients[,4]

NAFLDvsCtrl_mort_CVDLiv_tab$NAFLDvsCtrl_pval <- 2*pnorm(-abs(NAFLDvsCtrl_mort_CVDLiv_tab$z_score))

NAFLDvsCtrl_mort_CVDLiv_tab$NAFLDvsCtrl_text <- paste(format(round(NAFLDvsCtrl_mort_CVDLiv_tab$HR, 1), nsmall = 1), format(round(NAFLDvsCtrl_mort_CVDLiv_tab$lower, 1), nsmall = 1), sep = ' (', collapse = NULL)

NAFLDvsCtrl_mort_CVDLiv_tab$NAFLDvsCtrl_text <- paste(NAFLDvsCtrl_mort_CVDLiv_tab$NAFLDvsCtrl_text, format(round(NAFLDvsCtrl_mort_CVDLiv_tab$upper, 1), nsmall = 1), sep = '-', collapse = NULL)

NAFLDvsCtrl_mort_CVDLiv_tab$NAFLDvsCtrl_text <- paste(NAFLDvsCtrl_mort_CVDLiv_tab$NAFLDvsCtrl_text, '', sep = ')', collapse = NULL)

NAFLDvsCtrl_mort_CVDLiv_tab$var <- "mort_CVDLiv"

NAFLDvsCtrl_mort_CVDLiv_tab <- NAFLDvsCtrl_mort_CVDLiv_tab %>% slice(1)

NAFLDvsCtrl_mort_CVDLiv_tab <- NAFLDvsCtrl_mort_CVDLiv_tab %>% select(5:7)

CirrvsCtrl_mort_demog <- coxph(Surv(mortalit, deathind) ~ CirrvsCtrl + age + gender, data = df_clean)

CirrvsCtrl_mort_demog_tab <- as.data.frame(confint(CirrvsCtrl_mort_demog))

CirrvsCtrl_mort_demog_tab$HR <- coef(CirrvsCtrl_mort_demog)

CirrvsCtrl_mort_demog_tab <- CirrvsCtrl_mort_demog_tab %>% rename(lower = c(1))

CirrvsCtrl_mort_demog_tab <- CirrvsCtrl_mort_demog_tab %>% rename(upper = c(2))

CirrvsCtrl_mort_demog_tab$HR <- exp(CirrvsCtrl_mort_demog_tab$HR)

CirrvsCtrl_mort_demog_tab$lower <- exp(CirrvsCtrl_mort_demog_tab$lower)

CirrvsCtrl_mort_demog_tab$upper <- exp(CirrvsCtrl_mort_demog_tab$upper)

CirrvsCtrl_mort_demog_tab$z_score <- summary(CirrvsCtrl_mort_demog)$coefficients[,4]

CirrvsCtrl_mort_demog_tab$CirrvsCtrl_pval <- 2*pnorm(-abs(CirrvsCtrl_mort_demog_tab$z_score))

CirrvsCtrl_mort_demog_tab$CirrvsCtrl_text <- paste(format(round(CirrvsCtrl_mort_demog_tab$HR, 1), nsmall = 1), format(round(CirrvsCtrl_mort_demog_tab$lower, 1), nsmall = 1), sep = ' (', collapse = NULL)

CirrvsCtrl_mort_demog_tab$CirrvsCtrl_text <- paste(CirrvsCtrl_mort_demog_tab$CirrvsCtrl_text, format(round(CirrvsCtrl_mort_demog_tab$upper, 1), nsmall = 1), sep = '-', collapse = NULL)

CirrvsCtrl_mort_demog_tab$CirrvsCtrl_text <- paste(CirrvsCtrl_mort_demog_tab$CirrvsCtrl_text, '', sep = ')', collapse = NULL)

CirrvsCtrl_mort_demog_tab$var <- "mort_demog"

CirrvsCtrl_mort_demog_tab <- CirrvsCtrl_mort_demog_tab %>% slice(1)

CirrvsCtrl_mort_demog_tab <- CirrvsCtrl_mort_demog_tab %>% select(5:7)

CirrvsCtrl_mort_CVD <- coxph(Surv(mortalit, deathind) ~ CirrvsCtrl + age + gender + T2DM + Obesity + HYPERLIPIDAEMIA + HEARTFAILURE + AF + CKD + IHD + MI + ISCHAEMICSTROKE + hyperten + PVD, data = df_clean)

CirrvsCtrl_mort_CVD_tab <- as.data.frame(confint(CirrvsCtrl_mort_CVD))

CirrvsCtrl_mort_CVD_tab$HR <- coef(CirrvsCtrl_mort_CVD)

CirrvsCtrl_mort_CVD_tab <- CirrvsCtrl_mort_CVD_tab %>% rename(lower = c(1))

CirrvsCtrl_mort_CVD_tab <- CirrvsCtrl_mort_CVD_tab %>% rename(upper = c(2))

CirrvsCtrl_mort_CVD_tab$HR <- exp(CirrvsCtrl_mort_CVD_tab$HR)

CirrvsCtrl_mort_CVD_tab$lower <- exp(CirrvsCtrl_mort_CVD_tab$lower)

CirrvsCtrl_mort_CVD_tab$upper <- exp(CirrvsCtrl_mort_CVD_tab$upper)

CirrvsCtrl_mort_CVD_tab$z_score <- summary(CirrvsCtrl_mort_CVD)$coefficients[,4]

CirrvsCtrl_mort_CVD_tab$CirrvsCtrl_pval <- 2*pnorm(-abs(CirrvsCtrl_mort_CVD_tab$z_score))

CirrvsCtrl_mort_CVD_tab$CirrvsCtrl_text <- paste(format(round(CirrvsCtrl_mort_CVD_tab$HR, 1), nsmall = 1), format(round(CirrvsCtrl_mort_CVD_tab$lower, 1), nsmall = 1), sep = ' (', collapse = NULL)

CirrvsCtrl_mort_CVD_tab$CirrvsCtrl_text <- paste(CirrvsCtrl_mort_CVD_tab$CirrvsCtrl_text, format(round(CirrvsCtrl_mort_CVD_tab$upper, 1), nsmall = 1), sep = '-', collapse = NULL)

CirrvsCtrl_mort_CVD_tab$CirrvsCtrl_text <- paste(CirrvsCtrl_mort_CVD_tab$CirrvsCtrl_text, '', sep = ')', collapse = NULL)

CirrvsCtrl_mort_CVD_tab$var <- "mort_CVD"

CirrvsCtrl_mort_CVD_tab <- CirrvsCtrl_mort_CVD_tab %>% slice(1)

CirrvsCtrl_mort_CVD_tab <- CirrvsCtrl_mort_CVD_tab %>% select(5:7)

CirrvsCtrl_mort_liver <- coxph(Surv(mortalit, deathind) ~ CirrvsCtrl + age + gender + Livercancer + liver_decomp_cat, data = df_clean)

CirrvsCtrl_mort_liver_tab <- as.data.frame(confint(CirrvsCtrl_mort_liver))

CirrvsCtrl_mort_liver_tab$HR <- coef(CirrvsCtrl_mort_liver)

CirrvsCtrl_mort_liver_tab <- CirrvsCtrl_mort_liver_tab %>% rename(lower = c(1))

CirrvsCtrl_mort_liver_tab <- CirrvsCtrl_mort_liver_tab %>% rename(upper = c(2))

CirrvsCtrl_mort_liver_tab$HR <- exp(CirrvsCtrl_mort_liver_tab$HR)

CirrvsCtrl_mort_liver_tab$lower <- exp(CirrvsCtrl_mort_liver_tab$lower)

CirrvsCtrl_mort_liver_tab$upper <- exp(CirrvsCtrl_mort_liver_tab$upper)

CirrvsCtrl_mort_liver_tab$z_score <- summary(CirrvsCtrl_mort_liver)$coefficients[,4]

CirrvsCtrl_mort_liver_tab$CirrvsCtrl_pval <- 2*pnorm(-abs(CirrvsCtrl_mort_liver_tab$z_score))

CirrvsCtrl_mort_liver_tab$CirrvsCtrl_text <- paste(format(round(CirrvsCtrl_mort_liver_tab$HR, 1), nsmall = 1), format(round(CirrvsCtrl_mort_liver_tab$lower, 1), nsmall = 1), sep = ' (', collapse = NULL)

CirrvsCtrl_mort_liver_tab$CirrvsCtrl_text <- paste(CirrvsCtrl_mort_liver_tab$CirrvsCtrl_text, format(round(CirrvsCtrl_mort_liver_tab$upper, 1), nsmall = 1), sep = '-', collapse = NULL)

CirrvsCtrl_mort_liver_tab$CirrvsCtrl_text <- paste(CirrvsCtrl_mort_liver_tab$CirrvsCtrl_text, '', sep = ')', collapse = NULL)

CirrvsCtrl_mort_liver_tab$var <- "mort_liver"

CirrvsCtrl_mort_liver_tab <- CirrvsCtrl_mort_liver_tab %>% slice(1)

CirrvsCtrl_mort_liver_tab <- CirrvsCtrl_mort_liver_tab %>% select(5:7)

CirrvsCtrl_mort_CVDLiv <- coxph(Surv(mortalit, deathind) ~ CirrvsCtrl + age + gender + T2DM + Obesity + HYPERLIPIDAEMIA + HEARTFAILURE + AF + CKD + IHD + MI + ISCHAEMICSTROKE + hyperten + PVD + Livercancer + liver_decomp_cat, data = df_clean)

CirrvsCtrl_mort_CVDLiv_tab <- as.data.frame(confint(CirrvsCtrl_mort_CVDLiv))

CirrvsCtrl_mort_CVDLiv_tab$HR <- coef(CirrvsCtrl_mort_CVDLiv)

CirrvsCtrl_mort_CVDLiv_tab <- CirrvsCtrl_mort_CVDLiv_tab %>% rename(lower = c(1))

CirrvsCtrl_mort_CVDLiv_tab <- CirrvsCtrl_mort_CVDLiv_tab %>% rename(upper = c(2))

CirrvsCtrl_mort_CVDLiv_tab$HR <- exp(CirrvsCtrl_mort_CVDLiv_tab$HR)

CirrvsCtrl_mort_CVDLiv_tab$lower <- exp(CirrvsCtrl_mort_CVDLiv_tab$lower)

CirrvsCtrl_mort_CVDLiv_tab$upper <- exp(CirrvsCtrl_mort_CVDLiv_tab$upper)

CirrvsCtrl_mort_CVDLiv_tab$z_score <- summary(CirrvsCtrl_mort_CVDLiv)$coefficients[,4]

CirrvsCtrl_mort_CVDLiv_tab$CirrvsCtrl_pval <- 2*pnorm(-abs(CirrvsCtrl_mort_CVDLiv_tab$z_score))

CirrvsCtrl_mort_CVDLiv_tab$CirrvsCtrl_text <- paste(format(round(CirrvsCtrl_mort_CVDLiv_tab$HR, 1), nsmall = 1), format(round(CirrvsCtrl_mort_CVDLiv_tab$lower, 1), nsmall = 1), sep = ' (', collapse = NULL)

CirrvsCtrl_mort_CVDLiv_tab$CirrvsCtrl_text <- paste(CirrvsCtrl_mort_CVDLiv_tab$CirrvsCtrl_text, format(round(CirrvsCtrl_mort_CVDLiv_tab$upper, 1), nsmall = 1), sep = '-', collapse = NULL)

CirrvsCtrl_mort_CVDLiv_tab$CirrvsCtrl_text <- paste(CirrvsCtrl_mort_CVDLiv_tab$CirrvsCtrl_text, '', sep = ')', collapse = NULL)

CirrvsCtrl_mort_CVDLiv_tab$var <- "mort_CVDLiv"

CirrvsCtrl_mort_CVDLiv_tab <- CirrvsCtrl_mort_CVDLiv_tab %>% slice(1)

CirrvsCtrl_mort_CVDLiv_tab <- CirrvsCtrl_mort_CVDLiv_tab %>% select(5:7)

CirrvsNAFLD_mort_demog <- coxph(Surv(mortalit, deathind) ~ CirrvsNAFLD + age + gender, data = df_clean)

CirrvsNAFLD_mort_demog_tab <- as.data.frame(confint(CirrvsNAFLD_mort_demog))

CirrvsNAFLD_mort_demog_tab$HR <- coef(CirrvsNAFLD_mort_demog)

CirrvsNAFLD_mort_demog_tab <- CirrvsNAFLD_mort_demog_tab %>% rename(lower = c(1))

CirrvsNAFLD_mort_demog_tab <- CirrvsNAFLD_mort_demog_tab %>% rename(upper = c(2))

CirrvsNAFLD_mort_demog_tab$HR <- exp(CirrvsNAFLD_mort_demog_tab$HR)

CirrvsNAFLD_mort_demog_tab$lower <- exp(CirrvsNAFLD_mort_demog_tab$lower)

CirrvsNAFLD_mort_demog_tab$upper <- exp(CirrvsNAFLD_mort_demog_tab$upper)

CirrvsNAFLD_mort_demog_tab$z_score <- summary(CirrvsNAFLD_mort_demog)$coefficients[,4]

CirrvsNAFLD_mort_demog_tab$CirrvsNAFLD_pval <- 2*pnorm(-abs(CirrvsNAFLD_mort_demog_tab$z_score))

CirrvsNAFLD_mort_demog_tab$CirrvsNAFLD_text <- paste(format(round(CirrvsNAFLD_mort_demog_tab$HR, 1), nsmall = 1), format(round(CirrvsNAFLD_mort_demog_tab$lower, 1), nsmall = 1), sep = ' (', collapse = NULL)

CirrvsNAFLD_mort_demog_tab$CirrvsNAFLD_text <- paste(CirrvsNAFLD_mort_demog_tab$CirrvsNAFLD_text, format(round(CirrvsNAFLD_mort_demog_tab$upper, 1), nsmall = 1), sep = '-', collapse = NULL)

CirrvsNAFLD_mort_demog_tab$CirrvsNAFLD_text <- paste(CirrvsNAFLD_mort_demog_tab$CirrvsNAFLD_text, '', sep = ')', collapse = NULL)

CirrvsNAFLD_mort_demog_tab$var <- "mort_demog"

CirrvsNAFLD_mort_demog_tab <- CirrvsNAFLD_mort_demog_tab %>% slice(1)

CirrvsNAFLD_mort_demog_tab <- CirrvsNAFLD_mort_demog_tab %>% select(5:7)

CirrvsNAFLD_mort_CVD <- coxph(Surv(mortalit, deathind) ~ CirrvsNAFLD + age + gender + T2DM + Obesity + HYPERLIPIDAEMIA + HEARTFAILURE + AF + CKD + IHD + MI + ISCHAEMICSTROKE + hyperten + PVD, data = df_clean)

CirrvsNAFLD_mort_CVD_tab <- as.data.frame(confint(CirrvsNAFLD_mort_CVD))

CirrvsNAFLD_mort_CVD_tab$HR <- coef(CirrvsNAFLD_mort_CVD)

CirrvsNAFLD_mort_CVD_tab <- CirrvsNAFLD_mort_CVD_tab %>% rename(lower = c(1))

CirrvsNAFLD_mort_CVD_tab <- CirrvsNAFLD_mort_CVD_tab %>% rename(upper = c(2))

CirrvsNAFLD_mort_CVD_tab$HR <- exp(CirrvsNAFLD_mort_CVD_tab$HR)

CirrvsNAFLD_mort_CVD_tab$lower <- exp(CirrvsNAFLD_mort_CVD_tab$lower)

CirrvsNAFLD_mort_CVD_tab$upper <- exp(CirrvsNAFLD_mort_CVD_tab$upper)

CirrvsNAFLD_mort_CVD_tab$z_score <- summary(CirrvsNAFLD_mort_CVD)$coefficients[,4]

CirrvsNAFLD_mort_CVD_tab$CirrvsNAFLD_pval <- 2*pnorm(-abs(CirrvsNAFLD_mort_CVD_tab$z_score))

CirrvsNAFLD_mort_CVD_tab$CirrvsNAFLD_text <- paste(format(round(CirrvsNAFLD_mort_CVD_tab$HR, 1), nsmall = 1), format(round(CirrvsNAFLD_mort_CVD_tab$lower, 1), nsmall = 1), sep = ' (', collapse = NULL)

CirrvsNAFLD_mort_CVD_tab$CirrvsNAFLD_text <- paste(CirrvsNAFLD_mort_CVD_tab$CirrvsNAFLD_text, format(round(CirrvsNAFLD_mort_CVD_tab$upper, 1), nsmall = 1), sep = '-', collapse = NULL)

CirrvsNAFLD_mort_CVD_tab$CirrvsNAFLD_text <- paste(CirrvsNAFLD_mort_CVD_tab$CirrvsNAFLD_text, '', sep = ')', collapse = NULL)

CirrvsNAFLD_mort_CVD_tab$var <- "mort_CVD"

CirrvsNAFLD_mort_CVD_tab <- CirrvsNAFLD_mort_CVD_tab %>% slice(1)

CirrvsNAFLD_mort_CVD_tab <- CirrvsNAFLD_mort_CVD_tab %>% select(5:7)

CirrvsNAFLD_mort_liver <- coxph(Surv(mortalit, deathind) ~ CirrvsNAFLD + age + gender + Livercancer + liver_decomp_cat, data = df_clean)

CirrvsNAFLD_mort_liver_tab <- as.data.frame(confint(CirrvsNAFLD_mort_liver))

CirrvsNAFLD_mort_liver_tab$HR <- coef(CirrvsNAFLD_mort_liver)

CirrvsNAFLD_mort_liver_tab <- CirrvsNAFLD_mort_liver_tab %>% rename(lower = c(1))

CirrvsNAFLD_mort_liver_tab <- CirrvsNAFLD_mort_liver_tab %>% rename(upper = c(2))

CirrvsNAFLD_mort_liver_tab$HR <- exp(CirrvsNAFLD_mort_liver_tab$HR)

CirrvsNAFLD_mort_liver_tab$lower <- exp(CirrvsNAFLD_mort_liver_tab$lower)

CirrvsNAFLD_mort_liver_tab$upper <- exp(CirrvsNAFLD_mort_liver_tab$upper)

CirrvsNAFLD_mort_liver_tab$z_score <- summary(CirrvsNAFLD_mort_liver)$coefficients[,4]

CirrvsNAFLD_mort_liver_tab$CirrvsNAFLD_pval <- 2*pnorm(-abs(CirrvsNAFLD_mort_liver_tab$z_score))

CirrvsNAFLD_mort_liver_tab$CirrvsNAFLD_text <- paste(format(round(CirrvsNAFLD_mort_liver_tab$HR, 1), nsmall = 1), format(round(CirrvsNAFLD_mort_liver_tab$lower, 1), nsmall = 1), sep = ' (', collapse = NULL)

CirrvsNAFLD_mort_liver_tab$CirrvsNAFLD_text <- paste(CirrvsNAFLD_mort_liver_tab$CirrvsNAFLD_text, format(round(CirrvsNAFLD_mort_liver_tab$upper, 1), nsmall = 1), sep = '-', collapse = NULL)

CirrvsNAFLD_mort_liver_tab$CirrvsNAFLD_text <- paste(CirrvsNAFLD_mort_liver_tab$CirrvsNAFLD_text, '', sep = ')', collapse = NULL)

CirrvsNAFLD_mort_liver_tab$var <- "mort_liver"

CirrvsNAFLD_mort_liver_tab <- CirrvsNAFLD_mort_liver_tab %>% slice(1)

CirrvsNAFLD_mort_liver_tab <- CirrvsNAFLD_mort_liver_tab %>% select(5:7)

CirrvsNAFLD_mort_CVDLiv <- coxph(Surv(mortalit, deathind) ~ CirrvsNAFLD + age + gender + T2DM + Obesity + HYPERLIPIDAEMIA + HEARTFAILURE + AF + CKD + IHD + MI + ISCHAEMICSTROKE + hyperten + PVD + Livercancer + liver_decomp_cat, data = df_clean)

CirrvsNAFLD_mort_CVDLiv_tab <- as.data.frame(confint(CirrvsNAFLD_mort_CVDLiv))

CirrvsNAFLD_mort_CVDLiv_tab$HR <- coef(CirrvsNAFLD_mort_CVDLiv)

CirrvsNAFLD_mort_CVDLiv_tab <- CirrvsNAFLD_mort_CVDLiv_tab %>% rename(lower = c(1))

CirrvsNAFLD_mort_CVDLiv_tab <- CirrvsNAFLD_mort_CVDLiv_tab %>% rename(upper = c(2))

CirrvsNAFLD_mort_CVDLiv_tab$HR <- exp(CirrvsNAFLD_mort_CVDLiv_tab$HR)

CirrvsNAFLD_mort_CVDLiv_tab$lower <- exp(CirrvsNAFLD_mort_CVDLiv_tab$lower)

CirrvsNAFLD_mort_CVDLiv_tab$upper <- exp(CirrvsNAFLD_mort_CVDLiv_tab$upper)

CirrvsNAFLD_mort_CVDLiv_tab$z_score <- summary(CirrvsNAFLD_mort_CVDLiv)$coefficients[,4]

CirrvsNAFLD_mort_CVDLiv_tab$CirrvsNAFLD_pval <- 2*pnorm(-abs(CirrvsNAFLD_mort_CVDLiv_tab$z_score))

CirrvsNAFLD_mort_CVDLiv_tab$CirrvsNAFLD_text <- paste(format(round(CirrvsNAFLD_mort_CVDLiv_tab$HR, 1), nsmall = 1), format(round(CirrvsNAFLD_mort_CVDLiv_tab$lower, 1), nsmall = 1), sep = ' (', collapse = NULL)

CirrvsNAFLD_mort_CVDLiv_tab$CirrvsNAFLD_text <- paste(CirrvsNAFLD_mort_CVDLiv_tab$CirrvsNAFLD_text, format(round(CirrvsNAFLD_mort_CVDLiv_tab$upper, 1), nsmall = 1), sep = '-', collapse = NULL)

CirrvsNAFLD_mort_CVDLiv_tab$CirrvsNAFLD_text <- paste(CirrvsNAFLD_mort_CVDLiv_tab$CirrvsNAFLD_text, '', sep = ')', collapse = NULL)

CirrvsNAFLD_mort_CVDLiv_tab$var <- "mort_CVDLiv"

CirrvsNAFLD_mort_CVDLiv_tab <- CirrvsNAFLD_mort_CVDLiv_tab %>% slice(1)

CirrvsNAFLD_mort_CVDLiv_tab <- CirrvsNAFLD_mort_CVDLiv_tab %>% select(5:7)

Livercancer_OR_sumtab1 <- merge(NAFLDvsCtrl_Livercancer_OR_tab, CirrvsCtrl_Livercancer_OR_tab, by="var")

Livercancer_OR_sumtab1 <- merge(Livercancer_OR_sumtab1, CirrvsNAFLD_Livercancer_OR_tab, by="var")

liver_decomp_cat_OR_sumtab1 <- merge(NAFLDvsCtrl_liver_decomp_cat_OR_tab, CirrvsCtrl_liver_decomp_cat_OR_tab, by="var")

liver_decomp_cat_OR_sumtab1 <- merge(liver_decomp_cat_OR_sumtab1, CirrvsNAFLD_liver_decomp_cat_OR_tab, by="var")

mort_demog_sumtab1 <- merge(NAFLDvsCtrl_mort_demog_tab, CirrvsCtrl_mort_demog_tab, by="var")

mort_demog_sumtab1 <- merge(mort_demog_sumtab1, CirrvsNAFLD_mort_demog_tab, by="var")

mort_CVD_sumtab1 <- merge(NAFLDvsCtrl_mort_CVD_tab, CirrvsCtrl_mort_CVD_tab, by="var")

mort_CVD_sumtab1 <- merge(mort_CVD_sumtab1, CirrvsNAFLD_mort_CVD_tab, by="var")

mort_liver_sumtab1 <- merge(NAFLDvsCtrl_mort_liver_tab, CirrvsCtrl_mort_liver_tab, by="var")

mort_liver_sumtab1 <- merge(mort_liver_sumtab1, CirrvsNAFLD_mort_liver_tab, by="var")

mort_CVDLiv_sumtab1 <- merge(NAFLDvsCtrl_mort_CVDLiv_tab, CirrvsCtrl_mort_CVDLiv_tab, by="var")

mort_CVDLiv_sumtab1 <- merge(mort_CVDLiv_sumtab1, CirrvsNAFLD_mort_CVDLiv_tab, by="var")

OR_sumtab1 <- rbind(liver_decomp_cat_OR_sumtab1, Livercancer_OR_sumtab1, mort_demog_sumtab1, mort_CVD_sumtab1, mort_liver_sumtab1, mort_CVDLiv_sumtab1)

OR_sumtab1$NAFLDvsCtrl_qval <- p.adjust(OR_sumtab1$NAFLDvsCtrl_pval, method = "BH")

OR_sumtab1$CirrvsCtrl_qval <- p.adjust(OR_sumtab1$CirrvsCtrl_pval, method = "BH")

OR_sumtab1$CirrvsNAFLD_qval <- p.adjust(OR_sumtab1$CirrvsNAFLD_pval, method = "BH")

OR_sumtab1$NAFLDvsCtrl_qval <- ifelse(OR_sumtab1$NAFLDvsCtrl_qval <.01, format(OR_sumtab1$NAFLDvsCtrl_qval, scientific=T, digits=2), format(round(OR_sumtab1$NAFLDvsCtrl_qval, 2), nsmall = 2))

OR_sumtab1$CirrvsCtrl_qval <- ifelse(OR_sumtab1$CirrvsCtrl_qval <.01, format(OR_sumtab1$CirrvsCtrl_qval, scientific=T, digits=2), format(round(OR_sumtab1$CirrvsCtrl_qval, 2), nsmall = 2))

OR_sumtab1$CirrvsNAFLD_qval <- ifelse(OR_sumtab1$CirrvsNAFLD_qval <.01, format(OR_sumtab1$CirrvsNAFLD_qval, scientific=T, digits=2), format(round(OR_sumtab1$CirrvsNAFLD_qval, 2), nsmall = 2))

write.table(OR_sumtab1, file="OR_sumtab1.csv", sep=",")

############

## make figures

fig_demog <- coxph(Surv(mortalit, deathind) ~ strata(group1) + age + gender, data = df_clean)

fig_demog_fit <- survfit(fig_demog, strata = "group1")

fig_demog_curve <- ggsurvplot(fig_demog_fit, data = df_clean, variable = "group1", risk.table = TRUE, conf.int = TRUE, legend.title = "", fun = "cumhaz", fontsize=3, ylim=c(0,.8), xlab="Follow-up (days)", ylab="Cumulative hazard \n of death")

fig_demog_curve

pdf(file="fig_demog_curve.pdf",width=6,height=6)

fig_demog_curve

dev.off()

fig_liver <- coxph(Surv(mortalit, deathind) ~ strata(group1) + age + gender + Livercancer + liver_decomp_cat, data = df_clean)

fig_liver_fit <- survfit(fig_liver, strata = "group1")

fig_liver_curve <- ggsurvplot(fig_liver_fit, data = df_clean, variable = "group1", risk.table = TRUE, conf.int = TRUE, legend.title = "", fun = "cumhaz", fontsize=3, ylim=c(0,.8), xlab="Follow-up (days)", ylab="Cumulative hazard \n of death")

pdf(file="fig_liver_curve.pdf",width=6,height=6)

fig_liver_curve

dev.off()

fig_CVDliver <- coxph(Surv(mortalit, deathind) ~ strata(group1) + age + gender + T2DM + Obesity + HYPERLIPIDAEMIA + HEARTFAILURE + AF + CKD + IHD + MI + ISCHAEMICSTROKE + hyperten + PVD + Livercancer + liver_decomp_cat, data = df_clean)

fig_CVDliver_fit <- survfit(fig_CVDliver, strata = "group1")

fig_CVDliver_curve <- ggsurvplot(fig_CVDliver_fit, data = df_clean, variable = "group1", risk.table = TRUE, conf.int = TRUE, legend.title = "", fun = "cumhaz", fontsize=3, ylim=c(0,.8), xlab="Follow-up (days)", ylab="Cumulative hazard \n of death")

pdf(file="fig_CVDliver_curve.pdf",width=6,height=6)

fig_CVDliver_curve

dev.off()

fig_CVD <- coxph(Surv(mortalit, deathind) ~ strata(group1) + age + gender + T2DM + Obesity + HYPERLIPIDAEMIA + HEARTFAILURE + AF + CKD + IHD + MI + ISCHAEMICSTROKE + hyperten + PVD, data = df_clean)

fig_CVD_fit <- survfit(fig_CVD, strata = "group1")

fig_CVD_curve <- ggsurvplot(fig_CVD_fit, data = df_clean, variable = "group1", risk.table = TRUE, conf.int = TRUE, legend.title = "", fun = "cumhaz", fontsize=3, ylim=c(0,.8), xlab="Follow-up (days)", ylab="Cumulative hazard \n of death")

pdf(file="fig_CVD_curve.pdf",width=6,height=6)

fig_CVD_curve

dev.off()

## figures for NAFL & NASH

fig2_demog <- coxph(Surv(mortalit, deathind) ~ strata(group2) + age + gender, data = df_clean)

fig2_demog_fit <- survfit(fig2_demog, strata = "group2")

fig2_demog_curve <- ggsurvplot(fig2_demog_fit, data = df_clean, variable = "group2", risk.table = TRUE, conf.int = TRUE, legend.title = "", fun = "cumhaz", fontsize=3, ylim=c(0,.8), xlab="Follow-up (days)", ylab="Cumulative hazard \n of death")

fig2_demog_curve

pdf(file="fig2_demog_curve.pdf",width=6,height=6)

fig2_demog_curve

dev.off()

fig2_liver <- coxph(Surv(mortalit, deathind) ~ strata(group2) + age + gender + Livercancer + liver_decomp_cat, data = df_clean)

fig2_liver_fit <- survfit(fig2_liver, strata = "group2")

fig2_liver_curve <- ggsurvplot(fig2_liver_fit, data = df_clean, variable = "group2", risk.table = TRUE, conf.int = TRUE, legend.title = "", fun = "cumhaz", fontsize=3, ylim=c(0,.8), xlab="Follow-up (days)", ylab="Cumulative hazard \n of death")

pdf(file="fig2_liver_curve.pdf",width=6,height=6)

fig2_liver_curve

dev.off()

fig2_CVDliver <- coxph(Surv(mortalit, deathind) ~ strata(group2) + age + gender + T2DM + Obesity + HYPERLIPIDAEMIA + HEARTFAILURE + AF + CKD + IHD + MI + ISCHAEMICSTROKE + hyperten + PVD + Livercancer + liver_decomp_cat, data = df_clean)

fig2_CVDliver_fit <- survfit(fig2_CVDliver, strata = "group2")

fig2_CVDliver_curve <- ggsurvplot(fig2_CVDliver_fit, data = df_clean, variable = "group2", risk.table = TRUE, conf.int = TRUE, legend.title = "", fun = "cumhaz", fontsize=3, ylim=c(0,.8), xlab="Follow-up (days)", ylab="Cumulative hazard \n of death")

pdf(file="fig2_CVDliver_curve.pdf",width=6,height=6)

fig2_CVDliver_curve

dev.off()

fig2_CVD <- coxph(Surv(mortalit, deathind) ~ strata(group2) + age + gender + T2DM + Obesity + HYPERLIPIDAEMIA + HEARTFAILURE + AF + CKD + IHD + MI + ISCHAEMICSTROKE + hyperten + PVD, data = df_clean)

fig2_CVD_fit <- survfit(fig2_CVD, strata = "group2")

fig2_CVD_curve <- ggsurvplot(fig2_CVD_fit, data = df_clean, variable = "group2", risk.table = TRUE, conf.int = TRUE, legend.title = "", fun = "cumhaz", fontsize=3, ylim=c(0,.8), xlab="Follow-up (days)", ylab="Cumulative hazard \n of death")

pdf(file="fig2_CVD_curve.pdf",width=6,height=6)

fig2_CVD_curve

dev.off()

########################

#### make tables for NAFL & NASH separately (group2)

## descriptive stats table for main analysis (group2)

group2_sumtab <- df_clean %>% group_by(group2) %>% summarise(age_mean = mean(age), age_sd = sd(age), gender = sum(gender=="F"), White = sum(ethinc_group=="White"), South_Asian = sum(ethinc_group=="Asian"), total = n(), Obesity = sum(Obesity=='1'), T2DM = sum(T2DM=='1'), HYPERLIPIDAEMIA = sum(HYPERLIPIDAEMIA=='1'), HEARTFAILURE = sum(HEARTFAILURE=='1'), AF = sum(AF=='1'), CKD = sum(CKD=='1'), IHD = sum(IHD=='1'), MI = sum(MI=='1'), carotidarterydisease = sum(carotidarterydisease=='1'), ISCHAEMICSTROKE = sum(ISCHAEMICSTROKE=='1'), hyperten = sum(hyperten=='1'), PVD = sum(PVD=='1'), Livercancer = sum(Livercancer=='1'), liver_decomp_cat = sum(liver_decomp_cat=='1'), GI_cancer = sum(GI_cancer=='1'), nonGI_cancer = sum(nonGI_cancer=='1'), all_cancer = sum(all_cancer=='1'), deathind = sum(deathind=='1'))

group2_sumtab$gender_per = (group2_sumtab$gender/group2_sumtab$total*100)

group2_sumtab$White_per = (group2_sumtab$White/group2_sumtab$total*100)

group2_sumtab$South_Asian_per = (group2_sumtab$South_Asian/group2_sumtab$total*100)

group2_sumtab$Obesity_per = (group2_sumtab$Obesity/group2_sumtab$total*100)

group2_sumtab$T2DM_per = (group2_sumtab$T2DM/group2_sumtab$total*100)

group2_sumtab$HYPERLIPIDAEMIA_per = (group2_sumtab$HYPERLIPIDAEMIA/group2_sumtab$total*100)

group2_sumtab$HEARTFAILURE_per = (group2_sumtab$HEARTFAILURE/group2_sumtab$total*100)

group2_sumtab$AF_per = (group2_sumtab$AF/group2_sumtab$total*100)

group2_sumtab$CKD_per = (group2_sumtab$CKD/group2_sumtab$total*100)

group2_sumtab$IHD_per = (group2_sumtab$IHD/group2_sumtab$total*100)

group2_sumtab$MI_per = (group2_sumtab$MI/group2_sumtab$total*100)

group2_sumtab$carotidarterydisease_per = (group2_sumtab$carotidarterydisease/group2_sumtab$total*100)

group2_sumtab$ISCHAEMICSTROKE_per = (group2_sumtab$ISCHAEMICSTROKE/group2_sumtab$total*100)

group2_sumtab$hyperten_per = (group2_sumtab$hyperten/group2_sumtab$total*100)

group2_sumtab$PVD_per = (group2_sumtab$PVD/group2_sumtab$total*100)

group2_sumtab$Livercancer_per = (group2_sumtab$Livercancer/group2_sumtab$total*100)

group2_sumtab$liver_decomp_cat_per = (group2_sumtab$liver_decomp_cat/group2_sumtab$total*100)

group2_sumtab$GI_cancer_per = (group2_sumtab$GI_cancer/group2_sumtab$total*100)

group2_sumtab$nonGI_cancer_per = (group2_sumtab$nonGI_cancer/group2_sumtab$total*100)

group2_sumtab$all_cancer_per = (group2_sumtab$all_cancer/group2_sumtab$total*100)

group2_sumtab$deathind_per = (group2_sumtab$deathind/group2_sumtab$total*100)

group2_sumtab$age_mean_text <- paste(format(round(group2_sumtab$age_mean, 1), nsmall = 1), format(round(group2_sumtab$age_sd, 1), nsmall = 1), sep = ' (', collapse = NULL)

group2_sumtab$gender_text <- paste(group2_sumtab$gender, format(round(group2_sumtab$gender_per, 1), nsmall = 1), sep = ' (', collapse = NULL)

group2_sumtab$White_text <- paste(group2_sumtab$White, format(round(group2_sumtab$White_per, 1), nsmall = 1), sep = ' (', collapse = NULL)

group2_sumtab$South_Asian_text <- paste(group2_sumtab$South_Asian, format(round(group2_sumtab$South_Asian_per, 1), nsmall = 1), sep = ' (', collapse = NULL)

group2_sumtab$Obesity_text <- paste(group2_sumtab$Obesity, format(round(group2_sumtab$Obesity_per, 1), nsmall = 1), sep = ' (', collapse = NULL)

group2_sumtab$T2DM_text <- paste(group2_sumtab$T2DM, format(round(group2_sumtab$T2DM_per, 1), nsmall = 1), sep = ' (', collapse = NULL)

group2_sumtab$HYPERLIPIDAEMIA_text <- paste(group2_sumtab$HYPERLIPIDAEMIA, format(round(group2_sumtab$HYPERLIPIDAEMIA_per, 1), nsmall = 1), sep = ' (', collapse = NULL)

group2_sumtab$HEARTFAILURE_text <- paste(group2_sumtab$HEARTFAILURE, format(round(group2_sumtab$HEARTFAILURE_per, 1), nsmall = 1), sep = ' (', collapse = NULL)

group2_sumtab$AF_text <- paste(group2_sumtab$AF, format(round(group2_sumtab$AF_per, 1), nsmall = 1), sep = ' (', collapse = NULL)

group2_sumtab$CKD_text <- paste(group2_sumtab$CKD, format(round(group2_sumtab$CKD_per, 1), nsmall = 1), sep = ' (', collapse = NULL)

group2_sumtab$IHD_text <- paste(group2_sumtab$IHD, format(round(group2_sumtab$IHD_per, 1), nsmall = 1), sep = ' (', collapse = NULL)

group2_sumtab$MI_text <- paste(group2_sumtab$MI, format(round(group2_sumtab$MI_per, 1), nsmall = 1), sep = ' (', collapse = NULL)

group2_sumtab$carotidarterydisease_text <- paste(group2_sumtab$carotidarterydisease, format(round(group2_sumtab$carotidarterydisease_per, 1), nsmall = 1), sep = ' (', collapse = NULL)

group2_sumtab$ISCHAEMICSTROKE_text <- paste(group2_sumtab$ISCHAEMICSTROKE, format(round(group2_sumtab$ISCHAEMICSTROKE_per, 1), nsmall = 1), sep = ' (', collapse = NULL)

group2_sumtab$hyperten_text <- paste(group2_sumtab$hyperten, format(round(group2_sumtab$hyperten_per, 1), nsmall = 1), sep = ' (', collapse = NULL)

group2_sumtab$PVD_text <- paste(group2_sumtab$PVD, format(round(group2_sumtab$PVD_per, 1), nsmall = 1), sep = ' (', collapse = NULL)

group2_sumtab$Livercancer_text <- paste(group2_sumtab$Livercancer, format(round(group2_sumtab$Livercancer_per, 1), nsmall = 1), sep = ' (', collapse = NULL)

group2_sumtab$liver_decomp_cat_text <- paste(group2_sumtab$liver_decomp_cat, format(round(group2_sumtab$liver_decomp_cat_per, 1), nsmall = 1), sep = ' (', collapse = NULL)

group2_sumtab$GI_cancer_text <- paste(group2_sumtab$GI_cancer, format(round(group2_sumtab$GI_cancer_per, 1), nsmall = 1), sep = ' (', collapse = NULL)

group2_sumtab$nonGI_cancer_text <- paste(group2_sumtab$nonGI_cancer, format(round(group2_sumtab$nonGI_cancer_per, 1), nsmall = 1), sep = ' (', collapse = NULL)

group2_sumtab$all_cancer_text <- paste(group2_sumtab$all_cancer, format(round(group2_sumtab$all_cancer_per, 1), nsmall = 1), sep = ' (', collapse = NULL)

group2_sumtab$deathind_text <- paste(group2_sumtab$deathind, format(round(group2_sumtab$deathind_per, 1), nsmall = 1), sep = ' (', collapse = NULL)

group2_sumtab$age_mean <- paste(group2_sumtab$age_mean_text, '', sep = ')', collapse = NULL)

group2_sumtab$gender <- paste(group2_sumtab$gender_text, '', sep = ')', collapse = NULL)

group2_sumtab$White <- paste(group2_sumtab$White_text, '', sep = ')', collapse = NULL)

group2_sumtab$South_Asian <- paste(group2_sumtab$South_Asian_text, '', sep = ')', collapse = NULL)

group2_sumtab$Obesity <- paste(group2_sumtab$Obesity_text, '', sep = ')', collapse = NULL)

group2_sumtab$T2DM <- paste(group2_sumtab$T2DM_text, '', sep = ')', collapse = NULL)

group2_sumtab$HYPERLIPIDAEMIA <- paste(group2_sumtab$HYPERLIPIDAEMIA_text, '', sep = ')', collapse = NULL)

group2_sumtab$HEARTFAILURE <- paste(group2_sumtab$HEARTFAILURE_text, '', sep = ')', collapse = NULL)

group2_sumtab$AF <- paste(group2_sumtab$AF_text, '', sep = ')', collapse = NULL)

group2_sumtab$CKD <- paste(group2_sumtab$CKD_text, '', sep = ')', collapse = NULL)

group2_sumtab$IHD <- paste(group2_sumtab$IHD_text, '', sep = ')', collapse = NULL)

group2_sumtab$MI <- paste(group2_sumtab$MI_text, '', sep = ')', collapse = NULL)

group2_sumtab$carotidarterydisease <- paste(group2_sumtab$carotidarterydisease_text, '', sep = ')', collapse = NULL)

group2_sumtab$ISCHAEMICSTROKE <- paste(group2_sumtab$ISCHAEMICSTROKE_text, '', sep = ')', collapse = NULL)

group2_sumtab$hyperten <- paste(group2_sumtab$hyperten_text, '', sep = ')', collapse = NULL)

group2_sumtab$PVD <- paste(group2_sumtab$PVD_text, '', sep = ')', collapse = NULL)

group2_sumtab$Livercancer <- paste(group2_sumtab$Livercancer_text, '', sep = ')', collapse = NULL)

group2_sumtab$liver_decomp_cat <- paste(group2_sumtab$liver_decomp_cat_text, '', sep = ')', collapse = NULL)

group2_sumtab$GI_cancer <- paste(group2_sumtab$GI_cancer_text, '', sep = ')', collapse = NULL)

group2_sumtab$nonGI_cancer <- paste(group2_sumtab$nonGI_cancer_text, '', sep = ')', collapse = NULL)

group2_sumtab$all_cancer <- paste(group2_sumtab$all_cancer_text, '', sep = ')', collapse = NULL)

group2_sumtab$deathind <- paste(group2_sumtab$deathind_text, '', sep = ')', collapse = NULL)

rownames(group2_sumtab) = group2_sumtab$group2

group2_sumtab_t <- as.data.frame(t(group2_sumtab))

group2_sumtab_t <- group2_sumtab_t[-c(1),]

group2_sumtab_t$var <- rownames(group2_sumtab_t)

### Run chi-squared tests

NAFLvsCtrl_age <- t.test(age ~ NAFLvsCtrl, data = df_clean)

NAFLvsCtrl_female <- chisq.test(x = table(df_clean$NAFLvsCtrl, df_clean$female))

NAFLvsCtrl_White_count <- chisq.test(x = table(df_clean$NAFLvsCtrl, df_clean$White_count))

NAFLvsCtrl_Asian_count <- chisq.test(x = table(df_clean$NAFLvsCtrl, df_clean$Asian_count))

NAFLvsCtrl_Obesity <- chisq.test(x = table(df_clean$NAFLvsCtrl, df_clean$Obesity))

NAFLvsCtrl_T2DM <- chisq.test(x = table(df_clean$NAFLvsCtrl, df_clean$T2DM))

NAFLvsCtrl_HYPERLIPIDAEMIA <- chisq.test(x = table(df_clean$NAFLvsCtrl, df_clean$HYPERLIPIDAEMIA))

NAFLvsCtrl_HEARTFAILURE <- chisq.test(x = table(df_clean$NAFLvsCtrl, df_clean$HEARTFAILURE))

NAFLvsCtrl_AF <- chisq.test(x = table(df_clean$NAFLvsCtrl, df_clean$AF))

NAFLvsCtrl_CKD <- chisq.test(x = table(df_clean$NAFLvsCtrl, df_clean$CKD))

NAFLvsCtrl_IHD <- chisq.test(x = table(df_clean$NAFLvsCtrl, df_clean$IHD))

NAFLvsCtrl_MI <- chisq.test(x = table(df_clean$NAFLvsCtrl, df_clean$MI))

NAFLvsCtrl_carotidarterydisease <- chisq.test(x = table(df_clean$NAFLvsCtrl, df_clean$carotidarterydisease))

NAFLvsCtrl_ISCHAEMICSTROKE <- chisq.test(x = table(df_clean$NAFLvsCtrl, df_clean$ISCHAEMICSTROKE))

NAFLvsCtrl_hyperten <- chisq.test(x = table(df_clean$NAFLvsCtrl, df_clean$hyperten))

NAFLvsCtrl_PVD <- chisq.test(x = table(df_clean$NAFLvsCtrl, df_clean$PVD))

NAFLvsCtrl_Livercancer <- chisq.test(x = table(df_clean$NAFLvsCtrl, df_clean$Livercancer))

NAFLvsCtrl_liver_decomp_cat <- chisq.test(x = table(df_clean$NAFLvsCtrl, df_clean$liver_decomp_cat))

NAFLvsCtrl_GI_cancer <- chisq.test(x = table(df_clean$NAFLvsCtrl, df_clean$GI_cancer))

NAFLvsCtrl_nonGI_cancer <- chisq.test(x = table(df_clean$NAFLvsCtrl, df_clean$nonGI_cancer))

NAFLvsCtrl_all_cancer <- chisq.test(x = table(df_clean$NAFLvsCtrl, df_clean$all_cancer))

NAFLvsCtrl_deathind <- chisq.test(x = table(df_clean$NAFLvsCtrl, df_clean$deathind))

NAFLvsCtrl_pval <- data.frame(c(NAFLvsCtrl_age$p.value, NA, NAFLvsCtrl_female$p.value, NAFLvsCtrl_White_count$p.value, NAFLvsCtrl_Asian_count$p.value, "1", NAFLvsCtrl_Obesity$p.value, NAFLvsCtrl_T2DM$p.value, NAFLvsCtrl_HYPERLIPIDAEMIA$p.value, NAFLvsCtrl_HEARTFAILURE$p.value, NAFLvsCtrl_AF$p.value, NAFLvsCtrl_CKD$p.value, NAFLvsCtrl_IHD$p.value, NAFLvsCtrl_MI$p.value, NAFLvsCtrl_carotidarterydisease$p.value, NAFLvsCtrl_ISCHAEMICSTROKE$p.value, NAFLvsCtrl_hyperten$p.value, NAFLvsCtrl_PVD$p.value, NAFLvsCtrl_Livercancer$p.value, NAFLvsCtrl_liver_decomp_cat$p.value, NAFLvsCtrl_GI_cancer$p.value, NAFLvsCtrl_nonGI_cancer$p.value, NAFLvsCtrl_all_cancer$p.value, NAFLvsCtrl_deathind$p.value))

NAFLvsCtrl_pval <- NAFLvsCtrl_pval %>% rename(NAFLvsCtrl_pval = c(1))

NAFLvsCtrl_pval$var <- c("age_mean","age_sd","gender","White","South_Asian","total","Obesity","T2DM","HYPERLIPIDAEMIA","HEARTFAILURE","AF","CKD","IHD","MI","carotidarterydisease","ISCHAEMICSTROKE","hyperten","PVD","Livercancer","liver_decomp_cat","GI_cancer","nonGI_cancer","all_cancer","deathind")

NASHvsCtrl_age <- t.test(age ~ NASHvsCtrl, data = df_clean)

NASHvsCtrl_female <- chisq.test(x = table(df_clean$NASHvsCtrl, df_clean$female))

NASHvsCtrl_White_count <- chisq.test(x = table(df_clean$NASHvsCtrl, df_clean$White_count))

NASHvsCtrl_Asian_count <- chisq.test(x = table(df_clean$NASHvsCtrl, df_clean$Asian_count))

NASHvsCtrl_Obesity <- chisq.test(x = table(df_clean$NASHvsCtrl, df_clean$Obesity))

NASHvsCtrl_T2DM <- chisq.test(x = table(df_clean$NASHvsCtrl, df_clean$T2DM))

NASHvsCtrl_HYPERLIPIDAEMIA <- chisq.test(x = table(df_clean$NASHvsCtrl, df_clean$HYPERLIPIDAEMIA))

NASHvsCtrl_HEARTFAILURE <- chisq.test(x = table(df_clean$NASHvsCtrl, df_clean$HEARTFAILURE))

NASHvsCtrl_AF <- chisq.test(x = table(df_clean$NASHvsCtrl, df_clean$AF))

NASHvsCtrl_CKD <- chisq.test(x = table(df_clean$NASHvsCtrl, df_clean$CKD))

NASHvsCtrl_IHD <- chisq.test(x = table(df_clean$NASHvsCtrl, df_clean$IHD))

NASHvsCtrl_MI <- chisq.test(x = table(df_clean$NASHvsCtrl, df_clean$MI))

NASHvsCtrl_carotidarterydisease <- chisq.test(x = table(df_clean$NASHvsCtrl, df_clean$carotidarterydisease))

NASHvsCtrl_ISCHAEMICSTROKE <- chisq.test(x = table(df_clean$NASHvsCtrl, df_clean$ISCHAEMICSTROKE))

NASHvsCtrl_hyperten <- chisq.test(x = table(df_clean$NASHvsCtrl, df_clean$hyperten))

NASHvsCtrl_PVD <- chisq.test(x = table(df_clean$NASHvsCtrl, df_clean$PVD))

NASHvsCtrl_Livercancer <- chisq.test(x = table(df_clean$NASHvsCtrl, df_clean$Livercancer))

NASHvsCtrl_liver_decomp_cat <- chisq.test(x = table(df_clean$NASHvsCtrl, df_clean$liver_decomp_cat))

NASHvsCtrl_GI_cancer <- chisq.test(x = table(df_clean$NASHvsCtrl, df_clean$GI_cancer))

NASHvsCtrl_nonGI_cancer <- chisq.test(x = table(df_clean$NASHvsCtrl, df_clean$nonGI_cancer))

NASHvsCtrl_all_cancer <- chisq.test(x = table(df_clean$NASHvsCtrl, df_clean$all_cancer))

NASHvsCtrl_deathind <- chisq.test(x = table(df_clean$NASHvsCtrl, df_clean$deathind))

NASHvsCtrl_pval <- data.frame(c(NASHvsCtrl_age$p.value, NA, NASHvsCtrl_female$p.value, NASHvsCtrl_White_count$p.value, NASHvsCtrl_Asian_count$p.value, "1", NASHvsCtrl_Obesity$p.value, NASHvsCtrl_T2DM$p.value, NASHvsCtrl_HYPERLIPIDAEMIA$p.value, NASHvsCtrl_HEARTFAILURE$p.value, NASHvsCtrl_AF$p.value, NASHvsCtrl_CKD$p.value, NASHvsCtrl_IHD$p.value, NASHvsCtrl_MI$p.value, NASHvsCtrl_carotidarterydisease$p.value, NASHvsCtrl_ISCHAEMICSTROKE$p.value, NASHvsCtrl_hyperten$p.value, NASHvsCtrl_PVD$p.value, NASHvsCtrl_Livercancer$p.value, NASHvsCtrl_liver_decomp_cat$p.value, NASHvsCtrl_GI_cancer$p.value, NASHvsCtrl_nonGI_cancer$p.value, NASHvsCtrl_all_cancer$p.value, NASHvsCtrl_deathind$p.value))

NASHvsCtrl_pval <- NASHvsCtrl_pval %>% rename(NASHvsCtrl_pval = c(1))

NASHvsCtrl_pval$var <- c("age_mean","age_sd","gender","White","South_Asian","total","Obesity","T2DM","HYPERLIPIDAEMIA","HEARTFAILURE","AF","CKD","IHD","MI","carotidarterydisease","ISCHAEMICSTROKE","hyperten","PVD","Livercancer","liver_decomp_cat","GI_cancer","nonGI_cancer","all_cancer","deathind")

NAFLvsNASH_age <- t.test(age ~ NAFLvsNASH, data = df_clean)

NAFLvsNASH_female <- chisq.test(x = table(df_clean$NAFLvsNASH, df_clean$female))

NAFLvsNASH_White_count <- chisq.test(x = table(df_clean$NAFLvsNASH, df_clean$White_count))

NAFLvsNASH_Asian_count <- chisq.test(x = table(df_clean$NAFLvsNASH, df_clean$Asian_count))

NAFLvsNASH_Obesity <- chisq.test(x = table(df_clean$NAFLvsNASH, df_clean$Obesity))

NAFLvsNASH_T2DM <- chisq.test(x = table(df_clean$NAFLvsNASH, df_clean$T2DM))

NAFLvsNASH_HYPERLIPIDAEMIA <- chisq.test(x = table(df_clean$NAFLvsNASH, df_clean$HYPERLIPIDAEMIA))

NAFLvsNASH_HEARTFAILURE <- chisq.test(x = table(df_clean$NAFLvsNASH, df_clean$HEARTFAILURE))

NAFLvsNASH_AF <- chisq.test(x = table(df_clean$NAFLvsNASH, df_clean$AF))

NAFLvsNASH_CKD <- chisq.test(x = table(df_clean$NAFLvsNASH, df_clean$CKD))

NAFLvsNASH_IHD <- chisq.test(x = table(df_clean$NAFLvsNASH, df_clean$IHD))

NAFLvsNASH_MI <- chisq.test(x = table(df_clean$NAFLvsNASH, df_clean$MI))

NAFLvsNASH_carotidarterydisease <- chisq.test(x = table(df_clean$NAFLvsNASH, df_clean$carotidarterydisease))

NAFLvsNASH_ISCHAEMICSTROKE <- chisq.test(x = table(df_clean$NAFLvsNASH, df_clean$ISCHAEMICSTROKE))

NAFLvsNASH_hyperten <- chisq.test(x = table(df_clean$NAFLvsNASH, df_clean$hyperten))

NAFLvsNASH_PVD <- chisq.test(x = table(df_clean$NAFLvsNASH, df_clean$PVD))

NAFLvsNASH_Livercancer <- chisq.test(x = table(df_clean$NAFLvsNASH, df_clean$Livercancer))

NAFLvsNASH_liver_decomp_cat <- chisq.test(x = table(df_clean$NAFLvsNASH, df_clean$liver_decomp_cat))

NAFLvsNASH_GI_cancer <- chisq.test(x = table(df_clean$NAFLvsNASH, df_clean$GI_cancer))

NAFLvsNASH_nonGI_cancer <- chisq.test(x = table(df_clean$NAFLvsNASH, df_clean$nonGI_cancer))

NAFLvsNASH_all_cancer <- chisq.test(x = table(df_clean$NAFLvsNASH, df_clean$all_cancer))

NAFLvsNASH_deathind <- chisq.test(x = table(df_clean$NAFLvsNASH, df_clean$deathind))

NAFLvsNASH_pval <- data.frame(c(NAFLvsNASH_age$p.value, NA, NAFLvsNASH_female$p.value, NAFLvsNASH_White_count$p.value, NAFLvsNASH_Asian_count$p.value, "1", NAFLvsNASH_Obesity$p.value, NAFLvsNASH_T2DM$p.value, NAFLvsNASH_HYPERLIPIDAEMIA$p.value, NAFLvsNASH_HEARTFAILURE$p.value, NAFLvsNASH_AF$p.value, NAFLvsNASH_CKD$p.value, NAFLvsNASH_IHD$p.value, NAFLvsNASH_MI$p.value, NAFLvsNASH_carotidarterydisease$p.value, NAFLvsNASH_ISCHAEMICSTROKE$p.value, NAFLvsNASH_hyperten$p.value, NAFLvsNASH_PVD$p.value, NAFLvsNASH_Livercancer$p.value, NAFLvsNASH_liver_decomp_cat$p.value, NAFLvsNASH_GI_cancer$p.value, NAFLvsNASH_nonGI_cancer$p.value, NAFLvsNASH_all_cancer$p.value, NAFLvsNASH_deathind$p.value))

NAFLvsNASH_pval <- NAFLvsNASH_pval %>% rename(NAFLvsNASH_pval = c(1))

NAFLvsNASH_pval$var <- c("age_mean","age_sd","gender","White","South_Asian","total","Obesity","T2DM","HYPERLIPIDAEMIA","HEARTFAILURE","AF","CKD","IHD","MI","carotidarterydisease","ISCHAEMICSTROKE","hyperten","PVD","Livercancer","liver_decomp_cat","GI_cancer","nonGI_cancer","all_cancer","deathind")

CirrvsCtrl_age <- t.test(age ~ CirrvsCtrl, data = df_clean)

CirrvsCtrl_female <- chisq.test(x = table(df_clean$CirrvsCtrl, df_clean$female))

CirrvsCtrl_White_count <- chisq.test(x = table(df_clean$CirrvsCtrl, df_clean$White_count))

CirrvsCtrl_Asian_count <- chisq.test(x = table(df_clean$CirrvsCtrl, df_clean$Asian_count))

CirrvsCtrl_Obesity <- chisq.test(x = table(df_clean$CirrvsCtrl, df_clean$Obesity))

CirrvsCtrl_T2DM <- chisq.test(x = table(df_clean$CirrvsCtrl, df_clean$T2DM))

CirrvsCtrl_HYPERLIPIDAEMIA <- chisq.test(x = table(df_clean$CirrvsCtrl, df_clean$HYPERLIPIDAEMIA))

CirrvsCtrl_HEARTFAILURE <- chisq.test(x = table(df_clean$CirrvsCtrl, df_clean$HEARTFAILURE))

CirrvsCtrl_AF <- chisq.test(x = table(df_clean$CirrvsCtrl, df_clean$AF))

CirrvsCtrl_CKD <- chisq.test(x = table(df_clean$CirrvsCtrl, df_clean$CKD))

CirrvsCtrl_IHD <- chisq.test(x = table(df_clean$CirrvsCtrl, df_clean$IHD))

CirrvsCtrl_MI <- chisq.test(x = table(df_clean$CirrvsCtrl, df_clean$MI))

CirrvsCtrl_carotidarterydisease <- chisq.test(x = table(df_clean$CirrvsCtrl, df_clean$carotidarterydisease))

CirrvsCtrl_ISCHAEMICSTROKE <- chisq.test(x = table(df_clean$CirrvsCtrl, df_clean$ISCHAEMICSTROKE))

CirrvsCtrl_hyperten <- chisq.test(x = table(df_clean$CirrvsCtrl, df_clean$hyperten))

CirrvsCtrl_PVD <- chisq.test(x = table(df_clean$CirrvsCtrl, df_clean$PVD))

CirrvsCtrl_Livercancer <- chisq.test(x = table(df_clean$CirrvsCtrl, df_clean$Livercancer))

CirrvsCtrl_liver_decomp_cat <- chisq.test(x = table(df_clean$CirrvsCtrl, df_clean$liver_decomp_cat))

CirrvsCtrl_GI_cancer <- chisq.test(x = table(df_clean$CirrvsCtrl, df_clean$GI_cancer))

CirrvsCtrl_nonGI_cancer <- chisq.test(x = table(df_clean$CirrvsCtrl, df_clean$nonGI_cancer))

CirrvsCtrl_all_cancer <- chisq.test(x = table(df_clean$CirrvsCtrl, df_clean$all_cancer))

CirrvsCtrl_deathind <- chisq.test(x = table(df_clean$CirrvsCtrl, df_clean$deathind))

CirrvsCtrl_pval <- data.frame(c(CirrvsCtrl_age$p.value, NA, CirrvsCtrl_female$p.value, CirrvsCtrl_White_count$p.value, CirrvsCtrl_Asian_count$p.value, "1", CirrvsCtrl_Obesity$p.value, CirrvsCtrl_T2DM$p.value, CirrvsCtrl_HYPERLIPIDAEMIA$p.value, CirrvsCtrl_HEARTFAILURE$p.value, CirrvsCtrl_AF$p.value, CirrvsCtrl_CKD$p.value, CirrvsCtrl_IHD$p.value, CirrvsCtrl_MI$p.value, CirrvsCtrl_carotidarterydisease$p.value, CirrvsCtrl_ISCHAEMICSTROKE$p.value, CirrvsCtrl_hyperten$p.value, CirrvsCtrl_PVD$p.value, CirrvsCtrl_Livercancer$p.value, CirrvsCtrl_liver_decomp_cat$p.value, CirrvsCtrl_GI_cancer$p.value, CirrvsCtrl_nonGI_cancer$p.value, CirrvsCtrl_all_cancer$p.value, CirrvsCtrl_deathind$p.value))

CirrvsCtrl_pval <- CirrvsCtrl_pval %>% rename(CirrvsCtrl_pval = c(1))

CirrvsCtrl_pval$var <- c("age_mean","age_sd","gender","White","South_Asian","total","Obesity","T2DM","HYPERLIPIDAEMIA","HEARTFAILURE","AF","CKD","IHD","MI","carotidarterydisease","ISCHAEMICSTROKE","hyperten","PVD","Livercancer","liver_decomp_cat","GI_cancer","nonGI_cancer","all_cancer","deathind")

CirrvsNAFL_age <- t.test(age ~ CirrvsNAFL, data = df_clean)

CirrvsNAFL_female <- chisq.test(x = table(df_clean$CirrvsNAFL, df_clean$female))

CirrvsNAFL_White_count <- chisq.test(x = table(df_clean$CirrvsNAFL, df_clean$White_count))

CirrvsNAFL_Asian_count <- chisq.test(x = table(df_clean$CirrvsNAFL, df_clean$Asian_count))

CirrvsNAFL_Obesity <- chisq.test(x = table(df_clean$CirrvsNAFL, df_clean$Obesity))

CirrvsNAFL_T2DM <- chisq.test(x = table(df_clean$CirrvsNAFL, df_clean$T2DM))

CirrvsNAFL_HYPERLIPIDAEMIA <- chisq.test(x = table(df_clean$CirrvsNAFL, df_clean$HYPERLIPIDAEMIA))

CirrvsNAFL_HEARTFAILURE <- chisq.test(x = table(df_clean$CirrvsNAFL, df_clean$HEARTFAILURE))

CirrvsNAFL_AF <- chisq.test(x = table(df_clean$CirrvsNAFL, df_clean$AF))

CirrvsNAFL_CKD <- chisq.test(x = table(df_clean$CirrvsNAFL, df_clean$CKD))

CirrvsNAFL_IHD <- chisq.test(x = table(df_clean$CirrvsNAFL, df_clean$IHD))

CirrvsNAFL_MI <- chisq.test(x = table(df_clean$CirrvsNAFL, df_clean$MI))

CirrvsNAFL_carotidarterydisease <- chisq.test(x = table(df_clean$CirrvsNAFL, df_clean$carotidarterydisease))

CirrvsNAFL_ISCHAEMICSTROKE <- chisq.test(x = table(df_clean$CirrvsNAFL, df_clean$ISCHAEMICSTROKE))

CirrvsNAFL_hyperten <- chisq.test(x = table(df_clean$CirrvsNAFL, df_clean$hyperten))

CirrvsNAFL_PVD <- chisq.test(x = table(df_clean$CirrvsNAFL, df_clean$PVD))

CirrvsNAFL_Livercancer <- chisq.test(x = table(df_clean$CirrvsNAFL, df_clean$Livercancer))

CirrvsNAFL_liver_decomp_cat <- chisq.test(x = table(df_clean$CirrvsNAFL, df_clean$liver_decomp_cat))

CirrvsNAFL_GI_cancer <- chisq.test(x = table(df_clean$CirrvsNAFL, df_clean$GI_cancer))

CirrvsNAFL_nonGI_cancer <- chisq.test(x = table(df_clean$CirrvsNAFL, df_clean$nonGI_cancer))

CirrvsNAFL_all_cancer <- chisq.test(x = table(df_clean$CirrvsNAFL, df_clean$all_cancer))

CirrvsNAFL_deathind <- chisq.test(x = table(df_clean$CirrvsNAFL, df_clean$deathind))

CirrvsNAFL_pval <- data.frame(c(CirrvsNAFL_age$p.value, NA, CirrvsNAFL_female$p.value, CirrvsNAFL_White_count$p.value, CirrvsNAFL_Asian_count$p.value, "1", CirrvsNAFL_Obesity$p.value, CirrvsNAFL_T2DM$p.value, CirrvsNAFL_HYPERLIPIDAEMIA$p.value, CirrvsNAFL_HEARTFAILURE$p.value, CirrvsNAFL_AF$p.value, CirrvsNAFL_CKD$p.value, CirrvsNAFL_IHD$p.value, CirrvsNAFL_MI$p.value, CirrvsNAFL_carotidarterydisease$p.value, CirrvsNAFL_ISCHAEMICSTROKE$p.value, CirrvsNAFL_hyperten$p.value, CirrvsNAFL_PVD$p.value, CirrvsNAFL_Livercancer$p.value, CirrvsNAFL_liver_decomp_cat$p.value, CirrvsNAFL_GI_cancer$p.value, CirrvsNAFL_nonGI_cancer$p.value, CirrvsNAFL_all_cancer$p.value, CirrvsNAFL_deathind$p.value))

CirrvsNAFL_pval <- CirrvsNAFL_pval %>% rename(CirrvsNAFL_pval = c(1))

CirrvsNAFL_pval$var <- c("age_mean","age_sd","gender","White","South_Asian","total","Obesity","T2DM","HYPERLIPIDAEMIA","HEARTFAILURE","AF","CKD","IHD","MI","carotidarterydisease","ISCHAEMICSTROKE","hyperten","PVD","Livercancer","liver_decomp_cat","GI_cancer","nonGI_cancer","all_cancer","deathind")

CirrvsNASH_age <- t.test(age ~ CirrvsNASH, data = df_clean)

CirrvsNASH_female <- chisq.test(x = table(df_clean$CirrvsNASH, df_clean$female))

CirrvsNASH_White_count <- chisq.test(x = table(df_clean$CirrvsNASH, df_clean$White_count))

CirrvsNASH_Asian_count <- chisq.test(x = table(df_clean$CirrvsNASH, df_clean$Asian_count))

CirrvsNASH_Obesity <- chisq.test(x = table(df_clean$CirrvsNASH, df_clean$Obesity))

CirrvsNASH_T2DM <- chisq.test(x = table(df_clean$CirrvsNASH, df_clean$T2DM))

CirrvsNASH_HYPERLIPIDAEMIA <- chisq.test(x = table(df_clean$CirrvsNASH, df_clean$HYPERLIPIDAEMIA))

CirrvsNASH_HEARTFAILURE <- chisq.test(x = table(df_clean$CirrvsNASH, df_clean$HEARTFAILURE))

CirrvsNASH_AF <- chisq.test(x = table(df_clean$CirrvsNASH, df_clean$AF))

CirrvsNASH_CKD <- chisq.test(x = table(df_clean$CirrvsNASH, df_clean$CKD))

CirrvsNASH_IHD <- chisq.test(x = table(df_clean$CirrvsNASH, df_clean$IHD))

CirrvsNASH_MI <- chisq.test(x = table(df_clean$CirrvsNASH, df_clean$MI))

CirrvsNASH_carotidarterydisease <- chisq.test(x = table(df_clean$CirrvsNASH, df_clean$carotidarterydisease))

CirrvsNASH_ISCHAEMICSTROKE <- chisq.test(x = table(df_clean$CirrvsNASH, df_clean$ISCHAEMICSTROKE))

CirrvsNASH_hyperten <- chisq.test(x = table(df_clean$CirrvsNASH, df_clean$hyperten))

CirrvsNASH_PVD <- chisq.test(x = table(df_clean$CirrvsNASH, df_clean$PVD))

CirrvsNASH_Livercancer <- chisq.test(x = table(df_clean$CirrvsNASH, df_clean$Livercancer))

CirrvsNASH_liver_decomp_cat <- chisq.test(x = table(df_clean$CirrvsNASH, df_clean$liver_decomp_cat))

CirrvsNASH_GI_cancer <- chisq.test(x = table(df_clean$CirrvsNASH, df_clean$GI_cancer))

CirrvsNASH_nonGI_cancer <- chisq.test(x = table(df_clean$CirrvsNASH, df_clean$nonGI_cancer))

CirrvsNASH_all_cancer <- chisq.test(x = table(df_clean$CirrvsNASH, df_clean$all_cancer))

CirrvsNASH_deathind <- chisq.test(x = table(df_clean$CirrvsNASH, df_clean$deathind))

CirrvsNASH_pval <- data.frame(c(CirrvsNASH_age$p.value, NA, CirrvsNASH_female$p.value, CirrvsNASH_White_count$p.value, CirrvsNASH_Asian_count$p.value, "1", CirrvsNASH_Obesity$p.value, CirrvsNASH_T2DM$p.value, CirrvsNASH_HYPERLIPIDAEMIA$p.value, CirrvsNASH_HEARTFAILURE$p.value, CirrvsNASH_AF$p.value, CirrvsNASH_CKD$p.value, CirrvsNASH_IHD$p.value, CirrvsNASH_MI$p.value, CirrvsNASH_carotidarterydisease$p.value, CirrvsNASH_ISCHAEMICSTROKE$p.value, CirrvsNASH_hyperten$p.value, CirrvsNASH_PVD$p.value, CirrvsNASH_Livercancer$p.value, CirrvsNASH_liver_decomp_cat$p.value, CirrvsNASH_GI_cancer$p.value, CirrvsNASH_nonGI_cancer$p.value, CirrvsNASH_all_cancer$p.value, CirrvsNASH_deathind$p.value))

CirrvsNASH_pval <- CirrvsNASH_pval %>% rename(CirrvsNASH_pval = c(1))

CirrvsNASH_pval$var <- c("age_mean","age_sd","gender","White","South_Asian","total","Obesity","T2DM","HYPERLIPIDAEMIA","HEARTFAILURE","AF","CKD","IHD","MI","carotidarterydisease","ISCHAEMICSTROKE","hyperten","PVD","Livercancer","liver_decomp_cat","GI_cancer","nonGI_cancer","all_cancer","deathind")

group2_sumtab_v2 <- merge(group2_sumtab_t, NAFLvsCtrl_pval, by="var", all=TRUE)

group2_sumtab_v2 <- merge(group2_sumtab_v2, CirrvsCtrl_pval, by="var", all=TRUE)

group2_sumtab_v2 <- merge(group2_sumtab_v2, CirrvsNAFL_pval, by="var", all=TRUE)

group2_sumtab_v2 <- merge(group2_sumtab_v2, CirrvsNASH_pval, by="var", all=TRUE)

group2_sumtab_v2 <- merge(group2_sumtab_v2, NAFLvsNASH_pval, by="var", all=TRUE)

group2_sumtab_v2 <- merge(group2_sumtab_v2, NASHvsCtrl_pval, by="var", all=TRUE)

group2_sumtab_v2 <- group2_sumtab_v2 %>% drop_na()

group2_sumtab_v2$NAFLvsCtrl_qval <- p.adjust(group2_sumtab_v2$NAFLvsCtrl_pval, method = "BH")

group2_sumtab_v2$CirrvsCtrl_qval <- p.adjust(group2_sumtab_v2$CirrvsCtrl_pval, method = "BH")

group2_sumtab_v2$CirrvsNAFL_qval <- p.adjust(group2_sumtab_v2$CirrvsNAFL_pval, method = "BH")

group2_sumtab_v2$CirrvsNASH_qval <- p.adjust(group2_sumtab_v2$CirrvsNASH_pval, method = "BH")

group2_sumtab_v2$NAFLvsNASH_qval <- p.adjust(group2_sumtab_v2$NAFLvsNASH_pval, method = "BH")

group2_sumtab_v2$NASHvsCtrl_qval <- p.adjust(group2_sumtab_v2$NASHvsCtrl_pval, method = "BH")

group2_sumtab_v2$NAFLvsCtrl_qval <- ifelse(group2_sumtab_v2$NAFLvsCtrl_qval <.01, format(group2_sumtab_v2$NAFLvsCtrl_qval, scientific=T, digits=2), format(round(group2_sumtab_v2$NAFLvsCtrl_qval, 2), nsmall = 2))

group2_sumtab_v2$CirrvsCtrl_qval <- ifelse(group2_sumtab_v2$CirrvsCtrl_qval <.01, format(group2_sumtab_v2$CirrvsCtrl_qval, scientific=T, digits=2), format(round(group2_sumtab_v2$CirrvsCtrl_qval, 2), nsmall = 2))

group2_sumtab_v2$CirrvsNAFL_qval <- ifelse(group2_sumtab_v2$CirrvsNAFL_qval <.01, format(group2_sumtab_v2$CirrvsNAFL_qval, scientific=T, digits=2), format(round(group2_sumtab_v2$CirrvsNAFL_qval, 2), nsmall = 2))

group2_sumtab_v2$CirrvsNASH_qval <- ifelse(group2_sumtab_v2$CirrvsNASH_qval <.01, format(group2_sumtab_v2$CirrvsNASH_qval, scientific=T, digits=2), format(round(group2_sumtab_v2$CirrvsNASH_qval, 2), nsmall = 2))

group2_sumtab_v2$NAFLvsNASH_qval <- ifelse(group2_sumtab_v2$NAFLvsNASH_qval <.01, format(group2_sumtab_v2$NAFLvsNASH_qval, scientific=T, digits=2), format(round(group2_sumtab_v2$NAFLvsNASH_qval, 2), nsmall = 2))

group2_sumtab_v2$NASHvsCtrl_qval <- ifelse(group2_sumtab_v2$NASHvsCtrl_qval <.01, format(group2_sumtab_v2$NASHvsCtrl_qval, scientific=T, digits=2), format(round(group2_sumtab_v2$NASHvsCtrl_qval, 2), nsmall = 2))

write.table(group2_sumtab_v2, file="group2_sumtab_v2.csv", sep=",")

#### make adj OR & HR tables for NAFL & NASH separately (group2)

NAFLvsCtrl_liver_decomp_cat_OR <- glm(df_clean$NAFLvsCtrl ~ df_clean$liver_decomp_cat + df_clean$age + df_clean$gender, family = binomial, data = df_clean)

NAFLvsCtrl_liver_decomp_cat_OR_tab <- as.data.frame(confint(NAFLvsCtrl_liver_decomp_cat_OR))

NAFLvsCtrl_liver_decomp_cat_OR_tab$OR <- summary(NAFLvsCtrl_liver_decomp_cat_OR)$coefficients[,1]

NAFLvsCtrl_liver_decomp_cat_OR_tab$z_score <- summary(NAFLvsCtrl_liver_decomp_cat_OR)$coefficients[,3]

NAFLvsCtrl_liver_decomp_cat_OR_tab$NAFLvsCtrl_pval <- 2*pnorm(-abs(NAFLvsCtrl_liver_decomp_cat_OR_tab$z_score))

NAFLvsCtrl_liver_decomp_cat_OR_tab <- NAFLvsCtrl_liver_decomp_cat_OR_tab %>% rename(lower = c(1))

NAFLvsCtrl_liver_decomp_cat_OR_tab <- NAFLvsCtrl_liver_decomp_cat_OR_tab %>% rename(upper = c(2))

NAFLvsCtrl_liver_decomp_cat_OR_tab$NAFLvsCtrl_text <- paste(format(round(NAFLvsCtrl_liver_decomp_cat_OR_tab$OR, 1), nsmall = 1), format(round(NAFLvsCtrl_liver_decomp_cat_OR_tab$lower, 1), nsmall = 1), sep = ' (', collapse = NULL)

NAFLvsCtrl_liver_decomp_cat_OR_tab$NAFLvsCtrl_text <- paste(NAFLvsCtrl_liver_decomp_cat_OR_tab$NAFLvsCtrl_text, format(round(NAFLvsCtrl_liver_decomp_cat_OR_tab$upper, 1), nsmall = 1), sep = '-', collapse = NULL)

NAFLvsCtrl_liver_decomp_cat_OR_tab$NAFLvsCtrl_text <- paste(NAFLvsCtrl_liver_decomp_cat_OR_tab$NAFLvsCtrl_text, '', sep = ')', collapse = NULL)

NAFLvsCtrl_liver_decomp_cat_OR_tab$var <- "liver_decomp_cat"

NAFLvsCtrl_liver_decomp_cat_OR_tab <- NAFLvsCtrl_liver_decomp_cat_OR_tab %>% slice(2)

NAFLvsCtrl_liver_decomp_cat_OR_tab <- NAFLvsCtrl_liver_decomp_cat_OR_tab %>% select(5:7)

NAFLvsCtrl_Livercancer_OR <- glm(df_clean$NAFLvsCtrl ~ df_clean$Livercancer + df_clean$age + df_clean$gender, family = binomial, data = df_clean)

NAFLvsCtrl_Livercancer_OR_tab <- as.data.frame(confint(NAFLvsCtrl_Livercancer_OR))

NAFLvsCtrl_Livercancer_OR_tab$OR <- summary(NAFLvsCtrl_Livercancer_OR)$coefficients[,1]

NAFLvsCtrl_Livercancer_OR_tab$z_score <- summary(NAFLvsCtrl_Livercancer_OR)$coefficients[,3]

NAFLvsCtrl_Livercancer_OR_tab$NAFLvsCtrl_pval <- 2*pnorm(-abs(NAFLvsCtrl_Livercancer_OR_tab$z_score))

NAFLvsCtrl_Livercancer_OR_tab <- NAFLvsCtrl_Livercancer_OR_tab %>% rename(lower = c(1))

NAFLvsCtrl_Livercancer_OR_tab <- NAFLvsCtrl_Livercancer_OR_tab %>% rename(upper = c(2))

NAFLvsCtrl_Livercancer_OR_tab$NAFLvsCtrl_text <- paste(format(round(NAFLvsCtrl_Livercancer_OR_tab$OR, 1), nsmall = 1), format(round(NAFLvsCtrl_Livercancer_OR_tab$lower, 1), nsmall = 1), sep = ' (', collapse = NULL)

NAFLvsCtrl_Livercancer_OR_tab$NAFLvsCtrl_text <- paste(NAFLvsCtrl_Livercancer_OR_tab$NAFLvsCtrl_text, format(round(NAFLvsCtrl_Livercancer_OR_tab$upper, 1), nsmall = 1), sep = '-', collapse = NULL)

NAFLvsCtrl_Livercancer_OR_tab$NAFLvsCtrl_text <- paste(NAFLvsCtrl_Livercancer_OR_tab$NAFLvsCtrl_text, '', sep = ')', collapse = NULL)

NAFLvsCtrl_Livercancer_OR_tab$var <- "Livercancer"

NAFLvsCtrl_Livercancer_OR_tab <- NAFLvsCtrl_Livercancer_OR_tab %>% slice(2)

NAFLvsCtrl_Livercancer_OR_tab <- NAFLvsCtrl_Livercancer_OR_tab %>% select(5:7)

NAFLvsCtrl_mort_demog <- coxph(Surv(mortalit, deathind) ~ NAFLvsCtrl + age + gender, data = df_clean)

NAFLvsCtrl_mort_demog_tab <- as.data.frame(confint(NAFLvsCtrl_mort_demog))

NAFLvsCtrl_mort_demog_tab$HR <- coef(NAFLvsCtrl_mort_demog)

NAFLvsCtrl_mort_demog_tab <- NAFLvsCtrl_mort_demog_tab %>% rename(lower = c(1))

NAFLvsCtrl_mort_demog_tab <- NAFLvsCtrl_mort_demog_tab %>% rename(upper = c(2))

NAFLvsCtrl_mort_demog_tab$HR <- exp(NAFLvsCtrl_mort_demog_tab$HR)

NAFLvsCtrl_mort_demog_tab$lower <- exp(NAFLvsCtrl_mort_demog_tab$lower)

NAFLvsCtrl_mort_demog_tab$upper <- exp(NAFLvsCtrl_mort_demog_tab$upper)

NAFLvsCtrl_mort_demog_tab$z_score <- summary(NAFLvsCtrl_mort_demog)$coefficients[,4]

NAFLvsCtrl_mort_demog_tab$NAFLvsCtrl_pval <- 2*pnorm(-abs(NAFLvsCtrl_mort_demog_tab$z_score))

NAFLvsCtrl_mort_demog_tab$NAFLvsCtrl_text <- paste(format(round(NAFLvsCtrl_mort_demog_tab$HR, 1), nsmall = 1), format(round(NAFLvsCtrl_mort_demog_tab$lower, 1), nsmall = 1), sep = ' (', collapse = NULL)

NAFLvsCtrl_mort_demog_tab$NAFLvsCtrl_text <- paste(NAFLvsCtrl_mort_demog_tab$NAFLvsCtrl_text, format(round(NAFLvsCtrl_mort_demog_tab$upper, 1), nsmall = 1), sep = '-', collapse = NULL)

NAFLvsCtrl_mort_demog_tab$NAFLvsCtrl_text <- paste(NAFLvsCtrl_mort_demog_tab$NAFLvsCtrl_text, '', sep = ')', collapse = NULL)

NAFLvsCtrl_mort_demog_tab$var <- "mort_demog"

NAFLvsCtrl_mort_demog_tab <- NAFLvsCtrl_mort_demog_tab %>% slice(1)

NAFLvsCtrl_mort_demog_tab <- NAFLvsCtrl_mort_demog_tab %>% select(5:7)

NAFLvsCtrl_mort_CVD <- coxph(Surv(mortalit, deathind) ~ NAFLvsCtrl + age + gender + T2DM + Obesity + HYPERLIPIDAEMIA + HEARTFAILURE + AF + CKD + IHD + MI + ISCHAEMICSTROKE + hyperten + PVD, data = df_clean)

NAFLvsCtrl_mort_CVD_tab <- as.data.frame(confint(NAFLvsCtrl_mort_CVD))

NAFLvsCtrl_mort_CVD_tab$HR <- coef(NAFLvsCtrl_mort_CVD)

NAFLvsCtrl_mort_CVD_tab <- NAFLvsCtrl_mort_CVD_tab %>% rename(lower = c(1))

NAFLvsCtrl_mort_CVD_tab <- NAFLvsCtrl_mort_CVD_tab %>% rename(upper = c(2))

NAFLvsCtrl_mort_CVD_tab$HR <- exp(NAFLvsCtrl_mort_CVD_tab$HR)

NAFLvsCtrl_mort_CVD_tab$lower <- exp(NAFLvsCtrl_mort_CVD_tab$lower)

NAFLvsCtrl_mort_CVD_tab$upper <- exp(NAFLvsCtrl_mort_CVD_tab$upper)

NAFLvsCtrl_mort_CVD_tab$z_score <- summary(NAFLvsCtrl_mort_CVD)$coefficients[,4]

NAFLvsCtrl_mort_CVD_tab$NAFLvsCtrl_pval <- 2*pnorm(-abs(NAFLvsCtrl_mort_CVD_tab$z_score))

NAFLvsCtrl_mort_CVD_tab$NAFLvsCtrl_text <- paste(format(round(NAFLvsCtrl_mort_CVD_tab$HR, 1), nsmall = 1), format(round(NAFLvsCtrl_mort_CVD_tab$lower, 1), nsmall = 1), sep = ' (', collapse = NULL)

NAFLvsCtrl_mort_CVD_tab$NAFLvsCtrl_text <- paste(NAFLvsCtrl_mort_CVD_tab$NAFLvsCtrl_text, format(round(NAFLvsCtrl_mort_CVD_tab$upper, 1), nsmall = 1), sep = '-', collapse = NULL)

NAFLvsCtrl_mort_CVD_tab$NAFLvsCtrl_text <- paste(NAFLvsCtrl_mort_CVD_tab$NAFLvsCtrl_text, '', sep = ')', collapse = NULL)

NAFLvsCtrl_mort_CVD_tab$var <- "mort_CVD"

NAFLvsCtrl_mort_CVD_tab <- NAFLvsCtrl_mort_CVD_tab %>% slice(1)

NAFLvsCtrl_mort_CVD_tab <- NAFLvsCtrl_mort_CVD_tab %>% select(5:7)

NAFLvsCtrl_mort_liver <- coxph(Surv(mortalit, deathind) ~ NAFLvsCtrl + age + gender + Livercancer + liver_decomp_cat, data = df_clean)

NAFLvsCtrl_mort_liver_tab <- as.data.frame(confint(NAFLvsCtrl_mort_liver))

NAFLvsCtrl_mort_liver_tab$HR <- coef(NAFLvsCtrl_mort_liver)

NAFLvsCtrl_mort_liver_tab <- NAFLvsCtrl_mort_liver_tab %>% rename(lower = c(1))

NAFLvsCtrl_mort_liver_tab <- NAFLvsCtrl_mort_liver_tab %>% rename(upper = c(2))

NAFLvsCtrl_mort_liver_tab$HR <- exp(NAFLvsCtrl_mort_liver_tab$HR)

NAFLvsCtrl_mort_liver_tab$lower <- exp(NAFLvsCtrl_mort_liver_tab$lower)

NAFLvsCtrl_mort_liver_tab$upper <- exp(NAFLvsCtrl_mort_liver_tab$upper)

NAFLvsCtrl_mort_liver_tab$z_score <- summary(NAFLvsCtrl_mort_liver)$coefficients[,4]

NAFLvsCtrl_mort_liver_tab$NAFLvsCtrl_pval <- 2*pnorm(-abs(NAFLvsCtrl_mort_liver_tab$z_score))

NAFLvsCtrl_mort_liver_tab$NAFLvsCtrl_text <- paste(format(round(NAFLvsCtrl_mort_liver_tab$HR, 1), nsmall = 1), format(round(NAFLvsCtrl_mort_liver_tab$lower, 1), nsmall = 1), sep = ' (', collapse = NULL)

NAFLvsCtrl_mort_liver_tab$NAFLvsCtrl_text <- paste(NAFLvsCtrl_mort_liver_tab$NAFLvsCtrl_text, format(round(NAFLvsCtrl_mort_liver_tab$upper, 1), nsmall = 1), sep = '-', collapse = NULL)

NAFLvsCtrl_mort_liver_tab$NAFLvsCtrl_text <- paste(NAFLvsCtrl_mort_liver_tab$NAFLvsCtrl_text, '', sep = ')', collapse = NULL)

NAFLvsCtrl_mort_liver_tab$var <- "mort_liver"

NAFLvsCtrl_mort_liver_tab <- NAFLvsCtrl_mort_liver_tab %>% slice(1)

NAFLvsCtrl_mort_liver_tab <- NAFLvsCtrl_mort_liver_tab %>% select(5:7)

NAFLvsCtrl_mort_CVDLiv <- coxph(Surv(mortalit, deathind) ~ NAFLvsCtrl + age + gender + T2DM + Obesity + HYPERLIPIDAEMIA + HEARTFAILURE + AF + CKD + IHD + MI + ISCHAEMICSTROKE + hyperten + PVD + Livercancer + liver_decomp_cat, data = df_clean)

NAFLvsCtrl_mort_CVDLiv_tab <- as.data.frame(confint(NAFLvsCtrl_mort_CVDLiv))

NAFLvsCtrl_mort_CVDLiv_tab$HR <- coef(NAFLvsCtrl_mort_CVDLiv)

NAFLvsCtrl_mort_CVDLiv_tab <- NAFLvsCtrl_mort_CVDLiv_tab %>% rename(lower = c(1))

NAFLvsCtrl_mort_CVDLiv_tab <- NAFLvsCtrl_mort_CVDLiv_tab %>% rename(upper = c(2))

NAFLvsCtrl_mort_CVDLiv_tab$HR <- exp(NAFLvsCtrl_mort_CVDLiv_tab$HR)

NAFLvsCtrl_mort_CVDLiv_tab$lower <- exp(NAFLvsCtrl_mort_CVDLiv_tab$lower)

NAFLvsCtrl_mort_CVDLiv_tab$upper <- exp(NAFLvsCtrl_mort_CVDLiv_tab$upper)

NAFLvsCtrl_mort_CVDLiv_tab$z_score <- summary(NAFLvsCtrl_mort_CVDLiv)$coefficients[,4]

NAFLvsCtrl_mort_CVDLiv_tab$NAFLvsCtrl_pval <- 2*pnorm(-abs(NAFLvsCtrl_mort_CVDLiv_tab$z_score))

NAFLvsCtrl_mort_CVDLiv_tab$NAFLvsCtrl_text <- paste(format(round(NAFLvsCtrl_mort_CVDLiv_tab$HR, 1), nsmall = 1), format(round(NAFLvsCtrl_mort_CVDLiv_tab$lower, 1), nsmall = 1), sep = ' (', collapse = NULL)

NAFLvsCtrl_mort_CVDLiv_tab$NAFLvsCtrl_text <- paste(NAFLvsCtrl_mort_CVDLiv_tab$NAFLvsCtrl_text, format(round(NAFLvsCtrl_mort_CVDLiv_tab$upper, 1), nsmall = 1), sep = '-', collapse = NULL)

NAFLvsCtrl_mort_CVDLiv_tab$NAFLvsCtrl_text <- paste(NAFLvsCtrl_mort_CVDLiv_tab$NAFLvsCtrl_text, '', sep = ')', collapse = NULL)

NAFLvsCtrl_mort_CVDLiv_tab$var <- "mort_CVDLiv"

NAFLvsCtrl_mort_CVDLiv_tab <- NAFLvsCtrl_mort_CVDLiv_tab %>% slice(1)

NAFLvsCtrl_mort_CVDLiv_tab <- NAFLvsCtrl_mort_CVDLiv_tab %>% select(5:7)

NASHvsCtrl_liver_decomp_cat_OR <- glm(df_clean$NASHvsCtrl ~ df_clean$liver_decomp_cat + df_clean$age + df_clean$gender, family = binomial, data = df_clean)

NASHvsCtrl_liver_decomp_cat_OR_tab <- as.data.frame(confint(NASHvsCtrl_liver_decomp_cat_OR))

NASHvsCtrl_liver_decomp_cat_OR_tab$OR <- summary(NASHvsCtrl_liver_decomp_cat_OR)$coefficients[,1]

NASHvsCtrl_liver_decomp_cat_OR_tab$z_score <- summary(NASHvsCtrl_liver_decomp_cat_OR)$coefficients[,3]

NASHvsCtrl_liver_decomp_cat_OR_tab$NASHvsCtrl_pval <- 2*pnorm(-abs(NASHvsCtrl_liver_decomp_cat_OR_tab$z_score))

NASHvsCtrl_liver_decomp_cat_OR_tab <- NASHvsCtrl_liver_decomp_cat_OR_tab %>% rename(lower = c(1))

NASHvsCtrl_liver_decomp_cat_OR_tab <- NASHvsCtrl_liver_decomp_cat_OR_tab %>% rename(upper = c(2))

NASHvsCtrl_liver_decomp_cat_OR_tab$NASHvsCtrl_text <- paste(format(round(NASHvsCtrl_liver_decomp_cat_OR_tab$OR, 1), nsmall = 1), format(round(NASHvsCtrl_liver_decomp_cat_OR_tab$lower, 1), nsmall = 1), sep = ' (', collapse = NULL)

NASHvsCtrl_liver_decomp_cat_OR_tab$NASHvsCtrl_text <- paste(NASHvsCtrl_liver_decomp_cat_OR_tab$NASHvsCtrl_text, format(round(NASHvsCtrl_liver_decomp_cat_OR_tab$upper, 1), nsmall = 1), sep = '-', collapse = NULL)

NASHvsCtrl_liver_decomp_cat_OR_tab$NASHvsCtrl_text <- paste(NASHvsCtrl_liver_decomp_cat_OR_tab$NASHvsCtrl_text, '', sep = ')', collapse = NULL)

NASHvsCtrl_liver_decomp_cat_OR_tab$var <- "liver_decomp_cat"

NASHvsCtrl_liver_decomp_cat_OR_tab <- NASHvsCtrl_liver_decomp_cat_OR_tab %>% slice(2)

NASHvsCtrl_liver_decomp_cat_OR_tab <- NASHvsCtrl_liver_decomp_cat_OR_tab %>% select(5:7)

NASHvsCtrl_Livercancer_OR <- glm(df_clean$NASHvsCtrl ~ df_clean$Livercancer + df_clean$age + df_clean$gender, family = binomial, data = df_clean)

NASHvsCtrl_Livercancer_OR_tab <- as.data.frame(confint(NASHvsCtrl_Livercancer_OR))

NASHvsCtrl_Livercancer_OR_tab$OR <- summary(NASHvsCtrl_Livercancer_OR)$coefficients[,1]

NASHvsCtrl_Livercancer_OR_tab$z_score <- summary(NASHvsCtrl_Livercancer_OR)$coefficients[,3]

NASHvsCtrl_Livercancer_OR_tab$NASHvsCtrl_pval <- 2*pnorm(-abs(NASHvsCtrl_Livercancer_OR_tab$z_score))

NASHvsCtrl_Livercancer_OR_tab <- NASHvsCtrl_Livercancer_OR_tab %>% rename(lower = c(1))

NASHvsCtrl_Livercancer_OR_tab <- NASHvsCtrl_Livercancer_OR_tab %>% rename(upper = c(2))

NASHvsCtrl_Livercancer_OR_tab$NASHvsCtrl_text <- paste(format(round(NASHvsCtrl_Livercancer_OR_tab$OR, 1), nsmall = 1), format(round(NASHvsCtrl_Livercancer_OR_tab$lower, 1), nsmall = 1), sep = ' (', collapse = NULL)

NASHvsCtrl_Livercancer_OR_tab$NASHvsCtrl_text <- paste(NASHvsCtrl_Livercancer_OR_tab$NASHvsCtrl_text, format(round(NASHvsCtrl_Livercancer_OR_tab$upper, 1), nsmall = 1), sep = '-', collapse = NULL)

NASHvsCtrl_Livercancer_OR_tab$NASHvsCtrl_text <- paste(NASHvsCtrl_Livercancer_OR_tab$NASHvsCtrl_text, '', sep = ')', collapse = NULL)

NASHvsCtrl_Livercancer_OR_tab$var <- "Livercancer"

NASHvsCtrl_Livercancer_OR_tab <- NASHvsCtrl_Livercancer_OR_tab %>% slice(2)

NASHvsCtrl_Livercancer_OR_tab <- NASHvsCtrl_Livercancer_OR_tab %>% select(5:7)

NASHvsCtrl_mort_demog <- coxph(Surv(mortalit, deathind) ~ NASHvsCtrl + age + gender, data = df_clean)

NASHvsCtrl_mort_demog_tab <- as.data.frame(confint(NASHvsCtrl_mort_demog))

NASHvsCtrl_mort_demog_tab$HR <- coef(NASHvsCtrl_mort_demog)

NASHvsCtrl_mort_demog_tab <- NASHvsCtrl_mort_demog_tab %>% rename(lower = c(1))

NASHvsCtrl_mort_demog_tab <- NASHvsCtrl_mort_demog_tab %>% rename(upper = c(2))

NASHvsCtrl_mort_demog_tab$HR <- exp(NASHvsCtrl_mort_demog_tab$HR)

NASHvsCtrl_mort_demog_tab$lower <- exp(NASHvsCtrl_mort_demog_tab$lower)

NASHvsCtrl_mort_demog_tab$upper <- exp(NASHvsCtrl_mort_demog_tab$upper)

NASHvsCtrl_mort_demog_tab$z_score <- summary(NASHvsCtrl_mort_demog)$coefficients[,4]

NASHvsCtrl_mort_demog_tab$NASHvsCtrl_pval <- 2*pnorm(-abs(NASHvsCtrl_mort_demog_tab$z_score))

NASHvsCtrl_mort_demog_tab$NASHvsCtrl_text <- paste(format(round(NASHvsCtrl_mort_demog_tab$HR, 1), nsmall = 1), format(round(NASHvsCtrl_mort_demog_tab$lower, 1), nsmall = 1), sep = ' (', collapse = NULL)

NASHvsCtrl_mort_demog_tab$NASHvsCtrl_text <- paste(NASHvsCtrl_mort_demog_tab$NASHvsCtrl_text, format(round(NASHvsCtrl_mort_demog_tab$upper, 1), nsmall = 1), sep = '-', collapse = NULL)

NASHvsCtrl_mort_demog_tab$NASHvsCtrl_text <- paste(NASHvsCtrl_mort_demog_tab$NASHvsCtrl_text, '', sep = ')', collapse = NULL)

NASHvsCtrl_mort_demog_tab$var <- "mort_demog"

NASHvsCtrl_mort_demog_tab <- NASHvsCtrl_mort_demog_tab %>% slice(1)

NASHvsCtrl_mort_demog_tab <- NASHvsCtrl_mort_demog_tab %>% select(5:7)

NASHvsCtrl_mort_CVD <- coxph(Surv(mortalit, deathind) ~ NASHvsCtrl + age + gender + T2DM + Obesity + HYPERLIPIDAEMIA + HEARTFAILURE + AF + CKD + IHD + MI + ISCHAEMICSTROKE + hyperten + PVD, data = df_clean)

NASHvsCtrl_mort_CVD_tab <- as.data.frame(confint(NASHvsCtrl_mort_CVD))

NASHvsCtrl_mort_CVD_tab$HR <- coef(NASHvsCtrl_mort_CVD)

NASHvsCtrl_mort_CVD_tab <- NASHvsCtrl_mort_CVD_tab %>% rename(lower = c(1))

NASHvsCtrl_mort_CVD_tab <- NASHvsCtrl_mort_CVD_tab %>% rename(upper = c(2))

NASHvsCtrl_mort_CVD_tab$HR <- exp(NASHvsCtrl_mort_CVD_tab$HR)

NASHvsCtrl_mort_CVD_tab$lower <- exp(NASHvsCtrl_mort_CVD_tab$lower)

NASHvsCtrl_mort_CVD_tab$upper <- exp(NASHvsCtrl_mort_CVD_tab$upper)

NASHvsCtrl_mort_CVD_tab$z_score <- summary(NASHvsCtrl_mort_CVD)$coefficients[,4]

NASHvsCtrl_mort_CVD_tab$NASHvsCtrl_pval <- 2*pnorm(-abs(NASHvsCtrl_mort_CVD_tab$z_score))

NASHvsCtrl_mort_CVD_tab$NASHvsCtrl_text <- paste(format(round(NASHvsCtrl_mort_CVD_tab$HR, 1), nsmall = 1), format(round(NASHvsCtrl_mort_CVD_tab$lower, 1), nsmall = 1), sep = ' (', collapse = NULL)

NASHvsCtrl_mort_CVD_tab$NASHvsCtrl_text <- paste(NASHvsCtrl_mort_CVD_tab$NASHvsCtrl_text, format(round(NASHvsCtrl_mort_CVD_tab$upper, 1), nsmall = 1), sep = '-', collapse = NULL)

NASHvsCtrl_mort_CVD_tab$NASHvsCtrl_text <- paste(NASHvsCtrl_mort_CVD_tab$NASHvsCtrl_text, '', sep = ')', collapse = NULL)

NASHvsCtrl_mort_CVD_tab$var <- "mort_CVD"

NASHvsCtrl_mort_CVD_tab <- NASHvsCtrl_mort_CVD_tab %>% slice(1)

NASHvsCtrl_mort_CVD_tab <- NASHvsCtrl_mort_CVD_tab %>% select(5:7)

NASHvsCtrl_mort_liver <- coxph(Surv(mortalit, deathind) ~ NASHvsCtrl + age + gender + Livercancer + liver_decomp_cat, data = df_clean)

NASHvsCtrl_mort_liver_tab <- as.data.frame(confint(NASHvsCtrl_mort_liver))

NASHvsCtrl_mort_liver_tab$HR <- coef(NASHvsCtrl_mort_liver)

NASHvsCtrl_mort_liver_tab <- NASHvsCtrl_mort_liver_tab %>% rename(lower = c(1))

NASHvsCtrl_mort_liver_tab <- NASHvsCtrl_mort_liver_tab %>% rename(upper = c(2))

NASHvsCtrl_mort_liver_tab$HR <- exp(NASHvsCtrl_mort_liver_tab$HR)

NASHvsCtrl_mort_liver_tab$lower <- exp(NASHvsCtrl_mort_liver_tab$lower)

NASHvsCtrl_mort_liver_tab$upper <- exp(NASHvsCtrl_mort_liver_tab$upper)

NASHvsCtrl_mort_liver_tab$z_score <- summary(NASHvsCtrl_mort_liver)$coefficients[,4]

NASHvsCtrl_mort_liver_tab$NASHvsCtrl_pval <- 2*pnorm(-abs(NASHvsCtrl_mort_liver_tab$z_score))

NASHvsCtrl_mort_liver_tab$NASHvsCtrl_text <- paste(format(round(NASHvsCtrl_mort_liver_tab$HR, 1), nsmall = 1), format(round(NASHvsCtrl_mort_liver_tab$lower, 1), nsmall = 1), sep = ' (', collapse = NULL)

NASHvsCtrl_mort_liver_tab$NASHvsCtrl_text <- paste(NASHvsCtrl_mort_liver_tab$NASHvsCtrl_text, format(round(NASHvsCtrl_mort_liver_tab$upper, 1), nsmall = 1), sep = '-', collapse = NULL)

NASHvsCtrl_mort_liver_tab$NASHvsCtrl_text <- paste(NASHvsCtrl_mort_liver_tab$NASHvsCtrl_text, '', sep = ')', collapse = NULL)

NASHvsCtrl_mort_liver_tab$var <- "mort_liver"

NASHvsCtrl_mort_liver_tab <- NASHvsCtrl_mort_liver_tab %>% slice(1)

NASHvsCtrl_mort_liver_tab <- NASHvsCtrl_mort_liver_tab %>% select(5:7)

NASHvsCtrl_mort_CVDLiv <- coxph(Surv(mortalit, deathind) ~ NASHvsCtrl + age + gender + T2DM + Obesity + HYPERLIPIDAEMIA + HEARTFAILURE + AF + CKD + IHD + MI + ISCHAEMICSTROKE + hyperten + PVD + Livercancer + liver_decomp_cat, data = df_clean)

NASHvsCtrl_mort_CVDLiv_tab <- as.data.frame(confint(NASHvsCtrl_mort_CVDLiv))

NASHvsCtrl_mort_CVDLiv_tab$HR <- coef(NASHvsCtrl_mort_CVDLiv)

NASHvsCtrl_mort_CVDLiv_tab <- NASHvsCtrl_mort_CVDLiv_tab %>% rename(lower = c(1))

NASHvsCtrl_mort_CVDLiv_tab <- NASHvsCtrl_mort_CVDLiv_tab %>% rename(upper = c(2))

NASHvsCtrl_mort_CVDLiv_tab$HR <- exp(NASHvsCtrl_mort_CVDLiv_tab$HR)

NASHvsCtrl_mort_CVDLiv_tab$lower <- exp(NASHvsCtrl_mort_CVDLiv_tab$lower)

NASHvsCtrl_mort_CVDLiv_tab$upper <- exp(NASHvsCtrl_mort_CVDLiv_tab$upper)

NASHvsCtrl_mort_CVDLiv_tab$z_score <- summary(NASHvsCtrl_mort_CVDLiv)$coefficients[,4]

NASHvsCtrl_mort_CVDLiv_tab$NASHvsCtrl_pval <- 2*pnorm(-abs(NASHvsCtrl_mort_CVDLiv_tab$z_score))

NASHvsCtrl_mort_CVDLiv_tab$NASHvsCtrl_text <- paste(format(round(NASHvsCtrl_mort_CVDLiv_tab$HR, 1), nsmall = 1), format(round(NASHvsCtrl_mort_CVDLiv_tab$lower, 1), nsmall = 1), sep = ' (', collapse = NULL)

NASHvsCtrl_mort_CVDLiv_tab$NASHvsCtrl_text <- paste(NASHvsCtrl_mort_CVDLiv_tab$NASHvsCtrl_text, format(round(NASHvsCtrl_mort_CVDLiv_tab$upper, 1), nsmall = 1), sep = '-', collapse = NULL)

NASHvsCtrl_mort_CVDLiv_tab$NASHvsCtrl_text <- paste(NASHvsCtrl_mort_CVDLiv_tab$NASHvsCtrl_text, '', sep = ')', collapse = NULL)

NASHvsCtrl_mort_CVDLiv_tab$var <- "mort_CVDLiv"

NASHvsCtrl_mort_CVDLiv_tab <- NASHvsCtrl_mort_CVDLiv_tab %>% slice(1)

NASHvsCtrl_mort_CVDLiv_tab <- NASHvsCtrl_mort_CVDLiv_tab %>% select(5:7)

NAFLvsNASH_liver_decomp_cat_OR <- glm(df_clean$NAFLvsNASH ~ df_clean$liver_decomp_cat + df_clean$age + df_clean$gender, family = binomial, data = df_clean)

NAFLvsNASH_liver_decomp_cat_OR_tab <- as.data.frame(confint(NAFLvsNASH_liver_decomp_cat_OR))

NAFLvsNASH_liver_decomp_cat_OR_tab$OR <- summary(NAFLvsNASH_liver_decomp_cat_OR)$coefficients[,1]

NAFLvsNASH_liver_decomp_cat_OR_tab$z_score <- summary(NAFLvsNASH_liver_decomp_cat_OR)$coefficients[,3]

NAFLvsNASH_liver_decomp_cat_OR_tab$NAFLvsNASH_pval <- 2*pnorm(-abs(NAFLvsNASH_liver_decomp_cat_OR_tab$z_score))

NAFLvsNASH_liver_decomp_cat_OR_tab <- NAFLvsNASH_liver_decomp_cat_OR_tab %>% rename(lower = c(1))

NAFLvsNASH_liver_decomp_cat_OR_tab <- NAFLvsNASH_liver_decomp_cat_OR_tab %>% rename(upper = c(2))

NAFLvsNASH_liver_decomp_cat_OR_tab$NAFLvsNASH_text <- paste(format(round(NAFLvsNASH_liver_decomp_cat_OR_tab$OR, 1), nsmall = 1), format(round(NAFLvsNASH_liver_decomp_cat_OR_tab$lower, 1), nsmall = 1), sep = ' (', collapse = NULL)

NAFLvsNASH_liver_decomp_cat_OR_tab$NAFLvsNASH_text <- paste(NAFLvsNASH_liver_decomp_cat_OR_tab$NAFLvsNASH_text, format(round(NAFLvsNASH_liver_decomp_cat_OR_tab$upper, 1), nsmall = 1), sep = '-', collapse = NULL)

NAFLvsNASH_liver_decomp_cat_OR_tab$NAFLvsNASH_text <- paste(NAFLvsNASH_liver_decomp_cat_OR_tab$NAFLvsNASH_text, '', sep = ')', collapse = NULL)

NAFLvsNASH_liver_decomp_cat_OR_tab$var <- "liver_decomp_cat"

NAFLvsNASH_liver_decomp_cat_OR_tab <- NAFLvsNASH_liver_decomp_cat_OR_tab %>% slice(2)

NAFLvsNASH_liver_decomp_cat_OR_tab <- NAFLvsNASH_liver_decomp_cat_OR_tab %>% select(5:7)

NAFLvsNASH_Livercancer_OR <- glm(df_clean$NAFLvsNASH ~ df_clean$Livercancer + df_clean$age + df_clean$gender, family = binomial, data = df_clean)

NAFLvsNASH_Livercancer_OR_tab <- as.data.frame(confint(NAFLvsNASH_Livercancer_OR))

NAFLvsNASH_Livercancer_OR_tab$OR <- summary(NAFLvsNASH_Livercancer_OR)$coefficients[,1]

NAFLvsNASH_Livercancer_OR_tab$z_score <- summary(NAFLvsNASH_Livercancer_OR)$coefficients[,3]

NAFLvsNASH_Livercancer_OR_tab$NAFLvsNASH_pval <- 2*pnorm(-abs(NAFLvsNASH_Livercancer_OR_tab$z_score))

NAFLvsNASH_Livercancer_OR_tab <- NAFLvsNASH_Livercancer_OR_tab %>% rename(lower = c(1))

NAFLvsNASH_Livercancer_OR_tab <- NAFLvsNASH_Livercancer_OR_tab %>% rename(upper = c(2))

NAFLvsNASH_Livercancer_OR_tab$NAFLvsNASH_text <- paste(format(round(NAFLvsNASH_Livercancer_OR_tab$OR, 1), nsmall = 1), format(round(NAFLvsNASH_Livercancer_OR_tab$lower, 1), nsmall = 1), sep = ' (', collapse = NULL)

NAFLvsNASH_Livercancer_OR_tab$NAFLvsNASH_text <- paste(NAFLvsNASH_Livercancer_OR_tab$NAFLvsNASH_text, format(round(NAFLvsNASH_Livercancer_OR_tab$upper, 1), nsmall = 1), sep = '-', collapse = NULL)

NAFLvsNASH_Livercancer_OR_tab$NAFLvsNASH_text <- paste(NAFLvsNASH_Livercancer_OR_tab$NAFLvsNASH_text, '', sep = ')', collapse = NULL)

NAFLvsNASH_Livercancer_OR_tab$var <- "Livercancer"

NAFLvsNASH_Livercancer_OR_tab <- NAFLvsNASH_Livercancer_OR_tab %>% slice(2)

NAFLvsNASH_Livercancer_OR_tab <- NAFLvsNASH_Livercancer_OR_tab %>% select(5:7)

NAFLvsNASH_mort_demog <- coxph(Surv(mortalit, deathind) ~ NAFLvsNASH + age + gender, data = df_clean)

NAFLvsNASH_mort_demog_tab <- as.data.frame(confint(NAFLvsNASH_mort_demog))

NAFLvsNASH_mort_demog_tab$HR <- coef(NAFLvsNASH_mort_demog)

NAFLvsNASH_mort_demog_tab <- NAFLvsNASH_mort_demog_tab %>% rename(lower = c(1))

NAFLvsNASH_mort_demog_tab <- NAFLvsNASH_mort_demog_tab %>% rename(upper = c(2))

NAFLvsNASH_mort_demog_tab$HR <- exp(NAFLvsNASH_mort_demog_tab$HR)

NAFLvsNASH_mort_demog_tab$lower <- exp(NAFLvsNASH_mort_demog_tab$lower)

NAFLvsNASH_mort_demog_tab$upper <- exp(NAFLvsNASH_mort_demog_tab$upper)

NAFLvsNASH_mort_demog_tab$z_score <- summary(NAFLvsNASH_mort_demog)$coefficients[,4]

NAFLvsNASH_mort_demog_tab$NAFLvsNASH_pval <- 2*pnorm(-abs(NAFLvsNASH_mort_demog_tab$z_score))

NAFLvsNASH_mort_demog_tab$NAFLvsNASH_text <- paste(format(round(NAFLvsNASH_mort_demog_tab$HR, 1), nsmall = 1), format(round(NAFLvsNASH_mort_demog_tab$lower, 1), nsmall = 1), sep = ' (', collapse = NULL)

NAFLvsNASH_mort_demog_tab$NAFLvsNASH_text <- paste(NAFLvsNASH_mort_demog_tab$NAFLvsNASH_text, format(round(NAFLvsNASH_mort_demog_tab$upper, 1), nsmall = 1), sep = '-', collapse = NULL)

NAFLvsNASH_mort_demog_tab$NAFLvsNASH_text <- paste(NAFLvsNASH_mort_demog_tab$NAFLvsNASH_text, '', sep = ')', collapse = NULL)

NAFLvsNASH_mort_demog_tab$var <- "mort_demog"

NAFLvsNASH_mort_demog_tab <- NAFLvsNASH_mort_demog_tab %>% slice(1)

NAFLvsNASH_mort_demog_tab <- NAFLvsNASH_mort_demog_tab %>% select(5:7)

NAFLvsNASH_mort_CVD <- coxph(Surv(mortalit, deathind) ~ NAFLvsNASH + age + gender + T2DM + Obesity + HYPERLIPIDAEMIA + HEARTFAILURE + AF + CKD + IHD + MI + ISCHAEMICSTROKE + hyperten + PVD, data = df_clean)

NAFLvsNASH_mort_CVD_tab <- as.data.frame(confint(NAFLvsNASH_mort_CVD))

NAFLvsNASH_mort_CVD_tab$HR <- coef(NAFLvsNASH_mort_CVD)

NAFLvsNASH_mort_CVD_tab <- NAFLvsNASH_mort_CVD_tab %>% rename(lower = c(1))

NAFLvsNASH_mort_CVD_tab <- NAFLvsNASH_mort_CVD_tab %>% rename(upper = c(2))

NAFLvsNASH_mort_CVD_tab$HR <- exp(NAFLvsNASH_mort_CVD_tab$HR)

NAFLvsNASH_mort_CVD_tab$lower <- exp(NAFLvsNASH_mort_CVD_tab$lower)

NAFLvsNASH_mort_CVD_tab$upper <- exp(NAFLvsNASH_mort_CVD_tab$upper)

NAFLvsNASH_mort_CVD_tab$z_score <- summary(NAFLvsNASH_mort_CVD)$coefficients[,4]

NAFLvsNASH_mort_CVD_tab$NAFLvsNASH_pval <- 2*pnorm(-abs(NAFLvsNASH_mort_CVD_tab$z_score))

NAFLvsNASH_mort_CVD_tab$NAFLvsNASH_text <- paste(format(round(NAFLvsNASH_mort_CVD_tab$HR, 1), nsmall = 1), format(round(NAFLvsNASH_mort_CVD_tab$lower, 1), nsmall = 1), sep = ' (', collapse = NULL)

NAFLvsNASH_mort_CVD_tab$NAFLvsNASH_text <- paste(NAFLvsNASH_mort_CVD_tab$NAFLvsNASH_text, format(round(NAFLvsNASH_mort_CVD_tab$upper, 1), nsmall = 1), sep = '-', collapse = NULL)

NAFLvsNASH_mort_CVD_tab$NAFLvsNASH_text <- paste(NAFLvsNASH_mort_CVD_tab$NAFLvsNASH_text, '', sep = ')', collapse = NULL)

NAFLvsNASH_mort_CVD_tab$var <- "mort_CVD"

NAFLvsNASH_mort_CVD_tab <- NAFLvsNASH_mort_CVD_tab %>% slice(1)

NAFLvsNASH_mort_CVD_tab <- NAFLvsNASH_mort_CVD_tab %>% select(5:7)

NAFLvsNASH_mort_liver <- coxph(Surv(mortalit, deathind) ~ NAFLvsNASH + age + gender + Livercancer + liver_decomp_cat, data = df_clean)

NAFLvsNASH_mort_liver_tab <- as.data.frame(confint(NAFLvsNASH_mort_liver))

NAFLvsNASH_mort_liver_tab$HR <- coef(NAFLvsNASH_mort_liver)

NAFLvsNASH_mort_liver_tab <- NAFLvsNASH_mort_liver_tab %>% rename(lower = c(1))

NAFLvsNASH_mort_liver_tab <- NAFLvsNASH_mort_liver_tab %>% rename(upper = c(2))

NAFLvsNASH_mort_liver_tab$HR <- exp(NAFLvsNASH_mort_liver_tab$HR)

NAFLvsNASH_mort_liver_tab$lower <- exp(NAFLvsNASH_mort_liver_tab$lower)

NAFLvsNASH_mort_liver_tab$upper <- exp(NAFLvsNASH_mort_liver_tab$upper)

NAFLvsNASH_mort_liver_tab$z_score <- summary(NAFLvsNASH_mort_liver)$coefficients[,4]

NAFLvsNASH_mort_liver_tab$NAFLvsNASH_pval <- 2*pnorm(-abs(NAFLvsNASH_mort_liver_tab$z_score))

NAFLvsNASH_mort_liver_tab$NAFLvsNASH_text <- paste(format(round(NAFLvsNASH_mort_liver_tab$HR, 1), nsmall = 1), format(round(NAFLvsNASH_mort_liver_tab$lower, 1), nsmall = 1), sep = ' (', collapse = NULL)

NAFLvsNASH_mort_liver_tab$NAFLvsNASH_text <- paste(NAFLvsNASH_mort_liver_tab$NAFLvsNASH_text, format(round(NAFLvsNASH_mort_liver_tab$upper, 1), nsmall = 1), sep = '-', collapse = NULL)

NAFLvsNASH_mort_liver_tab$NAFLvsNASH_text <- paste(NAFLvsNASH_mort_liver_tab$NAFLvsNASH_text, '', sep = ')', collapse = NULL)

NAFLvsNASH_mort_liver_tab$var <- "mort_liver"

NAFLvsNASH_mort_liver_tab <- NAFLvsNASH_mort_liver_tab %>% slice(1)

NAFLvsNASH_mort_liver_tab <- NAFLvsNASH_mort_liver_tab %>% select(5:7)

NAFLvsNASH_mort_CVDLiv <- coxph(Surv(mortalit, deathind) ~ NAFLvsNASH + age + gender + T2DM + Obesity + HYPERLIPIDAEMIA + HEARTFAILURE + AF + CKD + IHD + MI + ISCHAEMICSTROKE + hyperten + PVD + Livercancer + liver_decomp_cat, data = df_clean)

NAFLvsNASH_mort_CVDLiv_tab <- as.data.frame(confint(NAFLvsNASH_mort_CVDLiv))

NAFLvsNASH_mort_CVDLiv_tab$HR <- coef(NAFLvsNASH_mort_CVDLiv)

NAFLvsNASH_mort_CVDLiv_tab <- NAFLvsNASH_mort_CVDLiv_tab %>% rename(lower = c(1))

NAFLvsNASH_mort_CVDLiv_tab <- NAFLvsNASH_mort_CVDLiv_tab %>% rename(upper = c(2))

NAFLvsNASH_mort_CVDLiv_tab$HR <- exp(NAFLvsNASH_mort_CVDLiv_tab$HR)

NAFLvsNASH_mort_CVDLiv_tab$lower <- exp(NAFLvsNASH_mort_CVDLiv_tab$lower)

NAFLvsNASH_mort_CVDLiv_tab$upper <- exp(NAFLvsNASH_mort_CVDLiv_tab$upper)

NAFLvsNASH_mort_CVDLiv_tab$z_score <- summary(NAFLvsNASH_mort_CVDLiv)$coefficients[,4]

NAFLvsNASH_mort_CVDLiv_tab$NAFLvsNASH_pval <- 2*pnorm(-abs(NAFLvsNASH_mort_CVDLiv_tab$z_score))

NAFLvsNASH_mort_CVDLiv_tab$NAFLvsNASH_text <- paste(format(round(NAFLvsNASH_mort_CVDLiv_tab$HR, 1), nsmall = 1), format(round(NAFLvsNASH_mort_CVDLiv_tab$lower, 1), nsmall = 1), sep = ' (', collapse = NULL)

NAFLvsNASH_mort_CVDLiv_tab$NAFLvsNASH_text <- paste(NAFLvsNASH_mort_CVDLiv_tab$NAFLvsNASH_text, format(round(NAFLvsNASH_mort_CVDLiv_tab$upper, 1), nsmall = 1), sep = '-', collapse = NULL)

NAFLvsNASH_mort_CVDLiv_tab$NAFLvsNASH_text <- paste(NAFLvsNASH_mort_CVDLiv_tab$NAFLvsNASH_text, '', sep = ')', collapse = NULL)

NAFLvsNASH_mort_CVDLiv_tab$var <- "mort_CVDLiv"

NAFLvsNASH_mort_CVDLiv_tab <- NAFLvsNASH_mort_CVDLiv_tab %>% slice(1)

NAFLvsNASH_mort_CVDLiv_tab <- NAFLvsNASH_mort_CVDLiv_tab %>% select(5:7)

CirrvsNASH_liver_decomp_cat_OR <- glm(df_clean$CirrvsNASH ~ df_clean$liver_decomp_cat + df_clean$age + df_clean$gender, family = binomial, data = df_clean)

CirrvsNASH_liver_decomp_cat_OR_tab <- as.data.frame(confint(CirrvsNASH_liver_decomp_cat_OR))

CirrvsNASH_liver_decomp_cat_OR_tab$OR <- summary(CirrvsNASH_liver_decomp_cat_OR)$coefficients[,1]

CirrvsNASH_liver_decomp_cat_OR_tab$z_score <- summary(CirrvsNASH_liver_decomp_cat_OR)$coefficients[,3]

CirrvsNASH_liver_decomp_cat_OR_tab$CirrvsNASH_pval <- 2*pnorm(-abs(CirrvsNASH_liver_decomp_cat_OR_tab$z_score))

CirrvsNASH_liver_decomp_cat_OR_tab <- CirrvsNASH_liver_decomp_cat_OR_tab %>% rename(lower = c(1))

CirrvsNASH_liver_decomp_cat_OR_tab <- CirrvsNASH_liver_decomp_cat_OR_tab %>% rename(upper = c(2))

CirrvsNASH_liver_decomp_cat_OR_tab$CirrvsNASH_text <- paste(format(round(CirrvsNASH_liver_decomp_cat_OR_tab$OR, 1), nsmall = 1), format(round(CirrvsNASH_liver_decomp_cat_OR_tab$lower, 1), nsmall = 1), sep = ' (', collapse = NULL)

CirrvsNASH_liver_decomp_cat_OR_tab$CirrvsNASH_text <- paste(CirrvsNASH_liver_decomp_cat_OR_tab$CirrvsNASH_text, format(round(CirrvsNASH_liver_decomp_cat_OR_tab$upper, 1), nsmall = 1), sep = '-', collapse = NULL)

CirrvsNASH_liver_decomp_cat_OR_tab$CirrvsNASH_text <- paste(CirrvsNASH_liver_decomp_cat_OR_tab$CirrvsNASH_text, '', sep = ')', collapse = NULL)

CirrvsNASH_liver_decomp_cat_OR_tab$var <- "liver_decomp_cat"

CirrvsNASH_liver_decomp_cat_OR_tab <- CirrvsNASH_liver_decomp_cat_OR_tab %>% slice(2)

CirrvsNASH_liver_decomp_cat_OR_tab <- CirrvsNASH_liver_decomp_cat_OR_tab %>% select(5:7)

CirrvsNASH_Livercancer_OR <- glm(df_clean$CirrvsNASH ~ df_clean$Livercancer + df_clean$age + df_clean$gender, family = binomial, data = df_clean)

CirrvsNASH_Livercancer_OR_tab <- as.data.frame(confint(CirrvsNASH_Livercancer_OR))

CirrvsNASH_Livercancer_OR_tab$OR <- summary(CirrvsNASH_Livercancer_OR)$coefficients[,1]

CirrvsNASH_Livercancer_OR_tab$z_score <- summary(CirrvsNASH_Livercancer_OR)$coefficients[,3]

CirrvsNASH_Livercancer_OR_tab$CirrvsNASH_pval <- 2*pnorm(-abs(CirrvsNASH_Livercancer_OR_tab$z_score))

CirrvsNASH_Livercancer_OR_tab <- CirrvsNASH_Livercancer_OR_tab %>% rename(lower = c(1))

CirrvsNASH_Livercancer_OR_tab <- CirrvsNASH_Livercancer_OR_tab %>% rename(upper = c(2))

CirrvsNASH_Livercancer_OR_tab$CirrvsNASH_text <- paste(format(round(CirrvsNASH_Livercancer_OR_tab$OR, 1), nsmall = 1), format(round(CirrvsNASH_Livercancer_OR_tab$lower, 1), nsmall = 1), sep = ' (', collapse = NULL)

CirrvsNASH_Livercancer_OR_tab$CirrvsNASH_text <- paste(CirrvsNASH_Livercancer_OR_tab$CirrvsNASH_text, format(round(CirrvsNASH_Livercancer_OR_tab$upper, 1), nsmall = 1), sep = '-', collapse = NULL)

CirrvsNASH_Livercancer_OR_tab$CirrvsNASH_text <- paste(CirrvsNASH_Livercancer_OR_tab$CirrvsNASH_text, '', sep = ')', collapse = NULL)

CirrvsNASH_Livercancer_OR_tab$var <- "Livercancer"

CirrvsNASH_Livercancer_OR_tab <- CirrvsNASH_Livercancer_OR_tab %>% slice(2)

CirrvsNASH_Livercancer_OR_tab <- CirrvsNASH_Livercancer_OR_tab %>% select(5:7)

CirrvsNASH_mort_demog <- coxph(Surv(mortalit, deathind) ~ CirrvsNASH + age + gender, data = df_clean)

CirrvsNASH_mort_demog_tab <- as.data.frame(confint(CirrvsNASH_mort_demog))

CirrvsNASH_mort_demog_tab$HR <- coef(CirrvsNASH_mort_demog)

CirrvsNASH_mort_demog_tab <- CirrvsNASH_mort_demog_tab %>% rename(lower = c(1))

CirrvsNASH_mort_demog_tab <- CirrvsNASH_mort_demog_tab %>% rename(upper = c(2))

CirrvsNASH_mort_demog_tab$HR <- exp(CirrvsNASH_mort_demog_tab$HR)

CirrvsNASH_mort_demog_tab$lower <- exp(CirrvsNASH_mort_demog_tab$lower)

CirrvsNASH_mort_demog_tab$upper <- exp(CirrvsNASH_mort_demog_tab$upper)

CirrvsNASH_mort_demog_tab$z_score <- summary(CirrvsNASH_mort_demog)$coefficients[,4]

CirrvsNASH_mort_demog_tab$CirrvsNASH_pval <- 2*pnorm(-abs(CirrvsNASH_mort_demog_tab$z_score))

CirrvsNASH_mort_demog_tab$CirrvsNASH_text <- paste(format(round(CirrvsNASH_mort_demog_tab$HR, 1), nsmall = 1), format(round(CirrvsNASH_mort_demog_tab$lower, 1), nsmall = 1), sep = ' (', collapse = NULL)

CirrvsNASH_mort_demog_tab$CirrvsNASH_text <- paste(CirrvsNASH_mort_demog_tab$CirrvsNASH_text, format(round(CirrvsNASH_mort_demog_tab$upper, 1), nsmall = 1), sep = '-', collapse = NULL)

CirrvsNASH_mort_demog_tab$CirrvsNASH_text <- paste(CirrvsNASH_mort_demog_tab$CirrvsNASH_text, '', sep = ')', collapse = NULL)

CirrvsNASH_mort_demog_tab$var <- "mort_demog"

CirrvsNASH_mort_demog_tab <- CirrvsNASH_mort_demog_tab %>% slice(1)

CirrvsNASH_mort_demog_tab <- CirrvsNASH_mort_demog_tab %>% select(5:7)

CirrvsNASH_mort_CVD <- coxph(Surv(mortalit, deathind) ~ CirrvsNASH + age + gender + T2DM + Obesity + HYPERLIPIDAEMIA + HEARTFAILURE + AF + CKD + IHD + MI + ISCHAEMICSTROKE + hyperten + PVD, data = df_clean)

CirrvsNASH_mort_CVD_tab <- as.data.frame(confint(CirrvsNASH_mort_CVD))

CirrvsNASH_mort_CVD_tab$HR <- coef(CirrvsNASH_mort_CVD)

CirrvsNASH_mort_CVD_tab <- CirrvsNASH_mort_CVD_tab %>% rename(lower = c(1))

CirrvsNASH_mort_CVD_tab <- CirrvsNASH_mort_CVD_tab %>% rename(upper = c(2))

CirrvsNASH_mort_CVD_tab$HR <- exp(CirrvsNASH_mort_CVD_tab$HR)

CirrvsNASH_mort_CVD_tab$lower <- exp(CirrvsNASH_mort_CVD_tab$lower)

CirrvsNASH_mort_CVD_tab$upper <- exp(CirrvsNASH_mort_CVD_tab$upper)

CirrvsNASH_mort_CVD_tab$z_score <- summary(CirrvsNASH_mort_CVD)$coefficients[,4]

CirrvsNASH_mort_CVD_tab$CirrvsNASH_pval <- 2*pnorm(-abs(CirrvsNASH_mort_CVD_tab$z_score))

CirrvsNASH_mort_CVD_tab$CirrvsNASH_text <- paste(format(round(CirrvsNASH_mort_CVD_tab$HR, 1), nsmall = 1), format(round(CirrvsNASH_mort_CVD_tab$lower, 1), nsmall = 1), sep = ' (', collapse = NULL)

CirrvsNASH_mort_CVD_tab$CirrvsNASH_text <- paste(CirrvsNASH_mort_CVD_tab$CirrvsNASH_text, format(round(CirrvsNASH_mort_CVD_tab$upper, 1), nsmall = 1), sep = '-', collapse = NULL)

CirrvsNASH_mort_CVD_tab$CirrvsNASH_text <- paste(CirrvsNASH_mort_CVD_tab$CirrvsNASH_text, '', sep = ')', collapse = NULL)

CirrvsNASH_mort_CVD_tab$var <- "mort_CVD"

CirrvsNASH_mort_CVD_tab <- CirrvsNASH_mort_CVD_tab %>% slice(1)

CirrvsNASH_mort_CVD_tab <- CirrvsNASH_mort_CVD_tab %>% select(5:7)

CirrvsNASH_mort_liver <- coxph(Surv(mortalit, deathind) ~ CirrvsNASH + age + gender + Livercancer + liver_decomp_cat, data = df_clean)

CirrvsNASH_mort_liver_tab <- as.data.frame(confint(CirrvsNASH_mort_liver))

CirrvsNASH_mort_liver_tab$HR <- coef(CirrvsNASH_mort_liver)

CirrvsNASH_mort_liver_tab <- CirrvsNASH_mort_liver_tab %>% rename(lower = c(1))

CirrvsNASH_mort_liver_tab <- CirrvsNASH_mort_liver_tab %>% rename(upper = c(2))

CirrvsNASH_mort_liver_tab$HR <- exp(CirrvsNASH_mort_liver_tab$HR)

CirrvsNASH_mort_liver_tab$lower <- exp(CirrvsNASH_mort_liver_tab$lower)

CirrvsNASH_mort_liver_tab$upper <- exp(CirrvsNASH_mort_liver_tab$upper)

CirrvsNASH_mort_liver_tab$z_score <- summary(CirrvsNASH_mort_liver)$coefficients[,4]

CirrvsNASH_mort_liver_tab$CirrvsNASH_pval <- 2*pnorm(-abs(CirrvsNASH_mort_liver_tab$z_score))

CirrvsNASH_mort_liver_tab$CirrvsNASH_text <- paste(format(round(CirrvsNASH_mort_liver_tab$HR, 1), nsmall = 1), format(round(CirrvsNASH_mort_liver_tab$lower, 1), nsmall = 1), sep = ' (', collapse = NULL)

CirrvsNASH_mort_liver_tab$CirrvsNASH_text <- paste(CirrvsNASH_mort_liver_tab$CirrvsNASH_text, format(round(CirrvsNASH_mort_liver_tab$upper, 1), nsmall = 1), sep = '-', collapse = NULL)

CirrvsNASH_mort_liver_tab$CirrvsNASH_text <- paste(CirrvsNASH_mort_liver_tab$CirrvsNASH_text, '', sep = ')', collapse = NULL)

CirrvsNASH_mort_liver_tab$var <- "mort_liver"

CirrvsNASH_mort_liver_tab <- CirrvsNASH_mort_liver_tab %>% slice(1)

CirrvsNASH_mort_liver_tab <- CirrvsNASH_mort_liver_tab %>% select(5:7)

CirrvsNASH_mort_CVDLiv <- coxph(Surv(mortalit, deathind) ~ CirrvsNASH + age + gender + T2DM + Obesity + HYPERLIPIDAEMIA + HEARTFAILURE + AF + CKD + IHD + MI + ISCHAEMICSTROKE + hyperten + PVD + Livercancer + liver_decomp_cat, data = df_clean)

CirrvsNASH_mort_CVDLiv_tab <- as.data.frame(confint(CirrvsNASH_mort_CVDLiv))

CirrvsNASH_mort_CVDLiv_tab$HR <- coef(CirrvsNASH_mort_CVDLiv)

CirrvsNASH_mort_CVDLiv_tab <- CirrvsNASH_mort_CVDLiv_tab %>% rename(lower = c(1))

CirrvsNASH_mort_CVDLiv_tab <- CirrvsNASH_mort_CVDLiv_tab %>% rename(upper = c(2))

CirrvsNASH_mort_CVDLiv_tab$HR <- exp(CirrvsNASH_mort_CVDLiv_tab$HR)

CirrvsNASH_mort_CVDLiv_tab$lower <- exp(CirrvsNASH_mort_CVDLiv_tab$lower)

CirrvsNASH_mort_CVDLiv_tab$upper <- exp(CirrvsNASH_mort_CVDLiv_tab$upper)

CirrvsNASH_mort_CVDLiv_tab$z_score <- summary(CirrvsNASH_mort_CVDLiv)$coefficients[,4]

CirrvsNASH_mort_CVDLiv_tab$CirrvsNASH_pval <- 2*pnorm(-abs(CirrvsNASH_mort_CVDLiv_tab$z_score))

CirrvsNASH_mort_CVDLiv_tab$CirrvsNASH_text <- paste(format(round(CirrvsNASH_mort_CVDLiv_tab$HR, 1), nsmall = 1), format(round(CirrvsNASH_mort_CVDLiv_tab$lower, 1), nsmall = 1), sep = ' (', collapse = NULL)

CirrvsNASH_mort_CVDLiv_tab$CirrvsNASH_text <- paste(CirrvsNASH_mort_CVDLiv_tab$CirrvsNASH_text, format(round(CirrvsNASH_mort_CVDLiv_tab$upper, 1), nsmall = 1), sep = '-', collapse = NULL)

CirrvsNASH_mort_CVDLiv_tab$CirrvsNASH_text <- paste(CirrvsNASH_mort_CVDLiv_tab$CirrvsNASH_text, '', sep = ')', collapse = NULL)

CirrvsNASH_mort_CVDLiv_tab$var <- "mort_CVDLiv"

CirrvsNASH_mort_CVDLiv_tab <- CirrvsNASH_mort_CVDLiv_tab %>% slice(1)

CirrvsNASH_mort_CVDLiv_tab <- CirrvsNASH_mort_CVDLiv_tab %>% select(5:7)

CirrvsCtrl_liver_decomp_cat_OR <- glm(df_clean$CirrvsCtrl ~ df_clean$liver_decomp_cat + df_clean$age + df_clean$gender, family = binomial, data = df_clean)

CirrvsCtrl_liver_decomp_cat_OR_tab <- as.data.frame(confint(CirrvsCtrl_liver_decomp_cat_OR))

CirrvsCtrl_liver_decomp_cat_OR_tab$OR <- summary(CirrvsCtrl_liver_decomp_cat_OR)$coefficients[,1]

CirrvsCtrl_liver_decomp_cat_OR_tab$z_score <- summary(CirrvsCtrl_liver_decomp_cat_OR)$coefficients[,3]

CirrvsCtrl_liver_decomp_cat_OR_tab$CirrvsCtrl_pval <- 2*pnorm(-abs(CirrvsCtrl_liver_decomp_cat_OR_tab$z_score))

CirrvsCtrl_liver_decomp_cat_OR_tab <- CirrvsCtrl_liver_decomp_cat_OR_tab %>% rename(lower = c(1))

CirrvsCtrl_liver_decomp_cat_OR_tab <- CirrvsCtrl_liver_decomp_cat_OR_tab %>% rename(upper = c(2))

CirrvsCtrl_liver_decomp_cat_OR_tab$CirrvsCtrl_text <- paste(format(round(CirrvsCtrl_liver_decomp_cat_OR_tab$OR, 1), nsmall = 1), format(round(CirrvsCtrl_liver_decomp_cat_OR_tab$lower, 1), nsmall = 1), sep = ' (', collapse = NULL)

CirrvsCtrl_liver_decomp_cat_OR_tab$CirrvsCtrl_text <- paste(CirrvsCtrl_liver_decomp_cat_OR_tab$CirrvsCtrl_text, format(round(CirrvsCtrl_liver_decomp_cat_OR_tab$upper, 1), nsmall = 1), sep = '-', collapse = NULL)

CirrvsCtrl_liver_decomp_cat_OR_tab$CirrvsCtrl_text <- paste(CirrvsCtrl_liver_decomp_cat_OR_tab$CirrvsCtrl_text, '', sep = ')', collapse = NULL)

CirrvsCtrl_liver_decomp_cat_OR_tab$var <- "liver_decomp_cat"

CirrvsCtrl_liver_decomp_cat_OR_tab <- CirrvsCtrl_liver_decomp_cat_OR_tab %>% slice(2)

CirrvsCtrl_liver_decomp_cat_OR_tab <- CirrvsCtrl_liver_decomp_cat_OR_tab %>% select(5:7)

CirrvsCtrl_Livercancer_OR <- glm(df_clean$CirrvsCtrl ~ df_clean$Livercancer + df_clean$age + df_clean$gender, family = binomial, data = df_clean)

CirrvsCtrl_Livercancer_OR_tab <- as.data.frame(confint(CirrvsCtrl_Livercancer_OR))

CirrvsCtrl_Livercancer_OR_tab$OR <- summary(CirrvsCtrl_Livercancer_OR)$coefficients[,1]

CirrvsCtrl_Livercancer_OR_tab$z_score <- summary(CirrvsCtrl_Livercancer_OR)$coefficients[,3]

CirrvsCtrl_Livercancer_OR_tab$CirrvsCtrl_pval <- 2*pnorm(-abs(CirrvsCtrl_Livercancer_OR_tab$z_score))

CirrvsCtrl_Livercancer_OR_tab <- CirrvsCtrl_Livercancer_OR_tab %>% rename(lower = c(1))

CirrvsCtrl_Livercancer_OR_tab <- CirrvsCtrl_Livercancer_OR_tab %>% rename(upper = c(2))

CirrvsCtrl_Livercancer_OR_tab$CirrvsCtrl_text <- paste(format(round(CirrvsCtrl_Livercancer_OR_tab$OR, 1), nsmall = 1), format(round(CirrvsCtrl_Livercancer_OR_tab$lower, 1), nsmall = 1), sep = ' (', collapse = NULL)

CirrvsCtrl_Livercancer_OR_tab$CirrvsCtrl_text <- paste(CirrvsCtrl_Livercancer_OR_tab$CirrvsCtrl_text, format(round(CirrvsCtrl_Livercancer_OR_tab$upper, 1), nsmall = 1), sep = '-', collapse = NULL)

CirrvsCtrl_Livercancer_OR_tab$CirrvsCtrl_text <- paste(CirrvsCtrl_Livercancer_OR_tab$CirrvsCtrl_text, '', sep = ')', collapse = NULL)

CirrvsCtrl_Livercancer_OR_tab$var <- "Livercancer"

CirrvsCtrl_Livercancer_OR_tab <- CirrvsCtrl_Livercancer_OR_tab %>% slice(2)

CirrvsCtrl_Livercancer_OR_tab <- CirrvsCtrl_Livercancer_OR_tab %>% select(5:7)

CirrvsNAFL_liver_decomp_cat_OR <- glm(df_clean$CirrvsNAFL ~ df_clean$liver_decomp_cat + df_clean$age + df_clean$gender, family = binomial, data = df_clean)

CirrvsNAFL_liver_decomp_cat_OR_tab <- as.data.frame(confint(CirrvsNAFL_liver_decomp_cat_OR))

CirrvsNAFL_liver_decomp_cat_OR_tab$OR <- summary(CirrvsNAFL_liver_decomp_cat_OR)$coefficients[,1]

CirrvsNAFL_liver_decomp_cat_OR_tab$z_score <- summary(CirrvsNAFL_liver_decomp_cat_OR)$coefficients[,3]

CirrvsNAFL_liver_decomp_cat_OR_tab$CirrvsNAFL_pval <- 2*pnorm(-abs(CirrvsNAFL_liver_decomp_cat_OR_tab$z_score))

CirrvsNAFL_liver_decomp_cat_OR_tab <- CirrvsNAFL_liver_decomp_cat_OR_tab %>% rename(lower = c(1))

CirrvsNAFL_liver_decomp_cat_OR_tab <- CirrvsNAFL_liver_decomp_cat_OR_tab %>% rename(upper = c(2))

CirrvsNAFL_liver_decomp_cat_OR_tab$CirrvsNAFL_text <- paste(format(round(CirrvsNAFL_liver_decomp_cat_OR_tab$OR, 1), nsmall = 1), format(round(CirrvsNAFL_liver_decomp_cat_OR_tab$lower, 1), nsmall = 1), sep = ' (', collapse = NULL)

CirrvsNAFL_liver_decomp_cat_OR_tab$CirrvsNAFL_text <- paste(CirrvsNAFL_liver_decomp_cat_OR_tab$CirrvsNAFL_text, format(round(CirrvsNAFL_liver_decomp_cat_OR_tab$upper, 1), nsmall = 1), sep = '-', collapse = NULL)

CirrvsNAFL_liver_decomp_cat_OR_tab$CirrvsNAFL_text <- paste(CirrvsNAFL_liver_decomp_cat_OR_tab$CirrvsNAFL_text, '', sep = ')', collapse = NULL)

CirrvsNAFL_liver_decomp_cat_OR_tab$var <- "liver_decomp_cat"

CirrvsNAFL_liver_decomp_cat_OR_tab <- CirrvsNAFL_liver_decomp_cat_OR_tab %>% slice(2)

CirrvsNAFL_liver_decomp_cat_OR_tab <- CirrvsNAFL_liver_decomp_cat_OR_tab %>% select(5:7)

CirrvsNAFL_Livercancer_OR <- glm(df_clean$CirrvsNAFL ~ df_clean$Livercancer + df_clean$age + df_clean$gender, family = binomial, data = df_clean)

CirrvsNAFL_Livercancer_OR_tab <- as.data.frame(confint(CirrvsNAFL_Livercancer_OR))

CirrvsNAFL_Livercancer_OR_tab$OR <- summary(CirrvsNAFL_Livercancer_OR)$coefficients[,1]

CirrvsNAFL_Livercancer_OR_tab$z_score <- summary(CirrvsNAFL_Livercancer_OR)$coefficients[,3]

CirrvsNAFL_Livercancer_OR_tab$CirrvsNAFL_pval <- 2*pnorm(-abs(CirrvsNAFL_Livercancer_OR_tab$z_score))

CirrvsNAFL_Livercancer_OR_tab <- CirrvsNAFL_Livercancer_OR_tab %>% rename(lower = c(1))

CirrvsNAFL_Livercancer_OR_tab <- CirrvsNAFL_Livercancer_OR_tab %>% rename(upper = c(2))

CirrvsNAFL_Livercancer_OR_tab$CirrvsNAFL_text <- paste(format(round(CirrvsNAFL_Livercancer_OR_tab$OR, 1), nsmall = 1), format(round(CirrvsNAFL_Livercancer_OR_tab$lower, 1), nsmall = 1), sep = ' (', collapse = NULL)

CirrvsNAFL_Livercancer_OR_tab$CirrvsNAFL_text <- paste(CirrvsNAFL_Livercancer_OR_tab$CirrvsNAFL_text, format(round(CirrvsNAFL_Livercancer_OR_tab$upper, 1), nsmall = 1), sep = '-', collapse = NULL)

CirrvsNAFL_Livercancer_OR_tab$CirrvsNAFL_text <- paste(CirrvsNAFL_Livercancer_OR_tab$CirrvsNAFL_text, '', sep = ')', collapse = NULL)

CirrvsNAFL_Livercancer_OR_tab$var <- "Livercancer"

CirrvsNAFL_Livercancer_OR_tab <- CirrvsNAFL_Livercancer_OR_tab %>% slice(2)

CirrvsNAFL_Livercancer_OR_tab <- CirrvsNAFL_Livercancer_OR_tab %>% select(5:7)

## cox ph mortality

NAFLvsCtrl_mort_demog <- coxph(Surv(mortalit, deathind) ~ NAFLvsCtrl + age + gender, data = df_clean)

NAFLvsCtrl_mort_demog_tab <- as.data.frame(confint(NAFLvsCtrl_mort_demog))

NAFLvsCtrl_mort_demog_tab$HR <- coef(NAFLvsCtrl_mort_demog)

NAFLvsCtrl_mort_demog_tab <- NAFLvsCtrl_mort_demog_tab %>% rename(lower = c(1))

NAFLvsCtrl_mort_demog_tab <- NAFLvsCtrl_mort_demog_tab %>% rename(upper = c(2))

NAFLvsCtrl_mort_demog_tab$HR <- exp(NAFLvsCtrl_mort_demog_tab$HR)

NAFLvsCtrl_mort_demog_tab$lower <- exp(NAFLvsCtrl_mort_demog_tab$lower)

NAFLvsCtrl_mort_demog_tab$upper <- exp(NAFLvsCtrl_mort_demog_tab$upper)

NAFLvsCtrl_mort_demog_tab$z_score <- summary(NAFLvsCtrl_mort_demog)$coefficients[,4]

NAFLvsCtrl_mort_demog_tab$NAFLvsCtrl_pval <- 2*pnorm(-abs(NAFLvsCtrl_mort_demog_tab$z_score))

NAFLvsCtrl_mort_demog_tab$NAFLvsCtrl_text <- paste(format(round(NAFLvsCtrl_mort_demog_tab$HR, 1), nsmall = 1), format(round(NAFLvsCtrl_mort_demog_tab$lower, 1), nsmall = 1), sep = ' (', collapse = NULL)

NAFLvsCtrl_mort_demog_tab$NAFLvsCtrl_text <- paste(NAFLvsCtrl_mort_demog_tab$NAFLvsCtrl_text, format(round(NAFLvsCtrl_mort_demog_tab$upper, 1), nsmall = 1), sep = '-', collapse = NULL)

NAFLvsCtrl_mort_demog_tab$NAFLvsCtrl_text <- paste(NAFLvsCtrl_mort_demog_tab$NAFLvsCtrl_text, '', sep = ')', collapse = NULL)

NAFLvsCtrl_mort_demog_tab$var <- "mort_demog"

NAFLvsCtrl_mort_demog_tab <- NAFLvsCtrl_mort_demog_tab %>% slice(1)

NAFLvsCtrl_mort_demog_tab <- NAFLvsCtrl_mort_demog_tab %>% select(5:7)

NAFLvsCtrl_mort_CVD <- coxph(Surv(mortalit, deathind) ~ NAFLvsCtrl + age + gender + T2DM + Obesity + HYPERLIPIDAEMIA + HEARTFAILURE + AF + CKD + IHD + MI + ISCHAEMICSTROKE + hyperten + PVD, data = df_clean)

NAFLvsCtrl_mort_CVD_tab <- as.data.frame(confint(NAFLvsCtrl_mort_CVD))

NAFLvsCtrl_mort_CVD_tab$HR <- coef(NAFLvsCtrl_mort_CVD)

NAFLvsCtrl_mort_CVD_tab <- NAFLvsCtrl_mort_CVD_tab %>% rename(lower = c(1))

NAFLvsCtrl_mort_CVD_tab <- NAFLvsCtrl_mort_CVD_tab %>% rename(upper = c(2))

NAFLvsCtrl_mort_CVD_tab$HR <- exp(NAFLvsCtrl_mort_CVD_tab$HR)

NAFLvsCtrl_mort_CVD_tab$lower <- exp(NAFLvsCtrl_mort_CVD_tab$lower)

NAFLvsCtrl_mort_CVD_tab$upper <- exp(NAFLvsCtrl_mort_CVD_tab$upper)

NAFLvsCtrl_mort_CVD_tab$z_score <- summary(NAFLvsCtrl_mort_CVD)$coefficients[,4]

NAFLvsCtrl_mort_CVD_tab$NAFLvsCtrl_pval <- 2*pnorm(-abs(NAFLvsCtrl_mort_CVD_tab$z_score))

NAFLvsCtrl_mort_CVD_tab$NAFLvsCtrl_text <- paste(format(round(NAFLvsCtrl_mort_CVD_tab$HR, 1), nsmall = 1), format(round(NAFLvsCtrl_mort_CVD_tab$lower, 1), nsmall = 1), sep = ' (', collapse = NULL)

NAFLvsCtrl_mort_CVD_tab$NAFLvsCtrl_text <- paste(NAFLvsCtrl_mort_CVD_tab$NAFLvsCtrl_text, format(round(NAFLvsCtrl_mort_CVD_tab$upper, 1), nsmall = 1), sep = '-', collapse = NULL)

NAFLvsCtrl_mort_CVD_tab$NAFLvsCtrl_text <- paste(NAFLvsCtrl_mort_CVD_tab$NAFLvsCtrl_text, '', sep = ')', collapse = NULL)

NAFLvsCtrl_mort_CVD_tab$var <- "mort_CVD"

NAFLvsCtrl_mort_CVD_tab <- NAFLvsCtrl_mort_CVD_tab %>% slice(1)

NAFLvsCtrl_mort_CVD_tab <- NAFLvsCtrl_mort_CVD_tab %>% select(5:7)

NAFLvsCtrl_mort_liver <- coxph(Surv(mortalit, deathind) ~ NAFLvsCtrl + age + gender + Livercancer + liver_decomp_cat, data = df_clean)

NAFLvsCtrl_mort_liver_tab <- as.data.frame(confint(NAFLvsCtrl_mort_liver))

NAFLvsCtrl_mort_liver_tab$HR <- coef(NAFLvsCtrl_mort_liver)

NAFLvsCtrl_mort_liver_tab <- NAFLvsCtrl_mort_liver_tab %>% rename(lower = c(1))

NAFLvsCtrl_mort_liver_tab <- NAFLvsCtrl_mort_liver_tab %>% rename(upper = c(2))

NAFLvsCtrl_mort_liver_tab$HR <- exp(NAFLvsCtrl_mort_liver_tab$HR)

NAFLvsCtrl_mort_liver_tab$lower <- exp(NAFLvsCtrl_mort_liver_tab$lower)

NAFLvsCtrl_mort_liver_tab$upper <- exp(NAFLvsCtrl_mort_liver_tab$upper)

NAFLvsCtrl_mort_liver_tab$z_score <- summary(NAFLvsCtrl_mort_liver)$coefficients[,4]

NAFLvsCtrl_mort_liver_tab$NAFLvsCtrl_pval <- 2*pnorm(-abs(NAFLvsCtrl_mort_liver_tab$z_score))

NAFLvsCtrl_mort_liver_tab$NAFLvsCtrl_text <- paste(format(round(NAFLvsCtrl_mort_liver_tab$HR, 1), nsmall = 1), format(round(NAFLvsCtrl_mort_liver_tab$lower, 1), nsmall = 1), sep = ' (', collapse = NULL)

NAFLvsCtrl_mort_liver_tab$NAFLvsCtrl_text <- paste(NAFLvsCtrl_mort_liver_tab$NAFLvsCtrl_text, format(round(NAFLvsCtrl_mort_liver_tab$upper, 1), nsmall = 1), sep = '-', collapse = NULL)

NAFLvsCtrl_mort_liver_tab$NAFLvsCtrl_text <- paste(NAFLvsCtrl_mort_liver_tab$NAFLvsCtrl_text, '', sep = ')', collapse = NULL)

NAFLvsCtrl_mort_liver_tab$var <- "mort_liver"

NAFLvsCtrl_mort_liver_tab <- NAFLvsCtrl_mort_liver_tab %>% slice(1)

NAFLvsCtrl_mort_liver_tab <- NAFLvsCtrl_mort_liver_tab %>% select(5:7)

NAFLvsCtrl_mort_CVDLiv <- coxph(Surv(mortalit, deathind) ~ NAFLvsCtrl + age + gender + T2DM + Obesity + HYPERLIPIDAEMIA + HEARTFAILURE + AF + CKD + IHD + MI + ISCHAEMICSTROKE + hyperten + PVD + Livercancer + liver_decomp_cat, data = df_clean)

NAFLvsCtrl_mort_CVDLiv_tab <- as.data.frame(confint(NAFLvsCtrl_mort_CVDLiv))

NAFLvsCtrl_mort_CVDLiv_tab$HR <- coef(NAFLvsCtrl_mort_CVDLiv)

NAFLvsCtrl_mort_CVDLiv_tab <- NAFLvsCtrl_mort_CVDLiv_tab %>% rename(lower = c(1))

NAFLvsCtrl_mort_CVDLiv_tab <- NAFLvsCtrl_mort_CVDLiv_tab %>% rename(upper = c(2))

NAFLvsCtrl_mort_CVDLiv_tab$HR <- exp(NAFLvsCtrl_mort_CVDLiv_tab$HR)

NAFLvsCtrl_mort_CVDLiv_tab$lower <- exp(NAFLvsCtrl_mort_CVDLiv_tab$lower)

NAFLvsCtrl_mort_CVDLiv_tab$upper <- exp(NAFLvsCtrl_mort_CVDLiv_tab$upper)

NAFLvsCtrl_mort_CVDLiv_tab$z_score <- summary(NAFLvsCtrl_mort_CVDLiv)$coefficients[,4]

NAFLvsCtrl_mort_CVDLiv_tab$NAFLvsCtrl_pval <- 2*pnorm(-abs(NAFLvsCtrl_mort_CVDLiv_tab$z_score))

NAFLvsCtrl_mort_CVDLiv_tab$NAFLvsCtrl_text <- paste(format(round(NAFLvsCtrl_mort_CVDLiv_tab$HR, 1), nsmall = 1), format(round(NAFLvsCtrl_mort_CVDLiv_tab$lower, 1), nsmall = 1), sep = ' (', collapse = NULL)

NAFLvsCtrl_mort_CVDLiv_tab$NAFLvsCtrl_text <- paste(NAFLvsCtrl_mort_CVDLiv_tab$NAFLvsCtrl_text, format(round(NAFLvsCtrl_mort_CVDLiv_tab$upper, 1), nsmall = 1), sep = '-', collapse = NULL)

NAFLvsCtrl_mort_CVDLiv_tab$NAFLvsCtrl_text <- paste(NAFLvsCtrl_mort_CVDLiv_tab$NAFLvsCtrl_text, '', sep = ')', collapse = NULL)

NAFLvsCtrl_mort_CVDLiv_tab$var <- "mort_CVDLiv"

NAFLvsCtrl_mort_CVDLiv_tab <- NAFLvsCtrl_mort_CVDLiv_tab %>% slice(1)

NAFLvsCtrl_mort_CVDLiv_tab <- NAFLvsCtrl_mort_CVDLiv_tab %>% select(5:7)

CirrvsCtrl_mort_demog <- coxph(Surv(mortalit, deathind) ~ CirrvsCtrl + age + gender, data = df_clean)

CirrvsCtrl_mort_demog_tab <- as.data.frame(confint(CirrvsCtrl_mort_demog))

CirrvsCtrl_mort_demog_tab$HR <- coef(CirrvsCtrl_mort_demog)

CirrvsCtrl_mort_demog_tab <- CirrvsCtrl_mort_demog_tab %>% rename(lower = c(1))

CirrvsCtrl_mort_demog_tab <- CirrvsCtrl_mort_demog_tab %>% rename(upper = c(2))

CirrvsCtrl_mort_demog_tab$HR <- exp(CirrvsCtrl_mort_demog_tab$HR)

CirrvsCtrl_mort_demog_tab$lower <- exp(CirrvsCtrl_mort_demog_tab$lower)

CirrvsCtrl_mort_demog_tab$upper <- exp(CirrvsCtrl_mort_demog_tab$upper)

CirrvsCtrl_mort_demog_tab$z_score <- summary(CirrvsCtrl_mort_demog)$coefficients[,4]

CirrvsCtrl_mort_demog_tab$CirrvsCtrl_pval <- 2*pnorm(-abs(CirrvsCtrl_mort_demog_tab$z_score))

CirrvsCtrl_mort_demog_tab$CirrvsCtrl_text <- paste(format(round(CirrvsCtrl_mort_demog_tab$HR, 1), nsmall = 1), format(round(CirrvsCtrl_mort_demog_tab$lower, 1), nsmall = 1), sep = ' (', collapse = NULL)

CirrvsCtrl_mort_demog_tab$CirrvsCtrl_text <- paste(CirrvsCtrl_mort_demog_tab$CirrvsCtrl_text, format(round(CirrvsCtrl_mort_demog_tab$upper, 1), nsmall = 1), sep = '-', collapse = NULL)

CirrvsCtrl_mort_demog_tab$CirrvsCtrl_text <- paste(CirrvsCtrl_mort_demog_tab$CirrvsCtrl_text, '', sep = ')', collapse = NULL)

CirrvsCtrl_mort_demog_tab$var <- "mort_demog"

CirrvsCtrl_mort_demog_tab <- CirrvsCtrl_mort_demog_tab %>% slice(1)

CirrvsCtrl_mort_demog_tab <- CirrvsCtrl_mort_demog_tab %>% select(5:7)

CirrvsCtrl_mort_CVD <- coxph(Surv(mortalit, deathind) ~ CirrvsCtrl + age + gender + T2DM + Obesity + HYPERLIPIDAEMIA + HEARTFAILURE + AF + CKD + IHD + MI + ISCHAEMICSTROKE + hyperten + PVD, data = df_clean)

CirrvsCtrl_mort_CVD_tab <- as.data.frame(confint(CirrvsCtrl_mort_CVD))

CirrvsCtrl_mort_CVD_tab$HR <- coef(CirrvsCtrl_mort_CVD)

CirrvsCtrl_mort_CVD_tab <- CirrvsCtrl_mort_CVD_tab %>% rename(lower = c(1))

CirrvsCtrl_mort_CVD_tab <- CirrvsCtrl_mort_CVD_tab %>% rename(upper = c(2))

CirrvsCtrl_mort_CVD_tab$HR <- exp(CirrvsCtrl_mort_CVD_tab$HR)

CirrvsCtrl_mort_CVD_tab$lower <- exp(CirrvsCtrl_mort_CVD_tab$lower)

CirrvsCtrl_mort_CVD_tab$upper <- exp(CirrvsCtrl_mort_CVD_tab$upper)

CirrvsCtrl_mort_CVD_tab$z_score <- summary(CirrvsCtrl_mort_CVD)$coefficients[,4]

CirrvsCtrl_mort_CVD_tab$CirrvsCtrl_pval <- 2*pnorm(-abs(CirrvsCtrl_mort_CVD_tab$z_score))

CirrvsCtrl_mort_CVD_tab$CirrvsCtrl_text <- paste(format(round(CirrvsCtrl_mort_CVD_tab$HR, 1), nsmall = 1), format(round(CirrvsCtrl_mort_CVD_tab$lower, 1), nsmall = 1), sep = ' (', collapse = NULL)

CirrvsCtrl_mort_CVD_tab$CirrvsCtrl_text <- paste(CirrvsCtrl_mort_CVD_tab$CirrvsCtrl_text, format(round(CirrvsCtrl_mort_CVD_tab$upper, 1), nsmall = 1), sep = '-', collapse = NULL)

CirrvsCtrl_mort_CVD_tab$CirrvsCtrl_text <- paste(CirrvsCtrl_mort_CVD_tab$CirrvsCtrl_text, '', sep = ')', collapse = NULL)

CirrvsCtrl_mort_CVD_tab$var <- "mort_CVD"

CirrvsCtrl_mort_CVD_tab <- CirrvsCtrl_mort_CVD_tab %>% slice(1)

CirrvsCtrl_mort_CVD_tab <- CirrvsCtrl_mort_CVD_tab %>% select(5:7)

CirrvsCtrl_mort_liver <- coxph(Surv(mortalit, deathind) ~ CirrvsCtrl + age + gender + Livercancer + liver_decomp_cat, data = df_clean)

CirrvsCtrl_mort_liver_tab <- as.data.frame(confint(CirrvsCtrl_mort_liver))

CirrvsCtrl_mort_liver_tab$HR <- coef(CirrvsCtrl_mort_liver)

CirrvsCtrl_mort_liver_tab <- CirrvsCtrl_mort_liver_tab %>% rename(lower = c(1))

CirrvsCtrl_mort_liver_tab <- CirrvsCtrl_mort_liver_tab %>% rename(upper = c(2))

CirrvsCtrl_mort_liver_tab$HR <- exp(CirrvsCtrl_mort_liver_tab$HR)

CirrvsCtrl_mort_liver_tab$lower <- exp(CirrvsCtrl_mort_liver_tab$lower)

CirrvsCtrl_mort_liver_tab$upper <- exp(CirrvsCtrl_mort_liver_tab$upper)

CirrvsCtrl_mort_liver_tab$z_score <- summary(CirrvsCtrl_mort_liver)$coefficients[,4]

CirrvsCtrl_mort_liver_tab$CirrvsCtrl_pval <- 2*pnorm(-abs(CirrvsCtrl_mort_liver_tab$z_score))

CirrvsCtrl_mort_liver_tab$CirrvsCtrl_text <- paste(format(round(CirrvsCtrl_mort_liver_tab$HR, 1), nsmall = 1), format(round(CirrvsCtrl_mort_liver_tab$lower, 1), nsmall = 1), sep = ' (', collapse = NULL)

CirrvsCtrl_mort_liver_tab$CirrvsCtrl_text <- paste(CirrvsCtrl_mort_liver_tab$CirrvsCtrl_text, format(round(CirrvsCtrl_mort_liver_tab$upper, 1), nsmall = 1), sep = '-', collapse = NULL)

CirrvsCtrl_mort_liver_tab$CirrvsCtrl_text <- paste(CirrvsCtrl_mort_liver_tab$CirrvsCtrl_text, '', sep = ')', collapse = NULL)

CirrvsCtrl_mort_liver_tab$var <- "mort_liver"

CirrvsCtrl_mort_liver_tab <- CirrvsCtrl_mort_liver_tab %>% slice(1)

CirrvsCtrl_mort_liver_tab <- CirrvsCtrl_mort_liver_tab %>% select(5:7)

CirrvsCtrl_mort_CVDLiv <- coxph(Surv(mortalit, deathind) ~ CirrvsCtrl + age + gender + T2DM + Obesity + HYPERLIPIDAEMIA + HEARTFAILURE + AF + CKD + IHD + MI + ISCHAEMICSTROKE + hyperten + PVD + Livercancer + liver_decomp_cat, data = df_clean)

CirrvsCtrl_mort_CVDLiv_tab <- as.data.frame(confint(CirrvsCtrl_mort_CVDLiv))

CirrvsCtrl_mort_CVDLiv_tab$HR <- coef(CirrvsCtrl_mort_CVDLiv)

CirrvsCtrl_mort_CVDLiv_tab <- CirrvsCtrl_mort_CVDLiv_tab %>% rename(lower = c(1))

CirrvsCtrl_mort_CVDLiv_tab <- CirrvsCtrl_mort_CVDLiv_tab %>% rename(upper = c(2))

CirrvsCtrl_mort_CVDLiv_tab$HR <- exp(CirrvsCtrl_mort_CVDLiv_tab$HR)

CirrvsCtrl_mort_CVDLiv_tab$lower <- exp(CirrvsCtrl_mort_CVDLiv_tab$lower)

CirrvsCtrl_mort_CVDLiv_tab$upper <- exp(CirrvsCtrl_mort_CVDLiv_tab$upper)

CirrvsCtrl_mort_CVDLiv_tab$z_score <- summary(CirrvsCtrl_mort_CVDLiv)$coefficients[,4]

CirrvsCtrl_mort_CVDLiv_tab$CirrvsCtrl_pval <- 2*pnorm(-abs(CirrvsCtrl_mort_CVDLiv_tab$z_score))

CirrvsCtrl_mort_CVDLiv_tab$CirrvsCtrl_text <- paste(format(round(CirrvsCtrl_mort_CVDLiv_tab$HR, 1), nsmall = 1), format(round(CirrvsCtrl_mort_CVDLiv_tab$lower, 1), nsmall = 1), sep = ' (', collapse = NULL)

CirrvsCtrl_mort_CVDLiv_tab$CirrvsCtrl_text <- paste(CirrvsCtrl_mort_CVDLiv_tab$CirrvsCtrl_text, format(round(CirrvsCtrl_mort_CVDLiv_tab$upper, 1), nsmall = 1), sep = '-', collapse = NULL)

CirrvsCtrl_mort_CVDLiv_tab$CirrvsCtrl_text <- paste(CirrvsCtrl_mort_CVDLiv_tab$CirrvsCtrl_text, '', sep = ')', collapse = NULL)

CirrvsCtrl_mort_CVDLiv_tab$var <- "mort_CVDLiv"

CirrvsCtrl_mort_CVDLiv_tab <- CirrvsCtrl_mort_CVDLiv_tab %>% slice(1)

CirrvsCtrl_mort_CVDLiv_tab <- CirrvsCtrl_mort_CVDLiv_tab %>% select(5:7)

CirrvsNAFL_mort_demog <- coxph(Surv(mortalit, deathind) ~ CirrvsNAFL + age + gender, data = df_clean)

CirrvsNAFL_mort_demog_tab <- as.data.frame(confint(CirrvsNAFL_mort_demog))

CirrvsNAFL_mort_demog_tab$HR <- coef(CirrvsNAFL_mort_demog)

CirrvsNAFL_mort_demog_tab <- CirrvsNAFL_mort_demog_tab %>% rename(lower = c(1))

CirrvsNAFL_mort_demog_tab <- CirrvsNAFL_mort_demog_tab %>% rename(upper = c(2))

CirrvsNAFL_mort_demog_tab$HR <- exp(CirrvsNAFL_mort_demog_tab$HR)

CirrvsNAFL_mort_demog_tab$lower <- exp(CirrvsNAFL_mort_demog_tab$lower)

CirrvsNAFL_mort_demog_tab$upper <- exp(CirrvsNAFL_mort_demog_tab$upper)

CirrvsNAFL_mort_demog_tab$z_score <- summary(CirrvsNAFL_mort_demog)$coefficients[,4]

CirrvsNAFL_mort_demog_tab$CirrvsNAFL_pval <- 2*pnorm(-abs(CirrvsNAFL_mort_demog_tab$z_score))

CirrvsNAFL_mort_demog_tab$CirrvsNAFL_text <- paste(format(round(CirrvsNAFL_mort_demog_tab$HR, 1), nsmall = 1), format(round(CirrvsNAFL_mort_demog_tab$lower, 1), nsmall = 1), sep = ' (', collapse = NULL)

CirrvsNAFL_mort_demog_tab$CirrvsNAFL_text <- paste(CirrvsNAFL_mort_demog_tab$CirrvsNAFL_text, format(round(CirrvsNAFL_mort_demog_tab$upper, 1), nsmall = 1), sep = '-', collapse = NULL)

CirrvsNAFL_mort_demog_tab$CirrvsNAFL_text <- paste(CirrvsNAFL_mort_demog_tab$CirrvsNAFL_text, '', sep = ')', collapse = NULL)

CirrvsNAFL_mort_demog_tab$var <- "mort_demog"

CirrvsNAFL_mort_demog_tab <- CirrvsNAFL_mort_demog_tab %>% slice(1)

CirrvsNAFL_mort_demog_tab <- CirrvsNAFL_mort_demog_tab %>% select(5:7)

CirrvsNAFL_mort_CVD <- coxph(Surv(mortalit, deathind) ~ CirrvsNAFL + age + gender + T2DM + Obesity + HYPERLIPIDAEMIA + HEARTFAILURE + AF + CKD + IHD + MI + ISCHAEMICSTROKE + hyperten + PVD, data = df_clean)

CirrvsNAFL_mort_CVD_tab <- as.data.frame(confint(CirrvsNAFL_mort_CVD))

CirrvsNAFL_mort_CVD_tab$HR <- coef(CirrvsNAFL_mort_CVD)

CirrvsNAFL_mort_CVD_tab <- CirrvsNAFL_mort_CVD_tab %>% rename(lower = c(1))

CirrvsNAFL_mort_CVD_tab <- CirrvsNAFL_mort_CVD_tab %>% rename(upper = c(2))

CirrvsNAFL_mort_CVD_tab$HR <- exp(CirrvsNAFL_mort_CVD_tab$HR)

CirrvsNAFL_mort_CVD_tab$lower <- exp(CirrvsNAFL_mort_CVD_tab$lower)

CirrvsNAFL_mort_CVD_tab$upper <- exp(CirrvsNAFL_mort_CVD_tab$upper)

CirrvsNAFL_mort_CVD_tab$z_score <- summary(CirrvsNAFL_mort_CVD)$coefficients[,4]

CirrvsNAFL_mort_CVD_tab$CirrvsNAFL_pval <- 2*pnorm(-abs(CirrvsNAFL_mort_CVD_tab$z_score))

CirrvsNAFL_mort_CVD_tab$CirrvsNAFL_text <- paste(format(round(CirrvsNAFL_mort_CVD_tab$HR, 1), nsmall = 1), format(round(CirrvsNAFL_mort_CVD_tab$lower, 1), nsmall = 1), sep = ' (', collapse = NULL)

CirrvsNAFL_mort_CVD_tab$CirrvsNAFL_text <- paste(CirrvsNAFL_mort_CVD_tab$CirrvsNAFL_text, format(round(CirrvsNAFL_mort_CVD_tab$upper, 1), nsmall = 1), sep = '-', collapse = NULL)

CirrvsNAFL_mort_CVD_tab$CirrvsNAFL_text <- paste(CirrvsNAFL_mort_CVD_tab$CirrvsNAFL_text, '', sep = ')', collapse = NULL)

CirrvsNAFL_mort_CVD_tab$var <- "mort_CVD"

CirrvsNAFL_mort_CVD_tab <- CirrvsNAFL_mort_CVD_tab %>% slice(1)

CirrvsNAFL_mort_CVD_tab <- CirrvsNAFL_mort_CVD_tab %>% select(5:7)

CirrvsNAFL_mort_liver <- coxph(Surv(mortalit, deathind) ~ CirrvsNAFL + age + gender + Livercancer + liver_decomp_cat, data = df_clean)

CirrvsNAFL_mort_liver_tab <- as.data.frame(confint(CirrvsNAFL_mort_liver))

CirrvsNAFL_mort_liver_tab$HR <- coef(CirrvsNAFL_mort_liver)

CirrvsNAFL_mort_liver_tab <- CirrvsNAFL_mort_liver_tab %>% rename(lower = c(1))

CirrvsNAFL_mort_liver_tab <- CirrvsNAFL_mort_liver_tab %>% rename(upper = c(2))

CirrvsNAFL_mort_liver_tab$HR <- exp(CirrvsNAFL_mort_liver_tab$HR)

CirrvsNAFL_mort_liver_tab$lower <- exp(CirrvsNAFL_mort_liver_tab$lower)

CirrvsNAFL_mort_liver_tab$upper <- exp(CirrvsNAFL_mort_liver_tab$upper)

CirrvsNAFL_mort_liver_tab$z_score <- summary(CirrvsNAFL_mort_liver)$coefficients[,4]

CirrvsNAFL_mort_liver_tab$CirrvsNAFL_pval <- 2*pnorm(-abs(CirrvsNAFL_mort_liver_tab$z_score))

CirrvsNAFL_mort_liver_tab$CirrvsNAFL_text <- paste(format(round(CirrvsNAFL_mort_liver_tab$HR, 1), nsmall = 1), format(round(CirrvsNAFL_mort_liver_tab$lower, 1), nsmall = 1), sep = ' (', collapse = NULL)

CirrvsNAFL_mort_liver_tab$CirrvsNAFL_text <- paste(CirrvsNAFL_mort_liver_tab$CirrvsNAFL_text, format(round(CirrvsNAFL_mort_liver_tab$upper, 1), nsmall = 1), sep = '-', collapse = NULL)

CirrvsNAFL_mort_liver_tab$CirrvsNAFL_text <- paste(CirrvsNAFL_mort_liver_tab$CirrvsNAFL_text, '', sep = ')', collapse = NULL)

CirrvsNAFL_mort_liver_tab$var <- "mort_liver"

CirrvsNAFL_mort_liver_tab <- CirrvsNAFL_mort_liver_tab %>% slice(1)

CirrvsNAFL_mort_liver_tab <- CirrvsNAFL_mort_liver_tab %>% select(5:7)

CirrvsNAFL_mort_CVDLiv <- coxph(Surv(mortalit, deathind) ~ CirrvsNAFL + age + gender + T2DM + Obesity + HYPERLIPIDAEMIA + HEARTFAILURE + AF + CKD + IHD + MI + ISCHAEMICSTROKE + hyperten + PVD + Livercancer + liver_decomp_cat, data = df_clean)

CirrvsNAFL_mort_CVDLiv_tab <- as.data.frame(confint(CirrvsNAFL_mort_CVDLiv))

CirrvsNAFL_mort_CVDLiv_tab$HR <- coef(CirrvsNAFL_mort_CVDLiv)

CirrvsNAFL_mort_CVDLiv_tab <- CirrvsNAFL_mort_CVDLiv_tab %>% rename(lower = c(1))

CirrvsNAFL_mort_CVDLiv_tab <- CirrvsNAFL_mort_CVDLiv_tab %>% rename(upper = c(2))

CirrvsNAFL_mort_CVDLiv_tab$HR <- exp(CirrvsNAFL_mort_CVDLiv_tab$HR)

CirrvsNAFL_mort_CVDLiv_tab$lower <- exp(CirrvsNAFL_mort_CVDLiv_tab$lower)

CirrvsNAFL_mort_CVDLiv_tab$upper <- exp(CirrvsNAFL_mort_CVDLiv_tab$upper)

CirrvsNAFL_mort_CVDLiv_tab$z_score <- summary(CirrvsNAFL_mort_CVDLiv)$coefficients[,4]

CirrvsNAFL_mort_CVDLiv_tab$CirrvsNAFL_pval <- 2*pnorm(-abs(CirrvsNAFL_mort_CVDLiv_tab$z_score))

CirrvsNAFL_mort_CVDLiv_tab$CirrvsNAFL_text <- paste(format(round(CirrvsNAFL_mort_CVDLiv_tab$HR, 1), nsmall = 1), format(round(CirrvsNAFL_mort_CVDLiv_tab$lower, 1), nsmall = 1), sep = ' (', collapse = NULL)

CirrvsNAFL_mort_CVDLiv_tab$CirrvsNAFL_text <- paste(CirrvsNAFL_mort_CVDLiv_tab$CirrvsNAFL_text, format(round(CirrvsNAFL_mort_CVDLiv_tab$upper, 1), nsmall = 1), sep = '-', collapse = NULL)

CirrvsNAFL_mort_CVDLiv_tab$CirrvsNAFL_text <- paste(CirrvsNAFL_mort_CVDLiv_tab$CirrvsNAFL_text, '', sep = ')', collapse = NULL)

CirrvsNAFL_mort_CVDLiv_tab$var <- "mort_CVDLiv"

CirrvsNAFL_mort_CVDLiv_tab <- CirrvsNAFL_mort_CVDLiv_tab %>% slice(1)

CirrvsNAFL_mort_CVDLiv_tab <- CirrvsNAFL_mort_CVDLiv_tab %>% select(5:7)

Livercancer_OR_sumtab2 <- merge(NAFLvsCtrl_Livercancer_OR_tab, CirrvsCtrl_Livercancer_OR_tab, by="var")

Livercancer_OR_sumtab2 <- merge(Livercancer_OR_sumtab2, CirrvsNAFL_Livercancer_OR_tab, by="var")

Livercancer_OR_sumtab2 <- merge(Livercancer_OR_sumtab2, CirrvsNASH_Livercancer_OR_tab, by="var")

Livercancer_OR_sumtab2 <- merge(Livercancer_OR_sumtab2, NAFLvsNASH_Livercancer_OR_tab, by="var")

Livercancer_OR_sumtab2 <- merge(Livercancer_OR_sumtab2, NASHvsCtrl_Livercancer_OR_tab, by="var")

liver_decomp_cat_OR_sumtab2 <- merge(NAFLvsCtrl_liver_decomp_cat_OR_tab, CirrvsCtrl_liver_decomp_cat_OR_tab, by="var")

liver_decomp_cat_OR_sumtab2 <- merge(liver_decomp_cat_OR_sumtab2, CirrvsNAFL_liver_decomp_cat_OR_tab, by="var")

liver_decomp_cat_OR_sumtab2 <- merge(liver_decomp_cat_OR_sumtab2, CirrvsNASH_liver_decomp_cat_OR_tab, by="var")

liver_decomp_cat_OR_sumtab2 <- merge(liver_decomp_cat_OR_sumtab2, NAFLvsNASH_liver_decomp_cat_OR_tab, by="var")

liver_decomp_cat_OR_sumtab2 <- merge(liver_decomp_cat_OR_sumtab2, NASHvsCtrl_liver_decomp_cat_OR_tab, by="var")

mort_demog_sumtab2 <- merge(NAFLvsCtrl_mort_demog_tab, CirrvsCtrl_mort_demog_tab, by="var")

mort_demog_sumtab2 <- merge(mort_demog_sumtab2, CirrvsNAFL_mort_demog_tab, by="var")

mort_demog_sumtab2 <- merge(mort_demog_sumtab2, CirrvsNASH_mort_demog_tab, by="var")

mort_demog_sumtab2 <- merge(mort_demog_sumtab2, NAFLvsNASH_mort_demog_tab, by="var")

mort_demog_sumtab2 <- merge(mort_demog_sumtab2, NASHvsCtrl_mort_demog_tab, by="var")

mort_CVD_sumtab2 <- merge(NAFLvsCtrl_mort_CVD_tab, CirrvsCtrl_mort_CVD_tab, by="var")

mort_CVD_sumtab2 <- merge(mort_CVD_sumtab2, CirrvsNAFL_mort_CVD_tab, by="var")

mort_CVD_sumtab2 <- merge(mort_CVD_sumtab2, CirrvsNASH_mort_CVD_tab, by="var")

mort_CVD_sumtab2 <- merge(mort_CVD_sumtab2, NAFLvsNASH_mort_CVD_tab, by="var")

mort_CVD_sumtab2 <- merge(mort_CVD_sumtab2, NASHvsCtrl_mort_CVD_tab, by="var")

mort_liver_sumtab2 <- merge(NAFLvsCtrl_mort_liver_tab, CirrvsCtrl_mort_liver_tab, by="var")

mort_liver_sumtab2 <- merge(mort_liver_sumtab2, CirrvsNAFL_mort_liver_tab, by="var")

mort_liver_sumtab2 <- merge(mort_liver_sumtab2, CirrvsNASH_mort_liver_tab, by="var")

mort_liver_sumtab2 <- merge(mort_liver_sumtab2, NAFLvsNASH_mort_liver_tab, by="var")

mort_liver_sumtab2 <- merge(mort_liver_sumtab2, NASHvsCtrl_mort_liver_tab, by="var")

mort_CVDLiv_sumtab2 <- merge(NAFLvsCtrl_mort_CVDLiv_tab, CirrvsCtrl_mort_CVDLiv_tab, by="var")

mort_CVDLiv_sumtab2 <- merge(mort_CVDLiv_sumtab2, CirrvsNAFL_mort_CVDLiv_tab, by="var")

mort_CVDLiv_sumtab2 <- merge(mort_CVDLiv_sumtab2, CirrvsNASH_mort_CVDLiv_tab, by="var")

mort_CVDLiv_sumtab2 <- merge(mort_CVDLiv_sumtab2, NAFLvsNASH_mort_CVDLiv_tab, by="var")

mort_CVDLiv_sumtab2 <- merge(mort_CVDLiv_sumtab2, NASHvsCtrl_mort_CVDLiv_tab, by="var")

OR_sumtab2 <- rbind(liver_decomp_cat_OR_sumtab2, Livercancer_OR_sumtab2, mort_demog_sumtab2, mort_CVD_sumtab2, mort_liver_sumtab2, mort_CVDLiv_sumtab2)

OR_sumtab2$NAFLvsCtrl_qval <- p.adjust(OR_sumtab2$NAFLvsCtrl_pval, method = "BH")

OR_sumtab2$CirrvsCtrl_qval <- p.adjust(OR_sumtab2$CirrvsCtrl_pval, method = "BH")

OR_sumtab2$CirrvsNAFL_qval <- p.adjust(OR_sumtab2$CirrvsNAFL_pval, method = "BH")

OR_sumtab2$CirrvsNASH_qval <- p.adjust(OR_sumtab2$CirrvsNASH_pval, method = "BH")

OR_sumtab2$NAFLvsNASH_qval <- p.adjust(OR_sumtab2$NAFLvsNASH_pval, method = "BH")

OR_sumtab2$NASHvsCtrl_qval <- p.adjust(OR_sumtab2$NASHvsCtrl_pval, method = "BH")

OR_sumtab2$NAFLvsCtrl_qval <- ifelse(OR_sumtab2$NAFLvsCtrl_qval <.01, format(OR_sumtab2$NAFLvsCtrl_qval, scientific=T, digits=2), format(round(OR_sumtab2$NAFLvsCtrl_qval, 2), nsmall = 2))

OR_sumtab2$CirrvsCtrl_qval <- ifelse(OR_sumtab2$CirrvsCtrl_qval <.01, format(OR_sumtab2$CirrvsCtrl_qval, scientific=T, digits=2), format(round(OR_sumtab2$CirrvsCtrl_qval, 2), nsmall = 2))

OR_sumtab2$CirrvsNAFL_qval <- ifelse(OR_sumtab2$CirrvsNAFL_qval <.01, format(OR_sumtab2$CirrvsNAFL_qval, scientific=T, digits=2), format(round(OR_sumtab2$CirrvsNAFL_qval, 2), nsmall = 2))

OR_sumtab2$CirrvsNASH_qval <- ifelse(OR_sumtab2$CirrvsNASH_qval <.01, format(OR_sumtab2$CirrvsNASH_qval, scientific=T, digits=2), format(round(OR_sumtab2$CirrvsNASH_qval, 2), nsmall = 2))

OR_sumtab2$NAFLvsNASH_qval <- ifelse(OR_sumtab2$NAFLvsNASH_qval <.01, format(OR_sumtab2$NAFLvsNASH_qval, scientific=T, digits=2), format(round(OR_sumtab2$NAFLvsNASH_qval, 2), nsmall = 2))

OR_sumtab2$NASHvsCtrl_qval <- ifelse(OR_sumtab2$NASHvsCtrl_qval <.01, format(OR_sumtab2$NASHvsCtrl_qval, scientific=T, digits=2), format(round(OR_sumtab2$NASHvsCtrl_qval, 2), nsmall = 2))

write.table(OR_sumtab2, file="OR_sumtab2.csv", sep=",")
